# Integration of genome-wide association studies, metabolomics, and transcriptomics reveals phenolic acid- and flavonoid-associated genes and their regulatory elements under drought stress in rapeseed flowers

**DOI:** 10.3389/fpls.2023.1249142

**Published:** 2024-01-11

**Authors:** Maryam Salami, Bahram Heidari, Jacqueline Batley, Jin Wang, Xiao-Li Tan, Christopher Richards, Helin Tan

**Affiliations:** ^1^ Department of Plant Production and Genetics, School of Agriculture, Shiraz University, Shiraz, Iran; ^2^ School of Biological Sciences, University of Western Australia, Perth, WA, Australia; ^3^ School of Life Sciences, Jiangsu University, Zhenjiang, China; ^4^ United States Department of Agriculture (USDA) Agricultural Research Service (ARS), National Laboratory for Genetic Resources Preservation, Fort Collins, CO, United States; ^5^ State Key Laboratory of Crop Genetics and Germplasm Enhancement, Nanjing Agricultural University, Nanjing, China

**Keywords:** *Brassica napus* L., candidate gene, GWAS, haplotype, phytochemicals, SNP, transcriptomic

## Abstract

**Introduction:**

Biochemical and metabolic processes help plants tolerate the adverse effects of drought. In plants accumulating bioactive compounds, understanding the genetic control of the biosynthesis of biochemical pathways helps the discovery of candidate gene (CG)–metabolite relationships.

**Methods:**

The metabolic profile of flowers in 119 rapeseed (*Brassica napus*) accessions was assessed over two irrigation treatments, one a well-watered (WW) condition and the other a drought stress (DS) regime. We integrated information gained from 52,157 single-nucleotide polymorphism (SNP) markers, metabolites, and transcriptomes to identify linked SNPs and CGs responsible for the genetic control of flower phenolic compounds and regulatory elements.

**Results:**

In a genome-wide association study (GWAS), of the SNPs tested, 29,310 SNPs were qualified to assess the population structure and linkage disequilibrium (LD), of which several SNPs for radical scavenging activity (RSA) and total flavanol content (TFLC) were common between the two irrigation conditions and pleiotropic SNPs were found for chlorogenic and coumaric acids content. The principal component analysis (PCA) and stepwise regression showed that chlorogenic acid and epicatechin in WW and myricetin in DS conditions were the most important components for RSA. The hierarchical cluster analysis (HCA) showed that vanillic acid, myricetin, gallic acid, and catechin were closely associated in both irrigation conditions. Analysis of GWAS showed that 60 CGs were identified, of which 18 were involved in stress-induced pathways, phenylpropanoid pathway, and flavonoid modifications. Of the CGs, *PAL1*, *CHI*, *UGT89B1*, *FLS3*, *CCR1*, and *CYP75B137* contributed to flavonoid biosynthetic pathways. The results of RNA sequencing (RNA-seq) revealed that the transcript levels of *PAL*, *CHI*, and *CYP75B137* known as early flavonoid biosynthesis-related genes and *FLS3*, *CCR1*, and *UGT89B1* related to the later stages were increased during drought conditions. The transcription factors (TFs) *NAC035* and *ERF119* related to flavonoids and phenolic acids were upregulated under drought conditions.

**Discussion:**

These findings expand our knowledge on the response mechanisms to DS, particularly regarding the regulation of key phenolic biosynthetic genes in rapeseed. Our data also provided specific linked SNPs for marker-assisted selection (MAS) programs and CGs as resources toward realizing metabolomics-associated breeding of rapeseed.

## Introduction

*Brassica napus* L. (2n = 4x = 38, AACC), commonly known as rapeseed or canola, is the most important oil crop of the *Brassica* genus and is widely grown globally (Chalhoub et al., 2014). *B*. *napus* with high oil (~50%) and protein (~25%) contents is used as fodder and human food. The leaves and flowers of rapeseed are edible and can be used for therapeutic purposes due to their diuretic, anti-scurvy, and anti-inflammatory activities (Raboanatahiry et al., 2021). Furthermore, rapeseed is rich in both nutritional and non-nutritional antioxidants and accumulates phenols, hydroxybenzoic acids, hydroxycinnamic acids, and flavonoids (flavanols, flavones, flavanones, isoflavones, and anthocyanins) at a rate of 10–30 times higher than other oilseeds (Shao et al., 2014). The growing interest in edible flowers is motivated not only by their decorative and nutraceutical properties but also because of the desire for new flavors and new opportunities for food innovation. The antioxidant power of flowers derives from their richness in generic phenolic compounds (Mikolajczak et al., 2020). Phenolic compounds (phenolic acids and flavonoids) are substantial groups of plant secondary metabolites known as markers for biotic and abiotic stress tolerance (Sharma et al., 2019). Phenolic contents and compounds and phenolic oxidizing enzymes are strongly associated with food quality (Tomas-Barberan and Espin, 2001).

The biosynthesis of flavonoids is regulated by various signaling molecules, biosynthetic enzymes, transcription factors (TFs), and phytohormonal regulators (Li et al., 2013). The flavonoid biosynthetic-related genes at early steps including the key enzymes chalcone synthase (CHS), chalcone isomerase (CHI), flavanone 3-hydroxylase (F3H), flavonoid 3′-hydroxylase (F3′H), and flavonoid 3′,5′-hydroxylase (F3′5′H) are responsible for producing common precursors (Zhao, 2015). Then, the later-step biosynthetic enzymes dihydroflavonol-4-reductase (DFR), flavonol synthase (FLS), leucoanthocyanidin reductase (LAR), leucoanthocyanidin dioxygenase (LDOX), anthocyanidin reductase (ANR), and UDP-glucose:flavonoid 3-O-glucosyltransferase (UFGT) catalyze the formation of anthocyanin and other flavonoid end products (flavonols and flavones) and phenolic acid (Han et al., 2012). Besides the effects of genes, environmental factors affect flavonoid accumulation and expression of the flavonoid pathway genes that shows the need for the evaluation of a germplasm under different environmental conditions (Ahmed et al., 2021).

Most parts of Iran’s agricultural areas suffer from water scarcity due to low rainfall and poor irrigation systems, which are coincident with flowering and seed filling of rapeseed (Kalantarahmadi and Shoushi Dezfouli, 2021). It has been shown that rapeseed is more sensitive to water stress at the flowering and seed-forming stages (Elferjani and Soolanayakanahally, 2018). Abiotic stress such as drought affects the accumulation of phenolic compounds in plants (Gómez-Caravaca et al., 2012). Drought stress (DS) triggers the accumulation of reactive oxygen species (ROS) that can initiate lipid peroxidation, chlorophyll and betalain bleaching, and protein oxidation (Li et al., 2022). In response to the adverse effects of environmental conditions, plants produce a series of enzymatic and nonenzymatic defense systems for scavenging and detoxifying ROS, resulting in an enhanced antioxidant defense capacity (Huang et al., 2019; Farooq et al., 2021). Nonenzymatic antioxidants comprising of ascorbic acid, carotenoids, phenolic, and flavonoids have a protective role against the production of ROS in plants (Zhang et al., 2019).

Genetic analysis of drought-adaptive traits helps to identify the most important genes involved in networks and pathways for use in marker-assisted selection (MAS) programs and cultivar development under variable environmental conditions. Drought tolerance is a complex trait regulated by several minor and/or major effects quantitative trait loci (QTL) (Maazou et al., 2016). In rapeseed, a genome-wide association study (GWAS) has been used for genetic analysis of a range of traits such as plant height and primary branch number (Li et al., 2016), time of flowering (Xu et al., 2016), seed coat color (Wang et al., 2017), blackleg disease (Fikere et al., 2020), and oil content (Pal et al., 2021). However, only a few studies have analyzed the use of GWAS to dissect the genetics of drought tolerance-related traits in rapeseed, many of which have been based on low-marker density maps that may not be able to capture the diversity of drought tolerance mechanisms. Zhang et al. (2015) analyzed 66 rapeseed accessions under drought conditions and identified 16 single-nucleotide polymorphisms (SNPs) significantly associated with water stress-related responses. Recently, Shahzad et al. (2021) identified 139 SNPs associated with the water loss ratio using GWAS in 264 rapeseed accessions at the full-bloom stage, of which four were related to putative candidate genes (CGs) involved in drought tolerance in rapeseed.

The genetic basis of the phenolic compounds in response to DS and associated CGs has not yet been characterized in rapeseed germplasm under DS conditions. Rapeseed flowers have nutritional and industrial benefits, but this organ and the flowering stage are vulnerable to drought and, therefore, to seed production. In this study, our aim was to first assess the relation of flower polyphenols and drought tolerance and then identify linked markers/genes to find drought tolerance by selecting high-polyphenol varieties in rapeseed. First, we investigated the effects of irrigation regimes on phytochemical traits and polyphenols and induction of gene regulatory elements. Then, a GWAS analysis was conducted in the well-watered (WW) and DS conditions to assess the genomic architecture of the phytochemical traits and polyphenols. The RNA sequencing (RNA-seq) trial was performed in order to validate genes associated with the linked SNPs and identify key CGs involved in the regulatory pathway of phenolics. Differentially expressed genes (DEGs) between contrasting phenotypes (high- vs. low-phenolic content varieties) were identified and compared with genes underlying the genomic region (GR) of SNPs identified for the tested traits. Our intent was to identify key regulatory genes in phenolic biosynthetic pathways and provide better insight into the genetic basis underlying the establishment of high-phenolic compound varieties showing drought tolerance in rapeseed.

## Materials and methods

### Field experimental conditions

The plant materials consisted of 119 rapeseed genotypes including inbred lines, hybrids, and commercial cultivars (**Supplementary Table S1**). The seeds of the genotypes were sown in 11 × 11 lattice experimental design with three replications. Experimental plots were composed of four 1-m-long rows, each with 25 plants in 4-cm spaces. The experiments were performed under two irrigation treatments, one a WW condition and the other a DS treatment at the Research Farm of Plant Production and Genetics, Shiraz, Iran. The seeds were sown on 17 September 2020 in a silty loam soil. Information on macronutrient and micronutrient elements of soil is shown in **Supplementary Table S2**. Fertilizers were applied at rates of 250 kg N ha^−1^, 100 kg P_2_O_5_ ha^−1^, and 100 kg K_2_SO_4_ ha^−1^. Nitrogen fertilizer was added to the soil prior to sowing, at the time of rosette leaf stage, and at the beginning of flowering. In the WW condition, irrigation was carried out until seed physiological maturity at the time when no further increase in seed weight occurred. In the DS treatment, irrigation was carried out until 50% pod development stage. The weather data during the growing season show that rainfall did not occur during implementation of the DS period (**Supplementary Figure S1**).

### Sampling and preparation of the extracts

In a study, the results showed that the flower accumulates the highest polyphenols and antioxidant activity compared with leaves and seeds (Salami et al., 2023), which supported the results of the study by Khan et al. (2021). Four weeks after ceasing irrigation in the drought treatment and at the end of flowering, 10 flower samples were cut from five main plants in both irrigation regimes, snap frozen in liquid nitrogen, and stored at -80°C. The flower samples were homogenized with 80% methanol of 1:2 (w/v) at 4°C. The homogenization was sonicated for 10 min and mixed at 15 rpm on a tube rotator. Homogenized samples were immediately placed on a stirrer at room temperature for 2 h and centrifuged at 10,000 g for 20 min. Supernatants in triplicate were collected for further analyses (Samec et al., 2011).

### Biochemical assays

Total phenolic content (TPC) was quantified as described by Sarker and Oba (2018). Fifty microliters of the extract was mixed with 1 mL of Folin–Ciocalteu phenol reagent. Then, 1 mL of 10% sodium carbonate solution was added to the mixture and shaken thoroughly. The mixture was left for 30 min, and the blue color formed was measured at 760 nm wavelength using a spectrophotometer. A regression equation for the calibration curve (y = 0.0095 x - 0.113, *R^2 ^* = 1) of gallic acid was used, and the data were presented as μg gallic acid equivalent (GAE) g^−1^ dry weight (DW).

Total flavonoid content (TFDC) was determined with an aluminum chloride reagent followed by a colorimetric method (Zou et al., 2004). A total of 0.5 mL of each extract was mixed with 150 µL of 10% aluminum chloride and 2 mL of 1 M sodium hydroxide. The mixtures were incubated for 40 min at room temperature in the dark. The absorbance at 510 nm was measured using a spectrophotometer (Model M550 UV/VIS Spectrophotometer CamSpec). A calibration curve was drawn using a quercetin standard dilution series, and the data were shown as mg quercetin equivalents (QUE) g^−1^ DW.

The total flavonol content (TFLC) was determined using the *p*-dimethylaminocinnamaldehyde (DMACA) method (Kusznierewicz et al., 2008). Four hundred microliters of extract was mixed with 600 μL of DMACA solution (0.1% in 1 M HCl in MeOH). The mixture was vortexed, and the absorbance was measured at 640 nm after incubation at room temperature for 10 min. Catechin (CE) was used as a standard, and the TFLC data were expressed as μg catechin equivalents (CE) g^−1^ DW.

### Determination of total anthocyanin content and ascorbic acid content

The total anthocyanin content (TAC) was determined using a pH difference method (Egbuna et al., 2018). Two milliliters of the extracts were added to 2 mL of hydrochloric acid (2 N) and 2 mL of ammonia (2N). The extracts were diluted with 0.025 M hydrochloric acid–potassium chloride buffer (pH = 1) and 0.4 M sodium acetate buffer (pH = 4.5). Each sample was diluted with the buffers, and the absorbance of the mixture was measured at 700 nm using a UV-Vis spectrophotometer (UV1601; Shimadzu, Kyoto, Japan). The total anthocyanin content was expressed as cyanidin-3-glucoside equivalents as follows (Zou et al., 2011; Maran et al., 2015):


(1) Anthocyanin pigment (mg/L) = A × MW × DF × V × 1,000/a × l × m

where *A* is the absorbance, *MW* is the molecular weight of cyanidin-3-glucoside (449.2 g/mol), *DF* is the dilution factor, *V* is the solvent volume (mL), a is the molar absorptivity (26,900 L mol^-1^ cm^-1^), *l* is the cell path length (1 cm), and *m* is the weight of the sample.

The ascorbic acid content (AAC) of extracts was determined by the 2,6-dichlorophenol-indophenol (DCPIP) method as described by Rao and Deshpande (2006). The freeze-dried samples (0.5 g) were extracted using 20 mL (w/v) metaphosphoric acid (3%) followed by shaking at 300 rpm for 30 min. The extract was centrifuged at 4,000 rpm for 10 min. Here, 1 mL of extract was added to 3 mL of 0.2 mM DCPIP, and the spectrophotometer reading was performed at 515 nm immediately after mixing for 15 s. The results were expressed in mg ascorbic acid per 100 g dry weight (mg/100 g DW).

### High-performance liquid chromatography and phenolic compounds

Chromatographic analysis was performed on a Varian ProStar 210 system equipped with a vacuum degasser, binary pump, thermostatted column compartment, and diode array detector (DAD) connected to ChemStation software ver. 4.03.016 (Lopez et al., 2011). The compounds were separated using a reverse-phase Pursuit XRs C18 (250 mm × 4.6 mm, 5 µm) column and a Pursuit XRs C18 (10 mm × 4.6 mm, 5 µm) guard column (Varian, Barcelona) at 27°C and based on a gradient system with two mobile phases. Eluents A and B were water with 0.1% formic acid and methanol, respectively. The flow rate was 1.0 mL/min, and the injection volume was 60 µL of crude extracts. The elution conditions used were as follows: 0.5 min, 20% B isocratic; 5-30 min, linear gradient from 20% to 60% B; 30-35 min, 60% B isocratic; 30–40 min, linear gradient from 60% to 20% B, and finally wash and recondition the column. Simultaneous monitoring for quantification of phenolics was performed at 270 nm (gallic acid, protocatechuic acid, catechin, vanillic acid, epicatechin, and syringic acid), 324 nm (chlorogenic acid, gentisic acid, caffeic acid, coumaric acid, and ferulic acid), and 373 nm (rutin, myricetin, and quercetin). The stock standard solution diluted with methanol was used to obtain an appropriate concentration 1.0–100 µg/mL range for establishing the calibration curves. For quantitative analysis, four different concentrations of 14 analytes were injected in triplicate.

### Evaluation of the antioxidant activity by DPPH method

The free radical scavenging activity (RSA) of the extracts was evaluated with 1,1-diphenyl-2-picryl-hydrazil (DPPH) following Molyneux (2004) with some modifications. Briefly, 193 μL methanolic solution of free radical DPPH (60 μM) was added to 7 μL of the sample extracts. After incubation in the dark at room temperature for 30 min, the absorbance of the mixture at 515 nm against methanol was measured as a blank using a spectrophotometer-microplate reader (Epoch, BioTek, Instruments, Inc.). The control solution was 200 μL methanolic solution of free radical DPPH (60 μM). The RSA of the extracts was expressed as a percentage of the reduced DPPH as follows:


(2) $$\text{RSA }\left(\%\right)\text{ = }\left[\left({\text{A}}_{0}{\text{- A}}_{1}\right){\text{/A}}_{0}\right]\times 100$$

where A_0_ = absorbance of the control and A_1_ = absorbance of the test extracts.

### Statistical analyses of phytochemical traits

Descriptive statistics including means and standard deviations were obtained using the Statistical Analysis System (SAS, Version 9.3, SAS Institute Inc., Cary, NC, USA). Analysis of variance (ANOVA) was performed based on a generalized linear model (GLM) with genotype as fixed and replication as random effects. The principal component analysis (PCA) and hierarchical cluster analysis (HCA) were performed to visualize the interrelationships between phenolic compounds and similarities of genotypes under two experimental conditions in R version 3.4.0 (http://www.r-project.org/). Stepwise regression analysis was used to determine how much antioxidant activity variability could be explained by the independent variables (TPC, TFDC, TFLC, AAC, TAC, and phenolic compounds).

### Genotyping and filtering of single-nucleotide polymorphisms

Genomic DNA of 119 rapeseed accessions was extracted from young leaf tissue of greenhouse-grown plants using a cetyltrimethylammonium bromide (CTAB)-based method (Murray and Thompson, 1980). The genomic DNA was quantified using a Nanodrop ND1000 Spectrophotometer (Nanodrop Technologies, Inc., Wilmington, DE, USA). The DNA samples were used for genotyping with the Brassica 60 K Infinium array as described in the manufacturer’s protocol (Illumina Inc., San Diego, CA, USA). The SNPs were filtered for site coverage (90%), minimum minor allele frequency (MAF) of 0.05 with only biallelic markers, and low rates of missing data (≤10%) using the TASSEL ver. 5 software (Bradbury et al., 2007). The SNPs with low quality and high rates of missing data were removed using a protocol described in Iquira et al. (2015) and Kujur et al. (2015). Analyses of polymorphic information content (PIC) were performed using the software PowerMarker version 3.25 (Liu and Muse, 2005).

### Population structure and linkage disequilibrium analysis

Bayesian cluster analysis was used for analysis of the population structure and the fraction of the genetic ancestry of a single accession that belongs to a population using STRUCTURE software (Sehgal et al., 2015). The most appropriate subpopulation number (K) was obtained using the Ln probability of the data [Ln P(D)] and the delta K values were calculated according to Evanno et al. (2005). The maximum of the 1K peak was considered as the true cluster count. The linkage disequilibrium (LD) between pairs of polymorphic loci was analyzed using the TASSEL software package (Bradbury et al., 2007; http://www.maizegenetics.net/). LD was estimated using the squared allele frequency correlations (*r^2^*), which is a measure of the correlation between a pair of variables (Hill and Robertson, 1968). To investigate chromosome-wide and genome-specific patterns of LD, the software package TASSEL 4.0 was used to estimate LD (*r^2^*) on each chromosome and across the A- and C-sub genomes, respectively (Bradbury et al., 2007). Pairwise LD explained by *r^2^* was determined for 29,310 high-quality SNPs with MAF ≥0.05. Locally estimated scatterplot smoothing (LOESS, stands for locally weighted scatterplot smoothing) curves were then fitted into the scatter plot using R software. Specifically, the 95th percentile of the distributions of *r^2^* of the selected loci was estimated as the threshold *r^2^* with the assumption that LD was attributable to linkage (Breseghello and Sorrells, 2006).

### Genome-wide association study analysis

Marker–trait association (MTA) analysis was performed using the TASSEL program based on the mixed linear model (MLM) approach with the principal components (PCs) and kinship (K) matrices as cofactor (Bradbury et al., 2007). The MLM-based analysis requires the data for population structure (Q), PCs, K, and a total of 29K high-quality SNPs in 119 rapeseed genotypes (Yu et al., 2006). The K-Matrix was generated with the default parameters by choosing the Centered_IBS method to get a better estimate of the additive genetic variance. Analysis of MTAs was also performed with the general linear model (GLM) and GLM+Q and GLM+PCA models (Yu et al., 2006). The program’s default settings were used to filter marker data for minimum genotype number and MAF. The phenotypic variation (*R^2^%*) explained was also estimated for each individual marker. Quantile-quantile (QQ) plots were shown with -log_10_(*P*) of each SNP and expected *P*-value using the R package qqman (https://cran.r-project.org/web/packages/qqman/index.html; Turner, 2014). The results of the MLM analysis were used to obtain the Manhattan plots in TASSEL.

### Identification of candidate genes

To determine CGs related to phenolic compounds under WW and drought-stressed conditions, the position of flanking SNPs obtained from the “Darmor-bzh” reference genome (http://www.genoscope.cns.fr/brassicanapus/data/) was used to search in the rapeseed genome by EnsemblPlants (https://plants.ensembl.org/). Consequently, all of the genes underlying the GR of each SNP were functionally annotated by EnsemblPlants (https://plants.ensembl.org/) and online resources (https://genome.ucsc.edu/ and https://www.ncbi.nlm.nih.gov/). The CGs were identified based on their putative function in rapeseed or closely related species.

### Allele effect and haplotype analysis

Allele effects for the linked significant SNPs were analyzed as previously described by Alemu et al. (2021). Genotypes were divided into two different groups according to their specific SNP alleles, and the means were compared using Tukey’s Honest Significant Difference (HSD) test. Exploring and harnessing haplotype diversity help in the detection of CGs for improvement of target traits in crops (Qian et al., 2017). A haplotype association test was performed to investigate the combined effect of the linked significant SNPs. The SNPs in the same haploblock and the LD of significant SNPs were determined using Haploview 4.2 (Barrett et al., 2005). Standardized disequilibrium coefficient (D’) was used to evaluate the LD between markers and generate the LD heatmap. Haploid blocks were detected based on LD using the confidence interval (CI) method in Haploview 4.2 (Gabriel et al., 2002).

### RNA-seq and biological validation of the identified candidate genes

RNA-seq analysis in two contrasting lines, a top high (G67) and a low-phenolics (G59) cultivar, was used to validate the CGs associated with the SNPs of phenolic compounds. The plants were grown in pots in a greenhouse (26°C–28°C) under short-day (12 h of light and 12 h of dark) conditions until the initial flower stage. When the flower was visible, half of the plants were exposed to DS conditions. During irrigation, drought-treated flower pots maintain a soil relative water content of 10% (irrigated with PEG6000 at a concentration of 20%), while adequately watered flower pots maintain a soil relative water content of 30% (irrigated with sterile water of equal volume) with repeating the experiment three times. The relative water content of soil was calculated according to the following formula (Zhang et al., 2023):


(3) Soil moisture content (%) = (W2-W3)/(W3-W1) × 100%

where W1 is the weight of the container, W2 is the weight of the container with wet soil, and W3 is the weight of the container with dry soil.

Fifteen days after flowering, the top flowers were harvested from plants in each replicate group and each condition (WW/control or DS). All harvested flowers were immediately frozen using liquid nitrogen and were transferred to a deep freezer (−80°C) for storage.

Total RNA was isolated from each sample using a Plant RNA Mini Kit (Tiangen, Inc., China) according to the manufacturer’s protocol. Four cDNA libraries were constructed, and RNA-seq was performed on a DNBSEQ-G400 platform. Low-quality reads were filtered out using NGS QC toolkit v2.2.3 (https://omictools.com/ngs-qc-toolkit-tool) (Patel and Jain, 2012), and clean reads were mapped in the reference genome of “Darmor-bzh” (http://www.genoscope.cns.fr/brassicanapus/). The obtained genes from the previous step were quantitatively analyzed using Cluffquant and Cluffnorm of Cufflinks 2.0.0 (http://cole-trapnell-lab.github.io/cufflinks/releases/v2.0.0/). Gene expression levels were estimated by the FPKM (fragments per kilobase of exon per million mapped fragments) and an all by all differential expression analysis by combining replications. DEGs in the specific paired sample comparisons were identified with the log2 fold change between the two samples and the *P*-value given to the comparison along with the FPKMs for each of the two samples in the comparison.

## Results

### Phenotypic variation for phytochemicals in rapeseed population

**Figure 1:**
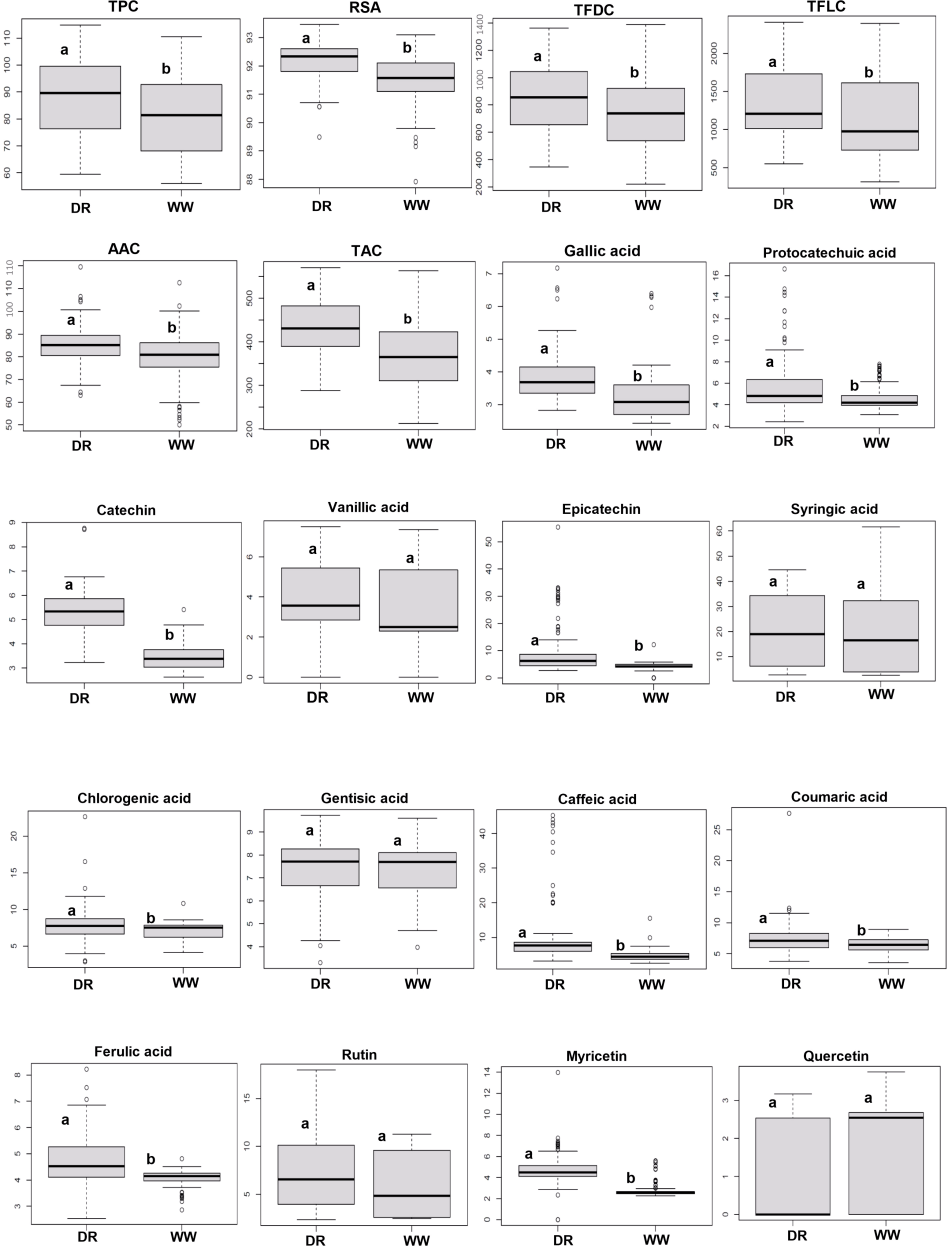
Boxplot of trait variations for rapeseed (*Brassica napus*) genotypes in the well-watered condition (WW) and drought stress condition (DR). Different letters on the boxes indicate a significant difference at *P*< 0.05 by Tukey’s HSD. The horizontal line within the box represents the median. The lower and upper limit of the box, lower and upper whisker represents Q1 (first quartile/25th percentile), Q3 (third quartile/75th percentile), (Q1−1.5IQR) and (Q3 + 1.5IQR), respectively. IQR, interquartile range.

The ANOVA revealed a significant effect of drought on TPC, TFDC, TFLC, AAC, TAC, and RSA and variations in 119 genotypes (**Supplementary Table S3**). The interaction of treatment (WW and DS conditions) × genotype was significant for TPC, TFDC, and TFLC. The means of the traits varied between WW and drought-stressed genotypes as shown in **Table 1**. The frequency distribution of genotypes and box plots for phytochemicals across two moisture conditions are presented in **Supplementary Figure S2** and **Figure 1**. The largest and smallest variations belonged to the means for TFLC and RSA, respectively (**Table 1**). The average of TPC, TFDC, TFLC, AAC, TAC, and RSA under DS was 87.09 μg GAE g^−1^ DW, 851.97 mg GAE g^−1^ DW, 1,370.45 μg CE g^−1^ DW, 85.33 mg/100 g DW, 427.67 mg/L, and 92.18%, respectively, which was higher than those in WW conditions. The absolute kurtosis and skewness of most of the phytochemicals were<1, reflecting the normal distribution of the traits (**Table 1**).

**Table 1:** Descriptive statistics for the phytochemical traits, phenolic compounds, and antioxidant activity of 119 rapeseed (*Brassica napus*) genotypes grown at two irrigation treatments.

Trait	Unit	Treatment	Range	Mean ± SD^a^	Skew^b^	Kurt^c^
TPC	μg GAE g^−1^ DW	WW	55.94 - 110.63	81.36 ± 1.23	-0.12	-1.08
		DS	59.36 - 114.89	87.09 ± 2.30	-0.2	-0.95
TFDC	mg GAE g^−1^ DW	WW	219.86 - 1388.43	743.15 ± 9.52	0.35	-0.41
		DS	354.57 - 1362.72	851.97 ± 8.48	0.15	-0.81
TFLC	μg CE g^−1^ DW	WW	314.5 - 2392	1214.62 ± 0.78	0.65	-0.81
		DS	553.3 - 2408.3	1370.45 ± 0.59	0.64	-0.77
RSA	%	WW	87.92 - 93.09	91.48 ± 1.01	-1.1	2.14
		DS	89.48 - 93.45	92.18 ± 1.20	-0.84	1.24
AAC	mg/100 g DW	WW	50.07 - 112.565	79.85 ± 1.87	-0.54	1.44
		DS	63.08 - 119.55	85.33 ± 1.07	0.44	2.1
TAC	mg/L	WW	212.58 - 563.09	370.75 ± 0.72	-0.02	-0.65
		DS	288.14 - 570.37	427.67 ± 0.77	-0.16	-0.56
Gallic acid	mg/100 g DW	WW	2.43 - 6.39	3.21 ± 1.05	2.48	1.54
		DS	2.83 - 7.17	3.83 ± 2.64	0.84	1.23
Protocatechuic acid	mg/100 g DW	WW	3.09 - 7.76	4.61 ± 0.46	0.46	1.04
		DS	2.41 - 16.62	5.7 ± 0.75	1.09	1.32
Catechin	mg/100 g DW	WW	2.63 - 5.41	3.42 ± 1.50	0.8	0.77
		DS	3.23 - 8.76	5.34 ± 1.31	0.7	1.11
Vanillic acid	mg/100 g DW	WW	nd - 7.36	4.11 ± 1.26	-0.01	-1.74
		DS	nd - 7.50	4 ± 0.36	-0.17	-0.23
Epicatechin	mg/100 g DW	WW	2.59 - 12.22	4.48 ± 1.35	2.45	1.32
		DS	2.73 - 55.38	9.19 ± 1.90	1.21	1.11
Syringic acid	mg/100 g DW	WW	2.60 - 61.67	17.17 ± 1.14	0.42	-1.13
		DS	2.74 - 44.52	19.63 ± 2.61	0.27	-1.64
Chlorogenic acid	mg/100 g DW	WW	4.13 - 10.83	7.07 ± 1.14	-0.52	0.35
		DS	2.87 - 22.65	7.84 ± 1.31	1.13	0.98
Gentisic acid	mg/100 g DW	WW	3.97 - 9.60	7.32 ± 1.99	-0.89	0.08
		DS	3.3 - 9.73	7.36 ± 0.78	-0.89	0.35
Caffeic acid	mg/100 g DW	WW	2.52 - 15.52	4.54 ± 1.04	1.64	1.36
		DS	3.11 - 45.21	9.8 ± 1.72	1.06	1.12
Coumaric acid	mg/100 g DW	WW	3.53 - 8.88	6.35 ± 0.27	-0.25	-0.36
		DS	3.77 - 27.64	7.41 ± 0.67	1.22	0.78
Ferulic acid	mg/100 g DW	WW	2.86 - 4.81	4.07 ± 3.37	-1.32	1.73
		DS	2.53 - 8.22	4.71 ± 3.14	0.89	1.88
Rutin	mg/100 g DW	WW	2.44 - 11.27	5.54 ± 0.68	0.57	-1.4
		DS	2.34 - 17.98	7.21 ± 1.52	0.34	-0.64
Myricetin	mg/100 g DW	WW	2.25 - 5.59	2.74 ± 0.15	1.43	1.24
		DS	nd - 13.97	4.72 ± 0.23	1.24	1.35
Quercetin	mg/100 g DW	WW	nd - 3.75	2.68 ± 0.99	1.76	1.32
		DS	nd - 3.17	1.15 ± 0.36	0.29	-1.34

nd, not detected. **^a^** Standard deviation. **^b^** Skewness, which reflects the asymmetry of the probability distribution of a real-valued random variable about its mean. **^c^** Kurtosis, which reflects the “tailedness” of the probability distribution of a real-valued random variable. TPC, TFDC, TFLC, RSA, AAC, and TAC were the abbreviations of total phenolic content, total flavonoid content, total flavanol content, radical scavenging activity, ascorbic acid content, and total anthocyanin content. WW and DS were the codes of well-watered and drought stress conditions.

### Profiling of phenolic compounds under well-watered and drought stress conditions

**Figure 2:**
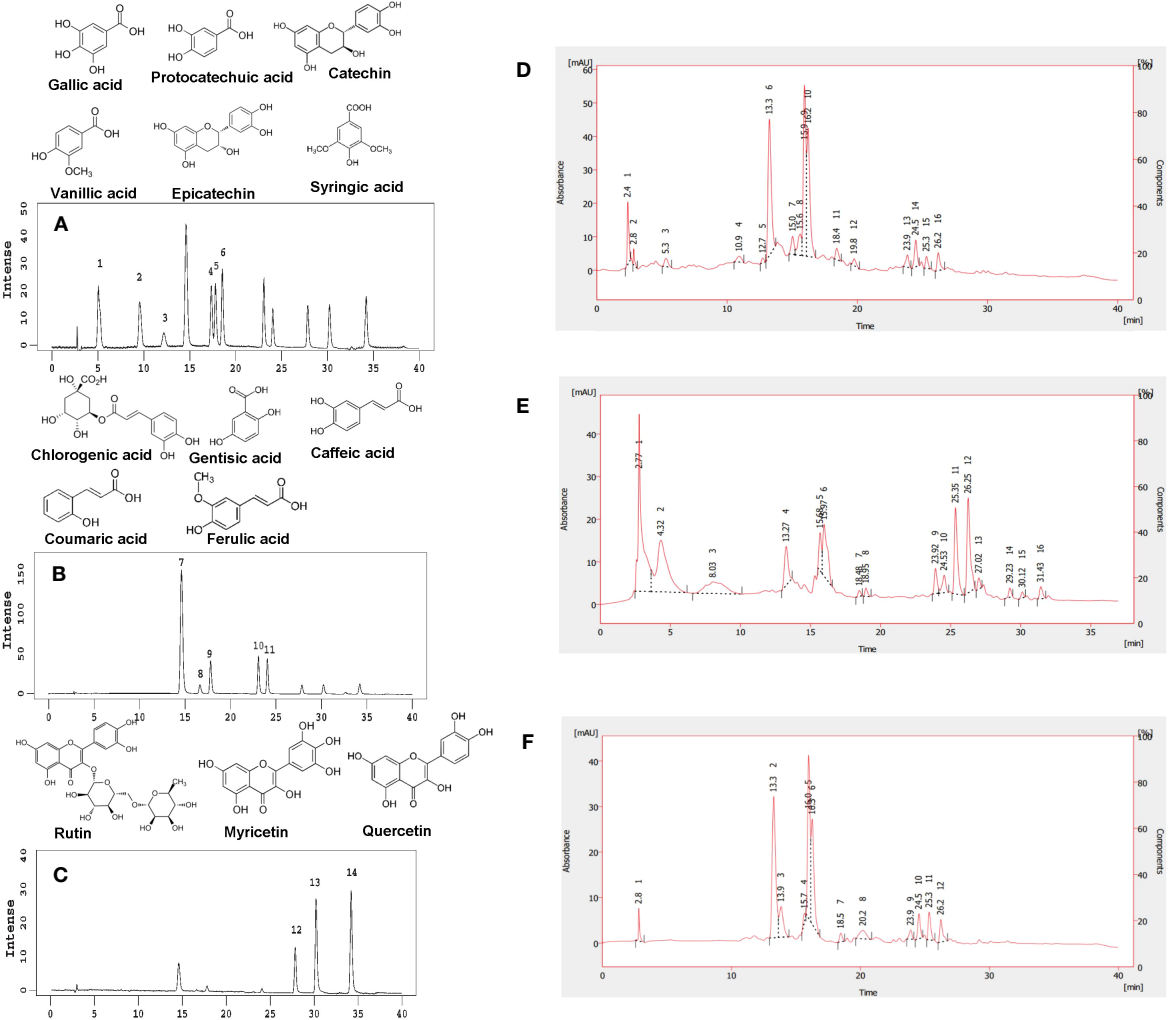
Chemical structures of 14 phenolic compounds and HPLC chromatograms of standard and sample polyphenols. **(A)** Chemical structures of gallic acid, protocatechuic acid, catechin, vanillic acid, epicatechin, and syringic acid and HPLC chromatograms of phenolic acids and flavonoids at 270 nm: peak 1 = gallic acid, peak 2 = protocatechuic acid, peak 3 = catechin, peak 4 = vanillic acid, peak 5 = epicatechin, and peak 6 = syringic acid. **(B)** Chemical structures of chlorogenic acid, gentisic acid, caffeic acid, coumaric acid, and ferulic acid and HPLC chromatograms of phenolic acids; 324 nm: peak 7 = chlorogenic acid, peak 8 = gentisic acid, peak 9 = caffeic acid, peak 10 = coumaric acid, and peak 11 = ferulic acid. **(C)** Chemical structures of rutin, myricetin, and quercetin and HPLC chromatograms of flavonoids; 373 nm: peak 12 = rutin, peak 13 = myricetin, and peak 14 = quercetin. HPLC chromatograms of sample polyphenols at **(D)** 270 nm, **(E)** 324 nm, and **(F)** 373 nm.

Interactions were significant for phenolic compounds except chlorogenic and gentisic acids (**Supplementary Table S3**). In the analysis of high-performance liquid chromatography (HPLC), 14 phenolic compounds from phenolic acid and flavonoid groups were identified (**Figures 2A–F**). Gallic, protocatechuic, vanillic, syringic, chlorogenic, gentisic, caffeic, coumaric, and ferulic acids were the major phenolic acids identified. The epicatechin, syringic, caffeic, coumaric, chlorogenic, and protocatechuic acids were more frequent than other compounds in the tested accessions (**Table 1**; **Figures 2D–F**).

**Figure 3:**
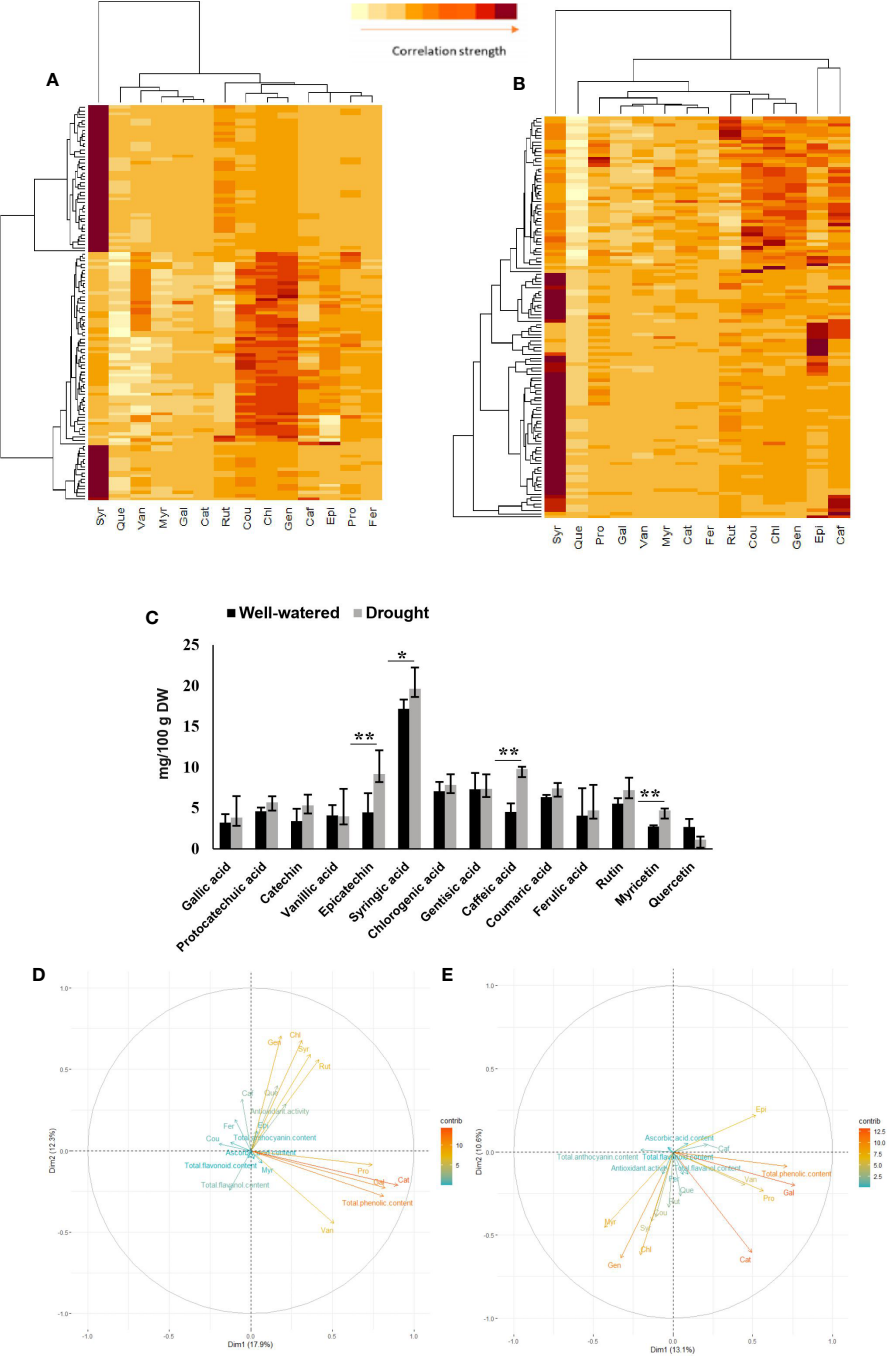
Relation of antioxidant activity and phenolics in rapeseed (*Brassica napus*) in the well-watered and drought stress conditions. Heatmap visualization of relative differences of phenolic compounds accumulation among rapeseed accessions in the well-watered condition **(A)** and drought stress condition **(B)**. Each accession is visualized in a single row and each phenolic compounds is represented by a single column. The dark color indicates high abundance, and light indicates low relative phenolic compounds contents. **(C)** Average amount of 14 phenolic acids and flavonoids across the rapeseed diversity panel are compared between two conditions (well-watered and drought stress) with Student’s t-tests (*P <0.05 and ** P < 0.01). Error bars = SE. **(D)** Principal component analysis (PCA) plot of variables (phytochemical traits, antioxidant activity and phenolic compounds) in the rapeseed accessions in the well-watered condition. **(E)** Principal component analysis (PCA) plot of variables (phytochemical traits, antioxidant activity and phenolic compounds) in the rapeseed accessions under drought stress condition.

Results of HCA are shown in **Figures 3A, B**. In the WW condition, quercetin, vanillic acid, myricetin, gallic acid, and catechin clustered as one group and rutin and coumaric acid in another group with chlorogenic and gentisic acids (**Figure 3A**). The heat map of genotypes based on their phenolics in the DS condition showed a different spatial distribution of phenolics from WW, where protocatechuic acid, gallic acid, vanillic acid, myricetin, catechin, ferulic acid, rutin, coumaric acid, chlorogenic acid, and gentisic acid were closely associated (**Figure 3B**).

### Phytochemicals and phenolic compounds increased under drought conditions

Under DS, TPC, TFDC, TFLC, AAC, and TAC were increased by 7.04%, 14.64%, 12.82%, 6.86%, and 15.35%, respectively (**Table 1**; **Figure 3C**). DS significantly increased the accumulation of phenolic acids and flavonoids such as caffeic acid (115.85%), epicatechin (105.13%), myricetin (72.26%), and syringic acid (14.32%).

### Relation of antioxidant activity and phenolics

The interrelationship of the antioxidant activity and phenolics was assessed by both the PCA and stepwise regression. The PCA was performed to identify the relationship between the pattern of phytochemicals and phenolics and the antioxidant activities. Theoretically, 17 PCs can be derived, but the PCs with higher contributions to total variation were selected for further analysis. In WW, seven PCs with eigenvalues greater than 1 explained 70.59% of the variability in the dataset (**Supplementary Table S4**). The first two PCs (PC1 and PC2) that explained 30.3% of the total variation of traits were used in biplot analysis (**Figure 3D**). PC1 represented the variation in gallic acid, catechin, and TPC, while PC2 explained the variation of syringic acid (46%) and rutin (43.4%). Rutin, syringic acid, chlorogenic acid, epicatechin, quercetin, and gentisic acid showed a stronger association with antioxidants, while caffeic, ferulic, and coumaric acids showed a negative correlation with antioxidant activity (**Figure 3D**).

Under DS, eight PCs with eigenvalues greater than 1 explained 66.13% of the variability in the dataset (**Supplementary Table S5**). The first two PCs explaining 23.7% of the total variation of the traits were used for the analysis of the relation of traits in a biplot (**Figure 3E**). Catechin was the most important contributor to PC1. For PC2, vanillic acid, with a positive coefficient, was the most representative variable. Gentisic acid, syringic acid, chlorogenic acid, coumaric acid, myricetin, and rutin showed a strong association with antioxidant activity, while quercetin, catechin, vanillic acid, protocatechuic acid, and gallic acid had negative correlations with antioxidant activity (**Figure 3E**). Results of the stepwise regression analysis showed that chlorogenic acid in the WW condition and gentisic acid, coumaric acid, and myricetin in the DS treatment had higher contributions to RSA (**Supplementary Table S6**).

### Distribution of SNPs, population structure, and LD analysis

**Figure 4:**
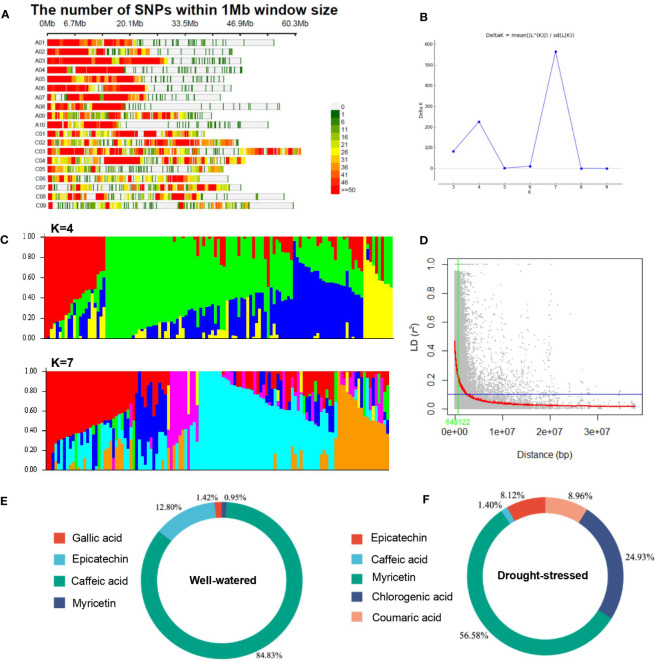
Population structure and linkage disequilibrium analysis in the rapeseed (*Brassica napus*) genotypes. **(A)** SNP density plot chromosome wise representing number of 29,310 SNPs within 1 Mb window size. The horizontal axis shows the chromosome length (Mb); the different color depicts SNP density. **(B, C)** Delta-K values and population structure analysis and of 119 rapeseed accessions. **(B)** Delta-K values for different numbers of populations assumed (K) in the STRUCTURE analysis. **(C)** Population structure analysis, K=4 and K=7. The distribution of the genotypes to different populations is indicated by color code. The number on the y-axis shows the subgroup membership, and the x-axis shows the different genotypes. **(D)** Decay of linkage disequilibrium (LD) (*r* ^2^) as a function of genetic distance (bp) between pairs of loci on all chromosomes in the rapeseed (*Brassica napus*) accessions (n=119). The blue lines indicate the 95th percentile of the distribution of unlinked *r* ^2^, which gives the critical value of *r* ^2^. The LOESS fitting curve (red line) illustrates the LD decay. Summary of significant SNPs detected under well-watered **(E)** and drought-stressed **(F)** conditions for phenolic compounds in rapeseed flowers.

Of the 52,157 SNPs, 22,847 SNPs with a MAF of<0.05 were removed and the remaining 29,310 SNPs were used to assess the population structure, LD, and association analysis. The filtered SNPs were distributed across the 19 chromosomes with 722 SNPs on chromosome C05 to 2,623 on C04 (**Figure 4A**).

The results of the population structure analysis are shown in **Figures 4B–D**. The clustering program estimated the membership probability (Q-matrix) of each rapeseed genotype to combine into a number of hypothetical subpopulations (K3–K10), and the statistic index Δk value was generated for subsequent runs. The most significant change in Δk was observed when K increased from 6 to 7. Overall iterations of the ΔK that had a much higher likelihood for K = 7 suggests the presence of seven main subpopulations and one mixed population (**Figure 4D**).

The critical *r^2^* value from which the genome-wide LD decayed was estimated at 0.1 (**Figure 4D**). This revealed large differences in LD decay between different chromosomes, with LD extending from 102 Kbps on chromosome A10 up to 954 Kbps on A08 (**Table 2**). The distribution of *r^2^* with respect to the physical distances over chromosomes is shown in **Figure 4D** and **Table 2**. The genome-wide average LD (*r^2^*) was 0.028. The LD values showed variations between chromosomes. The LD varied from 0.022 (chromosomes C03 and C08) to 0.088 (chromosome C05) (**Table 2**).

**Table 2:** Average distance of linkage disequilibrium (LD) decay (*r^2^* < 0.1) on subgenomes A and C, calculated using 29,311 unique genome-wide SNP markers with minor allele frequency (MAF) ≥0.05, in a collection of 119 rapeseed (*Brassica napus*) genotypes.

	Chromosome	*LD* decay (kb)	*LD* (*r^2^*)	SNPs with high *LD* (*r^2^* > 0.8) (%)
Subgenome A	A01	627.86	0.023	8.76
	A02	708.52	0.025	12.26
	A03	564.58	0.027	5.40
	A04	942.40	0.026	6.78
	A05	653.05	0.027	10.81
	A06	561.67	0.023	9.30
	A07	535.16	0.027	8.00
	A08	953.78	0.027	22.98
	A09	776.33	0.024	9.30
	A10	101.61	0.028	6.61
Subgenome C	C01	103.65	0.027	21.57
	C02	112.52	0.024	1.81
	C03	942.60	0.022	1.04
	C04	102.94	0.023	21.54
	C05	169.42	0.088	1.76
	C06	153.44	0.024	1.22
	C07	175.17	0.024	1.22
	C08	166.58	0.022	7.02
	C09	202.23	0.027	2.99

### Genetic basis of the phenolic acids under drought stress

**Figure 5:**
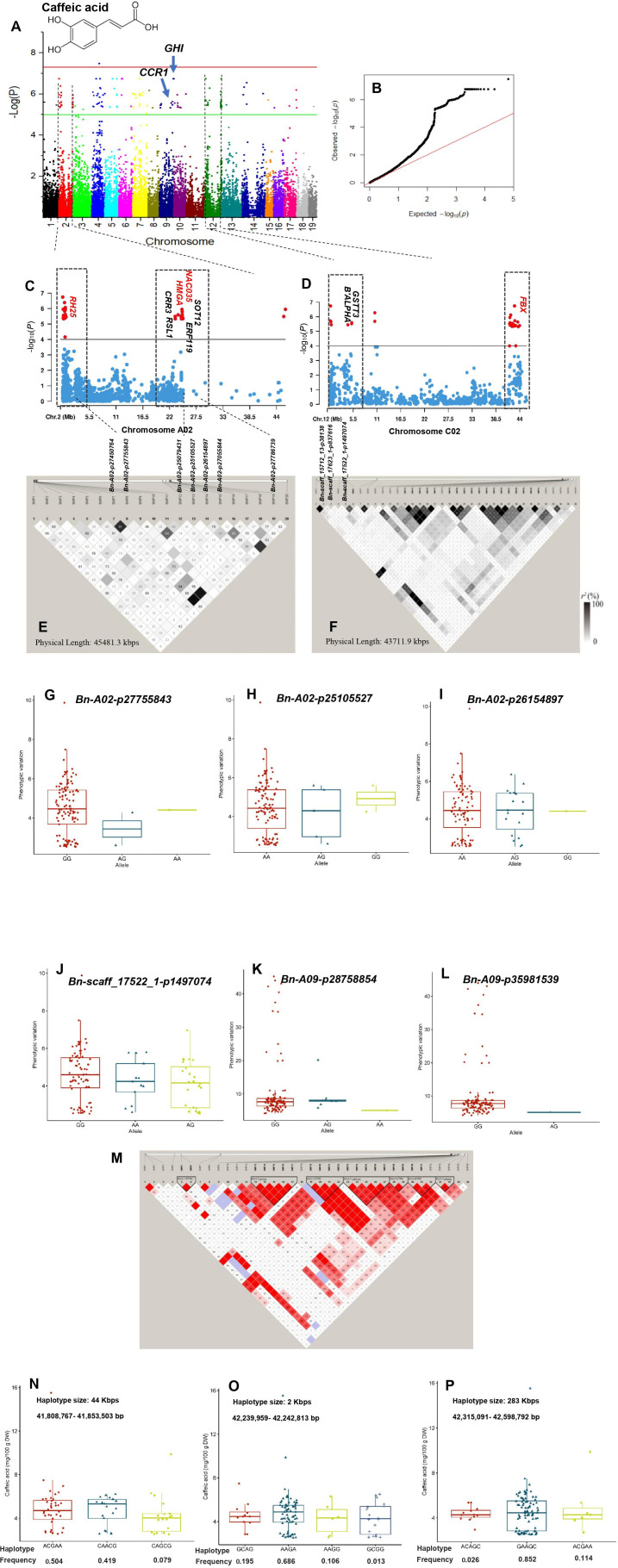
GWAS of caffeic acid using the 119 rapeseed (*Brassica napus*) genotypes. **(A)** Manhattan plot displaying the GWAS result of caffeic acid content in 19 chromosomes (1-10 stand for rapeseed chromosome of A01-A10 and 11-19 stand for rapeseed chromosome of C01-C09 at the horizontal axis) under well-watered condition. SNPs on different chromosomes are denoted by different colors. **(B)** The corresponding quantile-quantile (QQ) plot for GWAS of caffeic acid content under well-watered condition. **(C)** Locus zoom plot for caffeic acid associations in the chromosome A02. **(D)** Locus zoom plot for caffeic acid associations in the chromosome C02. **(E)** A representation of pairwise *r^2^* value (displayed as percentages) among polymorphic sites of chromosome 2 (A02) for caffeic acid. **(F)** A representation of pairwise *r^2^* value (displayed as percentages) among polymorphic sites of chromosome 12 (C02) for caffeic acid. Allele-effect analysis for six significant SNPs including Bn-A02-p27755843 **(G)**, Bn-A02-p25105527 **(H)**, Bn-A02-p26154897 **(I)**, Bn-scaff_17522_1-p1497074 **(J)**, Bn-A09-p28758854 **(K)**, and Bn-A09-p35981539 (L). The box plot depicts the number of the alleles for each of the six significant SNPs in 119 rapeseed accessions, and the contribution of these alleles to the phenotypic variation observed for caffeic acid level. **(M)** Haploview plot of the thirty-six significant SNPs on chromosome C02 for caffeic acid under well-watered condition. The coloring of the boxes is based on the scores of two estimates, logarithm of odds (LOD) and LD coefficient (Dʹ). Strong LD (LOD>2 and Dʹ=1) is indicated in red. A decrease in the intensity of red indicates lower LOD and Dʹ values. Regions with weak LD (LOD>2 and 0.21<Dʹ<1) are shown in blue. Regions with no LD (LOD<2 and Dʹ<1) are shown in white. The number within each box indicates the Dʹ statistic value multiplied by 100. Allelic effects of different haplotype blocks associated with caffeic acid in size 44 Kbps **(N)**, 2 Kbps **(O)**, and **(P)** 283 Kbps. Boxplots indicate the phenotype values corresponding to the different haplotype groups.

The MLM using both the PC and K matrix (MLM + K + PC) resulted in effectively controlling type I errors (false positives). Therefore, all subsequent GWAS analyses were performed using the MLM + K + PC model. A number of 613 significant SNPs (*P*< 1.0 × 10^–6^) associated with the phytochemicals and phenolics and RSA in the two irrigation conditions (**Supplementary Tables S7, S8**), 242 were associated with TPC, RSA, TFDC, TFLC, TAC, AAC, gallic acid, epicatechin, caffeic acid, and myricetin under WW and 371 were associated with RSA, TAC, and TFLC, epicatechin, caffeic acid, myricetin, chlorogenic acid, and coumaric acid under DS conditions (**Supplementary Tables S7, S8**; **Figures 4E, F**). The Manhattan and QQ plots for all traits are shown in **Figures 5** **,** **6** and **Supplementary Figures S3–S5**.

**Figure 6:**
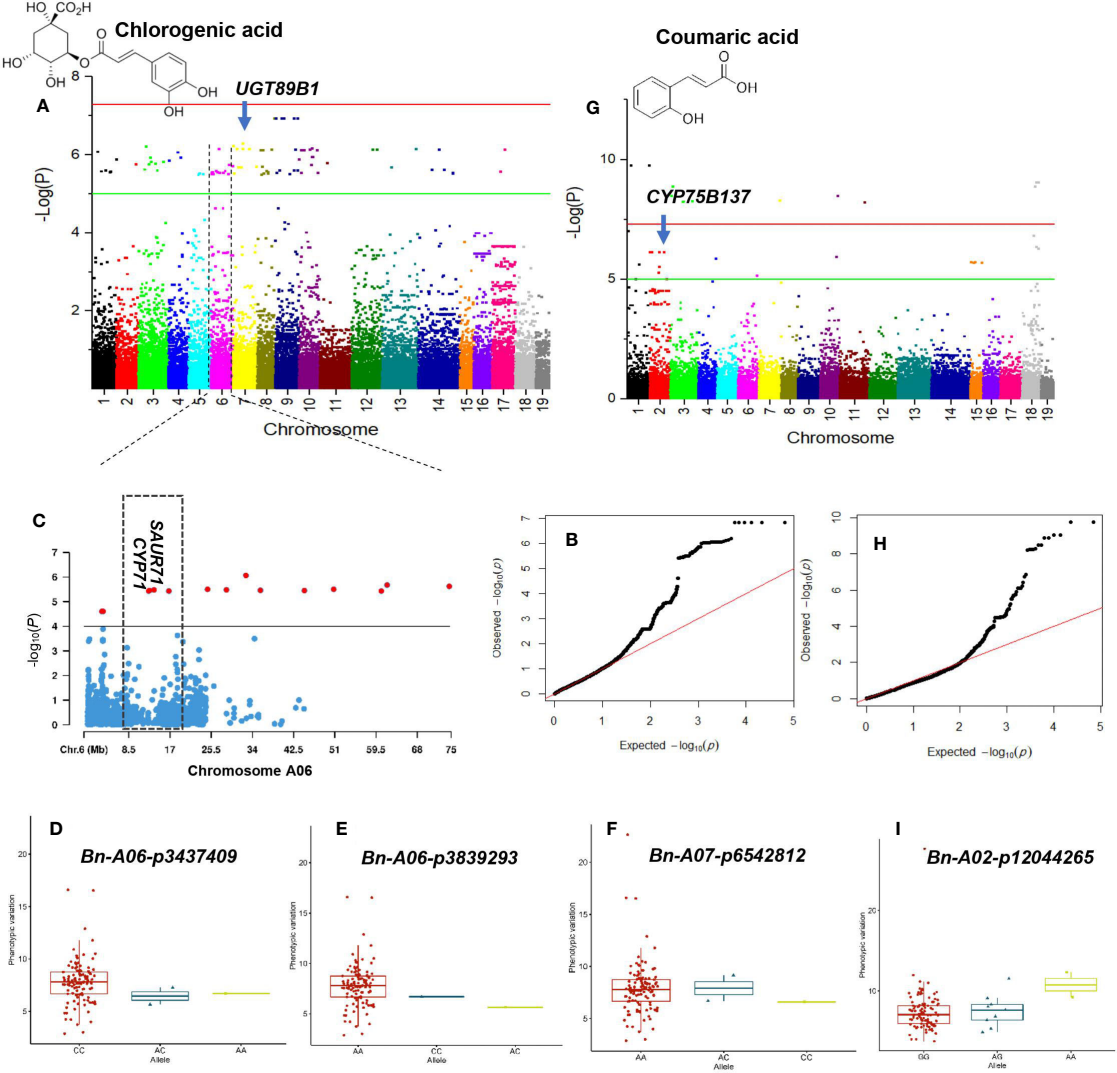
GWAS of chlorogenic acid and coumaric acid using the 119 rapeseed (*Brassica napus*) genotypes. **(A)** Manhattan plot displaying the GWAS result of chlorogenic acid content in 19 chromosomes (1-10 stand for rapeseed chromosome of A01-A10 and 11-19 stand for rapeseed chromosome of C01-C09 at the horizontal axis) under drought-stressed condition. SNPs on different chromosomes are denoted by different colors. **(B)** The corresponding quantile-quantile (QQ) plot for GWAS of chlorogenic acid content under drought stress condition. **(C)** Locus zoom plot for chlorogenic acid associations in the chromosome A06. Allele-effect analysis for significant SNPs including Bn-A06-p3437409 **(D)**, Bn-A06-p3839293 **(E)**, and Bn-A07-p6542812 **(F)**. The box plot depicts the number of the alleles for each of the four significant SNPs in 119 rapeseed accessions, and the contribution of these alleles to the phenotypic variation observed for chlorogenic acid level. **(G)** Manhattan plot displaying the GWAS result of coumaric acid content in 19 chromosomes under drought stress condition. **(H)** QQ-plot for GWAS of coumaric acid content under drought stress condition. Allele-effect analysis for significant SNP Bn-A02-p12044265 **(I)**. The box plot depicts the number of the alleles for the significant SNP in 119 rapeseed accessions, and the contribution of these alleles to the phenotypic variation observed for coumaric acid level.

GWAS analysis for TPC, TFDC, and TFLC showed that three SNPs, ChrA1:7615643, ChrA1:7669625, and ChrA1:7727474, with *P*-value = 7.48 × 10^–4^ were significantly associated with TPC, and 10 SNPs on chromosomes A05 (located on the 1838–17079 Kbps) and A06 were significantly associated with TFDC under WW (**Supplementary Table S7**). Ten SNPs distributed on chromosomes A04, A06, A10, and C04 and two SNPs (ChrA6:21114139 and ChrA10:12986214) on chromosomes A06 and A10 were significantly associated with TFLC under WW and DS conditions, respectively (**Supplementary Tables S7, S8**). A total of eight SNPs distributed on chromosomes A02, A05, A09, C04, and C07 were significantly associated with RSA under WW and DS conditions. The Percentage of Variance Explained (PVE) for these SNPs ranged from 13.59% for the SNPs ChrA9:3534018 and ChrC7:3534211 to 17.11% for the SNP ChrA5:21349945 under DS (**Supplementary Table S8**). GWAS analysis showed that three SNPs distributed on chromosomes A03, C03, and C08 and eight on chromosomes A03, C03, and C07 were significantly associated with the TAC under WW and DS, respectively (**Supplementary Tables S7, S8**). In the WW condition, two SNPs, ChrA3:9165370 (PVE = 14.24%) and ChrC8:34299868 (PVE = 16.34%), were significantly associated with AAC (**Supplementary Table S7**).

GWAS for phenolic compounds showed that three SNPs, ChrA10:16316355, ChrC4:1857195, and ChrC4:1891703, with relatively high PVE were significantly associated with gallic acid in the WW condition (**Supplementary Table S7**; **Supplementary Figure S3A**). A total of 179 SNPs distributed on chromosomes A01–A10, C02–C07, and C09 were significantly associated with caffeic acid in the WW condition (**Supplementary Table S7**; **Figures 5A, B**). The PVE ranged from 26.83% for the SNP ChrA4:8622635 to 33.50% for the SNP ChrA4:378678. The top 2 hot spots located on the 23–45505 Kbps region of chromosome A02 and 255–43967 Kbps interval of chromosome C02 comprised 35 and 38 SNPs, which covered 50.77% of phenotypic variation on their respective chromosomes (**Supplementary Table S7**; **Figures 5C–F**). Among the linked SNPs, ChrA4:10190551 showed the most significant association with caffeic acid under the WW condition (**Supplementary Table S7**). This linked SNP explained 33.01% of the phenotypic variation of caffeic acid. SNP ChrA4:378678 (PVE = 33.5%) and SNP ChrC4:8636542 (PVE = 32.44%) were also significantly associated with caffeic acid (**Supplementary Table S7**). Under the DS condition, five SNPs were significantly associated with the caffeic acid with PVE ranging from 24.71% to 30.96% (**Supplementary Table S8**; **Supplementary Figure S3D**).

Under the WW condition, the six SNPs including *Bn-A02-p27755843*, *Bn-A02-p25105527*, *Bn-A02-p26154897*, *Bn-scaff_17522_1-p1497074*, *Bn-A09-p28758854*, and *Bn-A09-p35981539* showing significant association with caffeic acid were further used to determine the effects of their individual alleles on caffeic acid (**Figures 5G–L**). The number of alleles for each of these six SNPs in the whole rapeseed population varied between 2 and 3. The two AG and GG alleles showed a significant difference in the regulation of caffeic acid content. For instance, the allele GG for the SNPs *Bn-A02-p27755843*, *Bn-scaff_17522_1-p1497074*, *Bn-A09-p28758854*, and *Bn-A09-p35981539* was associated with higher caffeic acid, whereas AG for these SNPs except *Bn-A09-p28758854* reduced this compound (**Figures 5G–L**).

The 36 significant SNPs associated with caffeic acid on ChrC2 under the WW condition were used for the identification of haplotype blocks on ChrC2 (**Figures 5M–P**). Three haplotype blocks (blocks 2–4) with strong LD (D′ >0.9) were mapped to 44, 2, and 284 Kbps, respectively (**Figures 5M–P**). In haplotype block 2 (41,808,767–41,853,503 bp), three haplotype alleles were identified that showed significant differences in the phenotypes of caffeic acid. Of these haplotypes, the haplotype alleles ACGAA (f = 0.504) and CAACG (f = 0.419) were associated with higher caffeic acid (**Figure 5N**). In haplotype block 3 (42,239,959–42,242,813 bp), four haplotype alleles showed significant phenotypic variation for caffeic acid. The haplotype allele AAGA with f = 0.686 was associated with higher caffeic acid (**Figure 5O**). Additionally, in haplotype block 4 (42,315,091–42,598,792 bp), the haplotype allele GAAGC regulated higher caffeic acid level (f = 0.686) (**Figure 5P**).

**Figure 7:**
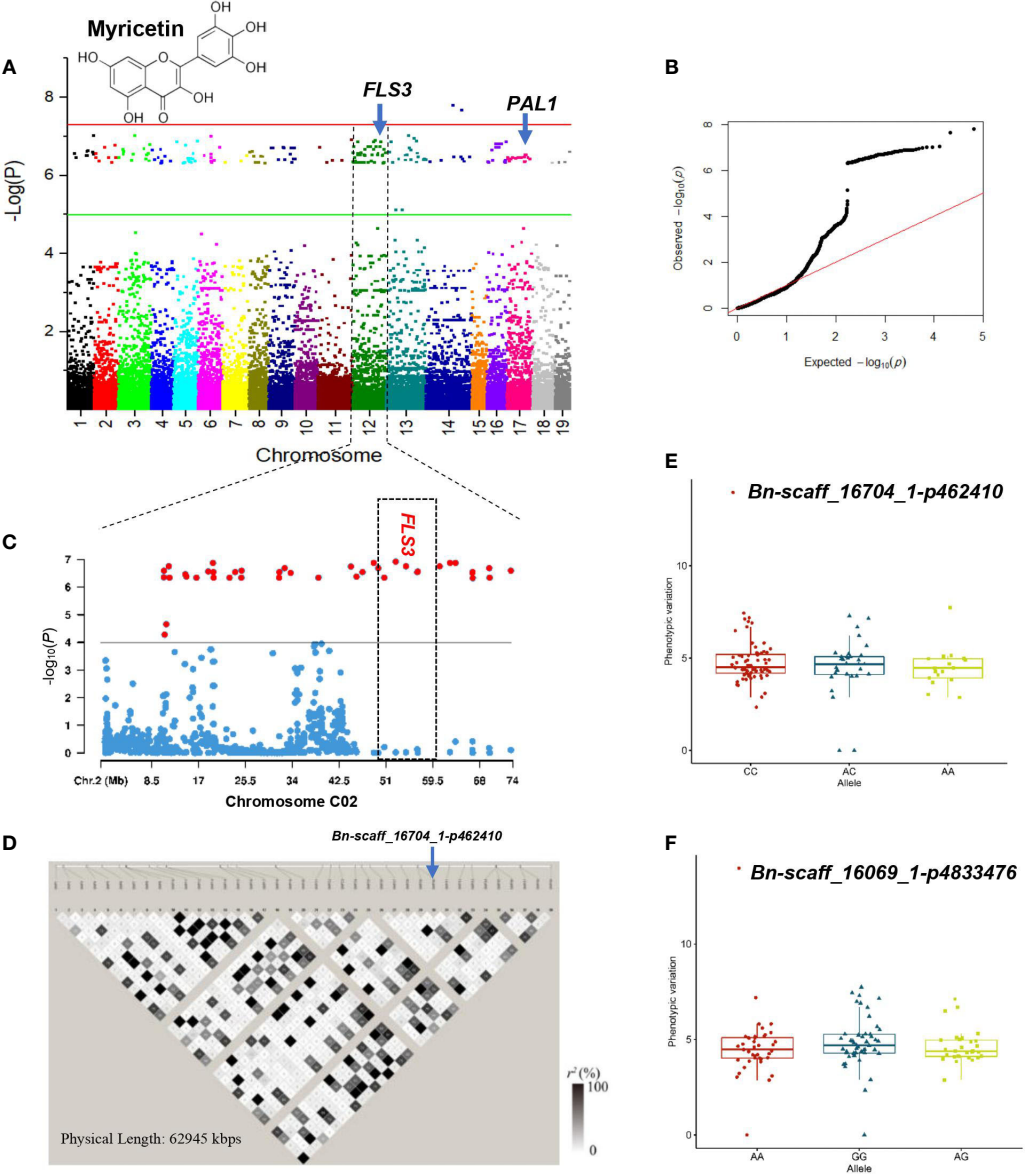
GWAS of myricetin using the 119 rapeseed (*Brassica napus*) genotypes. **(A)** Manhattan plot displaying the GWAS result of myricetin content in 19 chromosomes under drought stress condition. **(B)** QQ-plot for GWAS of myricetin content under drought stress condition. **(C)** Locus zoom plot for myricetin associations in the chromosome C02. **(D)** A representation of pairwise *r* ^2^ value (displayed as percentages) among polymorphic sites of chromosome 12 (C02). Allele-effect analysis for two significant SNPs including Bn-scaff_16704_1-p462410 **(E)** and Bn-scaff_16069_1-p4833476 **(F)**. The box plot depicts the number of the alleles for each of the significant SNPs in 119 rapeseed accessions, and the contribution of these alleles to the phenotypic variation observed for myricetin level.

In GWAS analysis, 89 significant SNPs were identified for the chlorogenic acid under the DS condition. The proportion of SNPs in total PVE (*R^2^*) ranged from 30.14% to 34.11%, with a mean of 30.92% (**Supplementary Table S8**; **Figures 7A, B**). Of them, 28 SNP–chlorogenic acid associations were identified on chromosome A06 that were located between 126616 and 74617 Kbps (**Figure 7C**). SNPs ChrA6:615333, ChrA6:12732383, and ChrA6:13700378 were the most significant SNPs associated with chlorogenic acid under the DS condition (**Supplementary Table S8**). Among the significant SNPs, 12 located on chromosome A09 were correlated with chlorogenic acid (**Supplementary Table S8**). The three SNPs on chromosome A06 and four on chromosome A07 were associated with higher chlorogenic acid content (**Figures 7D–F**).

Thirty-two SNPs on chromosomes A01-A04, A06, A07, A10, C01, C05, and C08 were significantly associated with coumaric acid under the DS condition (**Supplementary Table S8**; **Figures 7G, H**). The PVE of the SNP ranged from 21.49% to 48.87%. SNPs ChrA1:20708470 and ChrA1:73646490 significantly contributed to 46.86% of the PVE of coumaric acid. Allele AA for SNP *Bn-A02-p12044265* contributed to higher coumaric acid content (**Figure 7I**).

### Genetic basis of the flavonoid content under drought stress

Twenty-seven SNP–epicatechin associations on chromosomes A01, A05, A07–A10, C03–C05, and C07 (**Supplementary Table S7**) were detected under the WW condition. The PVE for the linked SNPs ranged from 24.22% to 33.01%. The most significant SNP ChrC4:29181029 had a PVE of 27.02% (**Supplementary Table S7**). Several SNPs associated with epicatechin were located on chromosome C05 and covered 43.15% of phenotypic variation. Three significantly linked SNPs, ChrC5:162937, ChrC5:163550, and ChrC5:163557, identified in an interval of 163 Kbps on chromosome C05 showed *P*-value = 9.34 × 10^–6^ (**Supplementary Table S7**). Under the DS condition, 29 SNPs were significantly associated with the epicatechin on chromosomes A01–A08, A10, C01–C05, C08, and C09 (**Supplementary Table S8**). The PVE for these SNPs ranged from 27.88% for SNP ChrC1:52661467 to 34.53% for SNP ChrC9:63810360. SNPs ChrC5:43670426, ChrC9:63810360, and ChrA7:28726499 showed stronger GWAS *P*-values in relation to epicatechin under the DS condition (**Supplementary Table S8**).

Association analysis at a threshold of *P<* 1.0 × 10^–6^ showed that 202 loci were associated with myricetin under the DS condition (**Supplementary Table S8**; **Figures 6A, B**). One hot spot located on the 10688–73679 Kbps region of chromosome C02 comprising 40 loci had a PVE of 34.77% (**Supplementary Table S8**; **Figures 6C, D**). Of the significant SNPs, SNP ChrC4:59722505 was the major locus controlling myricetin with a PVE of 40.98%. SNPs ChrC3:45787340 and ChrA6:43739339 explained 37.16% and 37.09% of the variation of myricetin, respectively (**Supplementary Table S8**). Alleles CC, AC, and AA for SNPs *Bn-scaff_16704_1-p462410* and *Bn-scaff_16069_1-p4833476* showed similar effects on myricetin content (**Figures 6E, F**).

### Characterization of the drought stress-related candidate genes

Two LD GRs showed 20 and 36 significant SNP–caffeic acid associations located on chromosomes A02 and C02, respectively, under the WW condition (**Figures 5E, F**). In the first GR, a highly significant LD (*r^2 = ^* 0.9) was found between SNPs ChrA2:426966 (tag-SNP #7) and ChrA2:444005 (tag-SNP #8). The position of the CG *DEAD-BOX ATP-DEPENDENT RNA HELICASE 25-LIKE* (*RH25*) was close to SNP ChrA2: 444005 (**Table 3** **;** **Figures 5C, E**). The *DEAD-box* genes play crucial roles in plant abiotic stress tolerance via regulating some stress-induced pathways. The *RH* genes are negative regulators of ABA signaling via inhibition of *PP2CA* activity. The functions of these RNA helicases influence the plant adaptive response (Baruah et al., 2017). The lead SNP ChrA2:23180627 was close to *E3 UBIQUITIN LIGASE* (*RSL1*) as a key regulator of hormone signaling (**Table 3** **;** **Figures 5C, E**). E3 ligases that transfer phytohormone abscisic acid (ABA) signals regulate plant responses to different abiotic stresses (Wang et al., 2022). SNP ChrA2:23154160 was close to *HMG-Y-RELATED PROTEIN A* (*HMGA*). High-mobility group (HMG) protein is an abundant non-histone chromosomal protein that promotes pollen tube development and regulates the expression of stress response genes (Xu et al., 2021). Several regulatory genes including TFs contribute to abiotic stress responses via regulating downstream stress-responsive genes (Joshi et al., 2016). We identified that the position of SNPs ChrA2:23778761 and ChrA2:24744845 was close to the position of TF CGs *NAC DOMAIN-CONTANING PROTEIN 35* (*NAC035*) and *ETHYLENE-RESPONSIVE TRANSCRIPTION FACTOR-LIKE* (*ERF119*), respectively (**Table 3** **;** **Figures 5C, E**). The TF family members involved in ABA-independent [ERF, bHLH, and NAM/ATAF1,2/CUC2 (NAC)] pathways have been upregulated in *B*. *napus* (Ke et al., 2020). The TFs of this family interact with specific cis-elements, and their overexpression confers stress tolerance (Yao et al., 2021). Moreover, SNP ChrA6:13700378 was close to *AUXIN-RESPONSIVE PROTEIN* (*SAUR71*).

**Table 3:** SNPs and candidate genes significantly associated with phenolic compounds integrating genome-wide association and transcriptome studies.

Phenoliccompounds	Lead SNP	Position (bp)^a^		Chr^b^	Candidate Gene^c^	Gene Location	Annotation
Drought stress-related genes							
Caffeic acid	Bn-A02-p27755843		444005	A2	LOC125581627	456830.460131	DEAD-box ATP-dependent RNA helicase 25-like (RH25)
	Bn-A02-p25105527		23180627	A2	LOC125587888	23161297-23165111	E3 ubiquitin-protein ligase (RSL1)
	Bn-A02-p25079431		23154160	A2	LOC125587885	23160045-23161004	Probable NAD(P)H dehydrogenase subunit CRR3, chloroplastic (CRR3)
	Bn-A02-p25105527		23180627	A2	LOC106424243	23185689-23190046	HMG-Y-related protein A (HMGA)
	Bn-A02-p26154897		23778761	A2	LOC106396214	23766736-23769871	NAC domain-containing protein 35 (NAC035)
	Bn-A02-p27055844		24377661	A2	LOC125580157	24382569-24383687	Cytosolic sulfotransferase 12-like (SOT12)
	Bn-A02-p27786739		24744845	A2	LOC111202999	24726755-24728300	Ethylene-responsive transcription factor (ERF119)
	Bn-scaff_15712_13-p38138		372866	C2	LOC106424063	370212-372418	Serine/threonine protein phosphatase 2A 57 kDa regulatory subunit B’ (B’ALPHA)
	Bn-scaff_17623_1-p837616		455566	C2	LOC106424074	454162-458783	Glutathione S-transferase T3 (GSTT3)
	Bn-scaff_17522_1-p1497074		5162738	C2	LOC106443306	5088531-5091807	F-box protein At1g30790-like (FBX)
Chlorogenic acid	Bn-A06-p3437409		12615333	A6	LOC106347668	12638041-12641569	Peptidyl-prolyl cis-trans isomerase (CYP71)
	Bn-A06-p3839293		13700378	A6	LOC106433452	13684571-13686129	Auxin-responsive protein (SAUR71)
Metabolite biosynthetic genes							
Caffeic acid	Bn-A09-p28758854		26690641	A9	LOC106367237	26877113-26878867	Cinnamoyl-CoA reductase 1 (CCR1)
	Bn-A09-p35981539		33104863	A9	LOC106412109	33233120-33235006	Chalcone-flavanone isomerase (CHI)
Chlorogenic acid	Bn-A07-p6542812		20717392	A7	LOC106430142	20512963-20517193	Flavonol 3-O-glucosyltransferase (UGT89B1)
Myricetin	Bn-scaff_16704_1-p462410		56681393	C2	LOC106382906	56510517-56512288	Flavonol synthase 3 (FLS3)
	Bn-scaff_16069_1-p4833476		52702475	C7	LOC106388514	53110580-53113485	Phenylalanine ammonia-lyase 1-like (PAL1)
Coumaric acid	Bn-A02-p12044265		16769326	A2	LOC106436650	16093088-16095189	Flavonoid 3’-monooxygenase (CYP75B137)

**^a^** Position in base pairs for the lead SNP according to version 4 of the rapeseed reference sequence, **^b^** Chromosome, **^c^** A plausible candidate gene near these loci.

In GR2, a significantly complete LD *r^2^* of 1 was found between 16 SNPs on chromosome C02 under the WW condition (**Figures 5D, F**). Highly significant LD (*r^2^* > 0.6) was found between SNPs ChrC2:5162738 (tag-SNP #5) and ChrC2:5172871 (tag-SNP #6) and between ChrC2:10513865 (tag-SNP #7) and ChrC2:10523601 (tag-SNP #8). SNP ChrC2:5162738 was 71 Kbps upstream of *F-BOX PROTEIN (FBX)* (**Table 3** **;** **Figures 5D, F**). F-box protein, as a major subunit of the Skp1, Cullins, F-box proteins (SCF) complex (a multiprotein E3 ubiquitin ligase), plays important roles in abiotic stress responses via the ubiquitin pathway (Song et al., 2023). The lead SNP ChrA6:12615333 was close to the *PEPTIDYL-PROLYL CIS-TRANS ISOMERASE* (*CYP71*) (**Table 3**; **Figure 7C**). Peptidyl-prolyl isomerase regulates protein folding and maturation of newly synthesized proteins under cell stress conditions (Hanes, 2015).

### Genes involved in phenolic acid and flavonoid biosynthetic pathways

Our GWAS analysis identified SNP ChrA9:26690641 for caffeic acid in the WW condition that was located at 187 Kbps downstream of *CINNAMOYL-COA REDUCTASE 1* (*CCR1*) gene. The *CCR1* gene is a potential control point in the phenylpropanoid pathway (**Table 3**; **Figure 5A**). *CCR1* involved in lignification uses feruloyl-CoA as a substrate and balances the abundance of phenolic compounds (Zhou et al., 2010). The linked SNP ChrC7:52702475 identified in the DS treatment was 409 Kbps downstream of *PHENYLALANINE AMMONIA-LYASE 1-LIKE* (*PAL1*) (**Table 3**; **Figure 6A**). *PAL* is the first enzyme in the phenylpropanoid pathway that catalyzes the trans-elimination of ammonia from l-phenylalanine to form trans-cinnamic acid, a precursor of lignins, phenols, flavanoids, and coumarins (Kong, 2015). SNP ChrA9:33104863 identified in the WW condition was located near *CHALCONE-FLAVANONE ISOMERASE* (*CHI*), a gene for flavonoid biosynthesis. Chalcone isomerase catalyzes the isomerization of naringenin chalcone into 5,7,4′-trihydroxyflavanone, which is considered to be the earliest intermediate with the flavonoid core (Ni et al., 2020). *CHI* genes co-regulate with other flavonoid-related genes, such as flavonol 3-O-glucosyltransferase, flavonol synthase, and flavonoid 3’-monooxygenase (Xu et al., 2023). Our GWAS results indicated that the lead SNP ChrA7:20717392 identified as a linked SNP in the DS treatment was 200 Kbps upstream of *FLAVONOL 3-O-GLUCOSYLTRANSFERASE* (*UGT89B1*) (**Table 3**; **Figure 7A**). Flavonol 3-O-glucosyltransferase catalyzes the glycosylation of either anthocyanidins or flavonols and is considered as a key enzyme for flavonoid modification (Dai et al., 2017). Glycosylation is usually the last step in the flavonoid biosynthetic pathway, which allows and enables plants to better cope with biotic and abiotic stress including the decrease of toxicity and side effects with the help of glycosylated phenolic compounds (Zhang et al., 2013). The LD GRs were estimated for 39 significant SNP–myricetin associations mapped on chromosome C02 in the DS treatment (**Figure 6D**). Significant and complete LD (*r^2 = ^* 1) was found between the two SNPs ChrC2:19721386 (tag-SNP #10) and ChrC2:22620484 (tag-SNP #11), and a highly significant LD (*r^2^* > 0.9) was found between SNP ChrC2:62628403 (tag-SNP #32) and SNP ChrC2:63618405 (tag-SNP #33). The LD plot showed that SNP ChrC2:56681393 was 200 Kbps upstream of *FLAVONOL SYNTHASE 3* (*FLS3*) under drought (**Table 3** **;** **Figures 6C, D**). *FLS* genes belong to the dioxygenases that catalyze the oxidation of dihydroflavonol to produce flavonol (Wang et al., 2021). The lead SNP ChrA2:16769326 identified as a linked SNP under drought was 675 Kbps upstream of *FLAVONOID 3’-MONOOXYGENASE* (*CYP75B137*).

### Transcriptomes of the high-phenolic (G67) and low-phenolic (G59) genotypes

**Figure 8:**
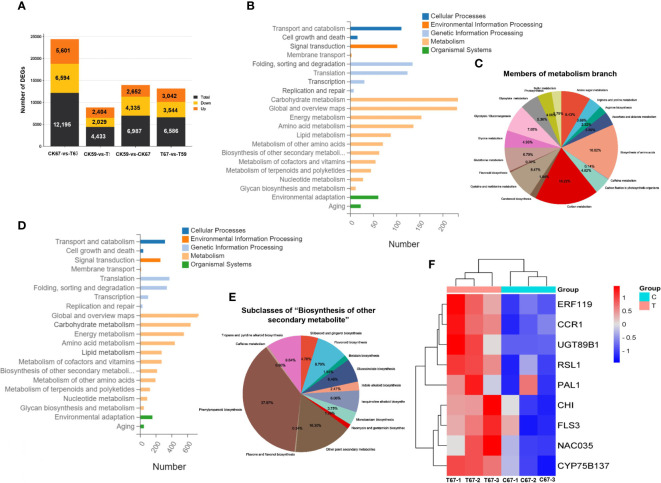
KEGG pathway classification map. **(A)** The number of up‐ and down‐regulated DEGs in high-phenolics genotype (G67) and low-phenolics genotype (G59) after drought. (CK): Control and (T): drought treatment. **(B)** KEGG pathway classification map in low-phenolics genotype (G59). Genes were divided into five branches according to the biological pathways they participated in: Cellular Processes, Environmental Information Processing, Genetic Information Processing, Metabolism, Organismal Systems. **(C)** A pie chart showing the members of metabolism branch. **(D)** KEGG pathway classification map in high-phenolics genotype (G67). Genes were divided into five branches according to the biological pathways they participated in: Cellular Processes, Environmental Information Processing, Genetic Information Processing, Metabolism, Organismal Systems. **(E)** A pie chart showing subclasses within the “Biosynthesis of other secondary metabolite”. **(F)** Heatmap expression of drought response and flavonoid biosynthesis genes under drought stress in transcriptome data in G67; (C): Control and (T): drought treatment. Heatmap color represents the expression level of each gene (rows) under drought treatment. (Fold-change > 1, P-value < 0.05). Red bars: upregulation; blue bars: downregulation.

Among the 119 genotypes evaluated in the two conditions, G67 and G59 showed the highest and lowest phenolic contents, respectively. To identify genes potentially involved in drought and flavonoid biosynthesis pathway, we conducted an RNA-seq workflow on flower extracts of both G67 and G59 genotypes. In total, we obtained 90,573,446 clean reads, of which 79.33% of the reads mapped to the *B*. *napus* reference genome. DEGs were identified with the criteria of *q* ≤ 0.05 and log_2_ fold change ≥1. The high-phenolic genotype G67 and low-phenolic G59 showed a substantial difference in their basal transcriptomes; a comparison of untreated G67 and G59 samples led to the identification of 6,987 DEGs, including 2,652 upregulated DEGs and 4,335 downregulated DEGs (**Figure 8A**). The high-phenolic genotype G67 had a total of 12,195 DEGs, of which 5,601 were upregulated. By contrast, the low-phenolic genotype had only 4,433 DEGs. Under DS, G67 showed more upregulated DEGs compared to G59 (T67 vs. T59; **Figure 8A**). We assumed that G67 might have numerous drought tolerance-related genes that are primed for response to DS.

To gain insight into the biological processes that were commonly or uniquely overrepresented between G59 and G67 in response to DS, we analyzed Gene Ontology (GO) and Kyoto Encyclopedia of Genes and Genomes (KEGG) enrichment of DEGs. The upregulated common biological processes included cellular processes, environmental information processing, genetic information processing, and metabolism, organismal systems (**Figures 8B–E**). The upregulated DEGs in G67 were specifically enriched in “phenylpropanoid biosynthesis,” “flavone and flavonol biosynthesis,” and “other plant secondary metabolites” (**Figure 8E**).

### Integration of the GWAS and transcriptome data

**Figure 9:**
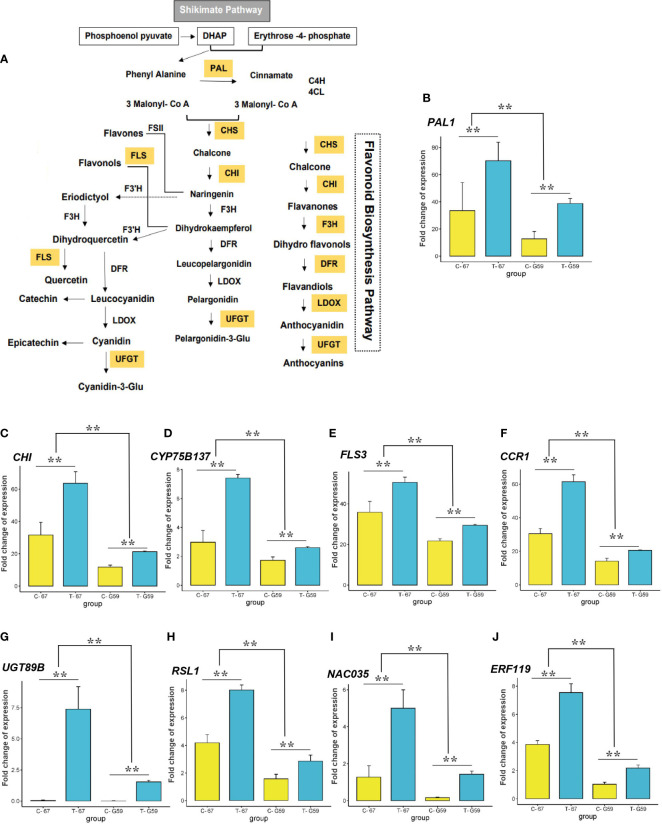
Expression variation of candidate genes from GWAS and transcriptome analysis. **(A)** Schematic diagram of the flavonoid biosynthesis pathway. PAL phenylalanine ammonia lyase, C4H cinnamate 4-hydroxylase, 4CL 4-coumarate-CoA ligase, CHS chalcone synthase, CHI chalcone isomerase, F3H flavanone-3-hydroxylase, F3'H flavonoid 3'-hydroxylase, FSII flavone synthase II, FLS flavonol synthase, DFR dihydroflavonol reductase, LDOX leucoanthocyanidin dioxygenase, UFGT UDP-glucose flavonoid 3-O-glucosyl transferase are key enzymes involved in the pathway. **(B–J)** Expression (Fold-change) of PAL1, CHI, CYP75B137, FLS3, CCR1, UGT89B, RSL1, NAC035, and ERF119 in high-phenolics genotype (G67) and low-phenolics genotype (G59) at the flowering stage under drought treatment (T), detected by RNA-seq. Data are average values with standard deviation (n = 3 varieties). ** Indicate significant difference relative to control (C) (t-test: **P < 0.01).

Our aim was to identify CGs differentially expressed after drought that were detected in the GWAS. Among the 60 genes detected in the GWAS, 50 were expressed in rapeseed flower based on transcriptome data. Of these genes, 18 were involved in stress-induced pathways, phenylpropanoid pathway, and flavonoid modifications (**Figure 8F**; **Table 3**). Analysis of the expression pattern of genes showed that the early unbranched part of the flavonoid biosynthesis pathway is encoded by *CHI*, *PAL*, and *CYP75B137* genes (**Figure 9A**). A significant increase in *PAL* was observed under drought (2.09-fold). The level of induction of gene expression was genotype-dependent. The relative expression of *PAL* was much higher in the high-phenolic genotype (G67) than in low ones (G59) (**Figure 9B**). The *CHI* gene also exhibited higher expression (2.01-fold) compared to the control (**Figure 9C**). The *CYP75B137* gene also showed a higher expression (2.48-fold) compared to the control (**Figure 9D**). The biosynthesis of flavonoids in the later stages is encoded by *FLS1/FLS3*, *CCR1*, and *UGT89B1* genes. The expression of the *FLS3* gene was higher (1.40-fold) in drought than in the control (**Figure 9E**). Additionally, under DS, the expression level of *CCR1* was much higher (2.01-fold; **Figure 9F**). The transcript level of *UGT89B1* was elevated more than 200-fold in the high-flavonoid genotype under water deficit conditions (**Figure 9G**). The transcript level of flavonoid biosynthesis genes was directly related to the flavonoid content. As shown in **Figures 9B–G**, the expression level of all flavonoid-related genes was much higher in G67 than that in G59. Transcriptional analysis showed that the genes were differentially expressed between the two genotypes, of which the *RSL1*, *NAC035*, and *ERF119* genes were upregulated under drought (**Figures 9H–J**).

## Discussion

### Phenolic compounds in *B. napus* flowers

In recent years, phenolic compounds have been intensively investigated because of their potential health-promoting effects (Cosme et al., 2020; Rahman et al., 2021). Phenolic compounds are considered in *Brassica* as the most abundant group of secondary metabolites known as natural antioxidants (Bennouna et al., 2021). This group of compounds is able to protect cells against the negative effects of ROS, lipid peroxidation, protein denaturation, DNA damage, and consequently reducing the oxidative damage (Jideani et al., 2021; Juan et al., 2021). Moreover, phenolic compounds have an important role in pigmentation, growth, reproduction of plants, and elevated defenses against the adverse effects of environmental stresses (Cheynier et al., 2013; Naik and Al-Khayri, 2016). In the present study, we found that *B*. *napus* flowers are a rich source of total phenolic, flavonoid, and flavonol content, anthocyanin, ascorbic acid, and natural antioxidants. The major dihydroxybenzoic acid in rapeseed flowers is gentisic acid (2,5-dihydroxybenzoic acid). In addition, caffeic acid (3,4-dihydroxy cinnamic acid) and epicatechin identified as abundant compounds in our study are the most important compounds of hydroxycinnamic acid and flavonoids. Notably, rapeseed flowers offer phenolic and flavonoid in much higher quantities than seed (Jun et al., 2014; Salami et al., 2023). Our results indicated that *B*. *napus* flowers can serve as natural antioxidants as characterized by a higher inhibition of DPPH (93%), which was higher than 83.17% and 89.25% DPPH activity in *B*. *juncea* (Lee et al., 2020) and *B*. *nigra*, respectively (Olgun et al., 2017). Phenolic compounds are often perceived favorably by consumers as healthy natural foods. Therefore, rapeseed flower can be used as a source of phenolic compounds with bioactive potential, which can be applied in the food sector, as food and as source of natural ingredients that can be an interesting alternative for consumers looking for product innovations.

### Drought stress responses in *B*. *napus* flowers

DS affects flowering and flower metabolism in rapeseed (Tesfamariam et al., 2010; Kirkegaard et al., 2018). There are various reasons for drought-induced damage of the flowering stage, including decreased water potentials in the floral tissues such as pollen, female gametophyte, and stigma and decreased assimilate and nitrogen supplies that ultimately affect the rapeseed yield (Fard et al., 2018; Zhu et al., 2021; Batool et al., 2022). Another phenomenon can occur, which is that drought-induced hormonal changes may cause sterility (Lalay et al., 2022). One of the adverse effects of DS at the molecular level is the accelerated production of ROS that disrupts the physiological and biochemical processes in plants (Saha et al., 2022). Plants have endogenous mechanisms for adapting to ROS production through enhancement of the natural activities of the antioxidant enzymes and nonenzymatic compounds (Fujita and Hasanuzzaman, 2022; Jan et al., 2022). Among the nonenzymatic antioxidant components, ascorbic acid, anthocyanin, and phenolic compounds are effective in resistance and adaptive features in plants against DS through suppression of singlet oxygen and inhibition of ROS-generating enzymes (Ashraf et al., 2022; Celi et al., 2023; Wagay et al., 2023). In this study, we monitored the changes in phenolic compounds between two contrasting irrigation conditions and evaluated their contributions to drought tolerance. The phenolics, flavonoids, and phenolic compounds were increased under drought condition in our rapeseed population. A significant enhancement of epicatechin and myricetin contents was observed in rapeseed flowers under DS conditions. The results of PCA and stepwise regression showed that myricetin had strong associations with antioxidant activity under DS conditions. These results suggest that epicatechin and myricetin play an important role in enhancing drought tolerance in *B*. *napus*. Similarly, Okafor et al. (2022) reported that flavonoids had a stronger antioxidant activity than phenolic acids. Flavonoids like myricetin help the formation of less reactive phenoxyl radicals by donation of electrons or hydrogen and the ability to chelate transition metals like iron (Semwal et al., 2016). The iron-chelating properties and RSA of flavonoids are closely related; iron is chelated by the flavonoid and ROS are subsequently scavenged by the flavonoids (Kejik et al., 2021; Agraharam et al., 2022). Transcriptomic and metabolomic analyses have shown that flavonoid accumulation is important to improve drought tolerance in *B*. *napus* (Xiong and Ma, 2022). An increase in phenolic content in rapeseed tested under water scarcity has been shown in several studies (Son et al., 2020; Okafor et al., 2022; Morsi et al., 2023). Information on the response of phenolic compounds and their antioxidant activity to DS will be essential for producers to understand the advantages of this crop for cultivation under water scarcity conditions.

### Stable SNPs identified for phytochemicals and phenolics

Information on DNA markers linked with polyphenol components contributing to DS helps screening varieties for drought tolerance improvement. The stability of linked markers over different environmental conditions and pleiotropic effects are two important factors for the use of markers in MAS programs and improvement of crops. In our GWAS analysis, evaluation of the relation between SNPs and traits showed several common and pleiotropic SNPs. Two SNPs for RSA and one for TFLC that were common between the two irrigation treatments significantly contributed to phenotypic variation of these traits. The SNP Bn-A01-p6333910 had pleiotropic effects on both chlorogenic and coumaric acid content under DS conditions. These stable and pleiotropic SNPs with high contribution to phenotypic variation for phenolic content provide valuable genetic information for the improvement of phenolics and indicate promising GR for MAS in rapeseed breeding programs.

### Candidate genes involved in phenolic biosynthesis pathway in *B*. *napus*

All phenolic compounds are synthesized through the shikimic acid/phenylpropanoid pathway (Kumar et al., 2023; **Figure 9A**). The synthesis of phenylalanine from chorismic acid is considered the branching point that links the shikimate pathway to the phenylpropanoid pathway (Tohge et al., 2013). The general phenylpropanoid pathway from phenylalanine to p-coumaroyl-CoA is considered a major precursor in the synthesis of both hydroxycinnamic acids and flavonoids (Metsamuuronen and Siren, 2019). The early phenylpropanoid pathway involves three key enzymes: PAL, C4H, and 4CL (Zhang and Liu, 2015; **Figure 9A**). PAL as a key enzyme in primary metabolism catalyzes the non-oxidative deamination of phenylalanine to cinnamic acid and affects the accumulation of lignin, flavonoids, and hydroxycinnamic acid through regulating the rate of phenylalanine entering the phenylpropanoid pathway (Wu et al., 2017). In this study, the drought-related GWAS results consistent with results of a study in *B*. *napus* (Ali et al., 2023) showed strong associations between *PAL1* and myricetin under drought conditions (**Figure 6A**, **Supplementary Table S8**). Moreover, the expression of *PAL1* increased in our highly accumulated myricetin accession and under drought conditions. Our RNA-seq analysis indicated that the stress-inducible expression of the *PAL1* gene might increase the accumulation of flavonoids and enhance abiotic stress tolerance in rapeseed.

CHI as the first enzyme specific for the flavonoid pathway converts naringenin chalcone to naringenin flavanone, which is a branching point for the formation of several groups of flavonoids (Yonekura-Sakakibara et al., 2019). *CHI* is co-regulated with other flavonoid genes, such as *CHS*, *F3H*, and *DFR* (Kayani et al., 2021). The genetic control of CHI activity has been assessed in flowers and pollen of several plant species (Sun et al., 2019). In accord with results of Hao et al. (2022), a higher *CHI* expression was reported under DS in our study. In response to higher *CHI* expression levels under drought, a higher caffeic acid was observed in the high phenolic accession (**Figure 9C**). Another early flavonoid biosynthetic gene, *FLAVONOID 3’-MONOOXYGENASE* (*CYP75B1*), is mostly involved in monooxygenation/hydroxylation and regulation of antioxidant metabolites (Zandalinas et al., 2017). It has been shown that the *CYP75B1* gene positively correlates with flavonoids and antioxidant flavonol accumulation and induces drought tolerance in *Arabidopsis* (Rao et al., 2020). In our GWAS and RNA-seq analyses, *CYP75B137* was upregulated under drought and associated with coumaric acid, which suggest a protective role of this gene against the adverse effect of drought. In our study, the induction of *FLS3* was quite significant under DS (**Figure 9E**), which possibly indicates the role of higher *FLS3* expression in drought tolerance in rapeseed. FLS catalyzes the oxidation of dihydroflavonol to produce flavonol, which is widely distributed in flowering plants (Yang et al., 2022; **Figure 9A**). So far, 13 *FLS* genes have been identified for *B. napus* (Schilbert et al., 2021). Higher FLS transcripts under DS have been shown in rapeseed (Liu et al., 2015) and *Arabidopsis* (Li et al., 2018).

Glycosylation is one of the most widespread modifications of flavonoids as the last step in the flavonoid biosynthetic pathway in plants (Alseekh et al., 2020). The UDP-glycosyltransferase (UGT) is a key enzyme in flavonoid modification used for transferring UDP-activated sugars to flavonoid compounds and increasing their solubility and stability (Irmisch et al., 2018). Results of GWAS of our study supported that the *UGT89B1* gene was associated with chlorogenic acid content and the expression of *UGT89B1* was strongly induced by DS (**Figure 9G**). These observations were in agreement with results of a study in *Camellia sinensis* for downregulation of *UGT* enzymes that reduced flavonol glycoside accumulation, ROS detoxification capacity, and lower stress tolerance (Zhao et al., 2019). Our results indicated that the glucosylation catalyzed by *UGT89B1* was effective for drought tolerance in rapeseed through the improvement in the biosynthesis of phenolics.

### Key regulators of phenolic biosynthesis pathway in *B*. *napus*

Biosynthetic enzymes, TFs, phytohormonal regulators, and environmental conditions affect phenylpropanoid biosynthesis and the structural genes (Guo et al., 2020). Different families of TFs including MYB, bHLH, bZIP, NAC, AP2/ERF, and WRKY regulate the expression of the structural genes in the phenylpropanoid pathway (Anwar et al., 2021). The plant-specific NAC proteins constitute one of the largest TF families and are characterized by a highly conserved NAC domain in the N-terminal region (Puranik et al., 2012). Although it has been widely demonstrated that the NAC TFs are involved in the regulation of various biological processes, from plant development to response to stresses, only a few members have been identified as regulators of anthocyanin biosynthesis (Sun et al., 2019). To date, some studies have focused on the regulation of *NAC* genes in anthocyanin biosynthesis (He et al., 2020). For example, *NAC078* protein was associated with the expression of genes related to flavonoid biosynthesis and leads to the accumulation of anthocyanins in response to high light stress in *Arabidopsis* (Morishita et al., 2009). Results of GWAS in our study showed that the *NAC035* gene was associated with caffeic acid content. Moreover, transcription of *NAC035* that was enhanced by DS was higher in the high-phenolic rapeseed genotype in our study, which was in line with the results of *NAC035* expression in drought in the study by Li et al. (2022). *NAC035* affects the growth and developmental processes from cell division to flower development in response to external signals in plants (Li et al., 2022). We found that the *NAC035* gene could be a positive regulator of flower phenolics in rapeseed. *CHI*, *FLS3*, and *CYP75B137* were upregulated in the higher-phenolic rapeseed under drought conditions, which shows that the expression of these genes under DS is regulated not only by the structural genes but also by TFs.

As one of the largest TF families in plants, AP2/ERF (APETALA2/Ethylene Responsive Factor) plays indispensable roles in plant growth, development, hormone regulation, flavonoid synthesis, and especially in responses to various stresses (Zhang et al., 2020; Qian et al., 2022; Qi et al., 2023). Recently, three AP2/ERF TFs were identified as positive regulators of the chalcone isomerase (CHI) gene that enhanced the accumulation of flavanones and flavones in citrus (Zhao et al., 2021). In our study, the *ERF119* gene was associated with caffeic acid content and upregulated under drought and in high-phenolic rapeseed accession. In a study in *Mangifera indica*, *ERF109* promoted the transcription of anthocyanin-related genes and the anthocyanin accumulation (Qian et al., 2022). Under stress, ERFs recognize and bind to cis-regulatory elements in the promoters of target genes to participate in gene expression regulation (Seok et al., 2022). Therefore, our results in line with other studies showed that *ERF119* improves drought tolerance in rapeseed by regulating phenolic-related genes.

## Conclusion

In the present study, a conjoint analysis based on metabolome and transcriptome data of rapeseed flowers was performed to identify the key metabolites, genes, and metabolic pathways related to drought response in rapeseed. We identified new sources of variation that contributed to higher polyphenolic compounds in rapeseed under DS conditions. Our work identified genomic locations within *B*. *napus* that influence the expression of phytochemicals and polyphenolic compounds under DS conditions. This work provides supporting information for two research areas. One is the development of breeding strategies for drought tolerance by disentangling the molecular mechanisms of these traits. The other is to develop genomic resources for developing metabolite-associated breeding in the future. The genomic and metabolomic variability identified in our selected rapeseed led to the detection of SNP-linked markers across the rapeseed genome that could help to facilitate the metabolite-associated breeding of rapeseed in the future. The use of metabolomic, genomic, and transcriptomic approaches increased our confidence in the identification of related genes and markers through GWAS. This workflow enabled the detection of six CGs including *PAL1*, *CHI*, *UGT89B1*, *FLS3*, *CCR1*, and *CYP75B137* associated with major phenolic acids and flavonoids. The analysis of the molecular mechanisms of drought response-related genes will contribute to the cultivation and development of new rapeseed drought-tolerant varieties, providing good materials for cultivating water-saving plants.

## Data availability statement

Publicly available datasets were analyzed in this study. This data can be found here: https://www.ncbi.nlm.nih.gov/sra/?term=PRJNA1052984, https://www.ncbi.nlm.nih.gov/geo/query/acc.cgi?&acc=GSE250611.

## Author contributions

MS performed the experiments, prepared samples, analyzed the data, and wrote original draft of the manuscript; BH designed the research and the experiments, prepared samples, wrote and reviewed the first and final draft of the manuscript; JB involved in experiments and reviewed the article; JW involved in the experiments and reviewed the article; XT involved in the experiments and reviewed the article; CR and HT provided scientific comments and reviewed the final draft of the manuscript. All authors contributed to the article and approved the submitted version.
