# Supplementary material for: Integration of genome-wide association studies, metabolomics, and transcriptomics reveals phenolic acid- and flavonoid-associated genes and their regulatory elements under drought stress in rapeseed flowers
Source: Front Plant Sci. 2024 Jan 11;14:1249142. doi: 10.3389/fpls.2023.1249142 (PMC10808681; doi:10.3389/fpls.2023.1249142)
Supplement: Supplementary file 1 [file DataSheet_1.pdf]

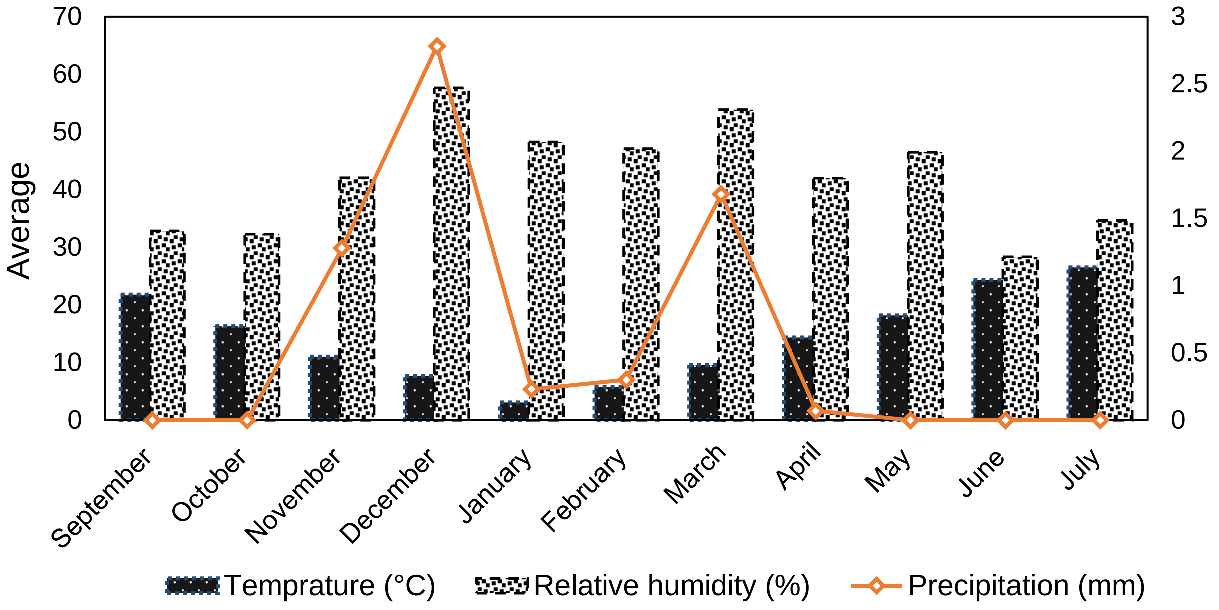

Supplementary Fig. S1. Monthly mean temperature, monthly mean values of relative humidity, and monthly mean precipitation during the rapeseed (*Brassica napus*) growing season in 2020-2021.
