# Supplementary material for: Integration of genome-wide association studies, metabolomics, and transcriptomics reveals phenolic acid- and flavonoid-associated genes and their regulatory elements under drought stress in rapeseed flowers
Source: Front Plant Sci. 2024 Jan 11;14:1249142. doi: 10.3389/fpls.2023.1249142 (PMC10808681; doi:10.3389/fpls.2023.1249142)
Supplement: Supplementary file 2 [file DataSheet_2.pdf]

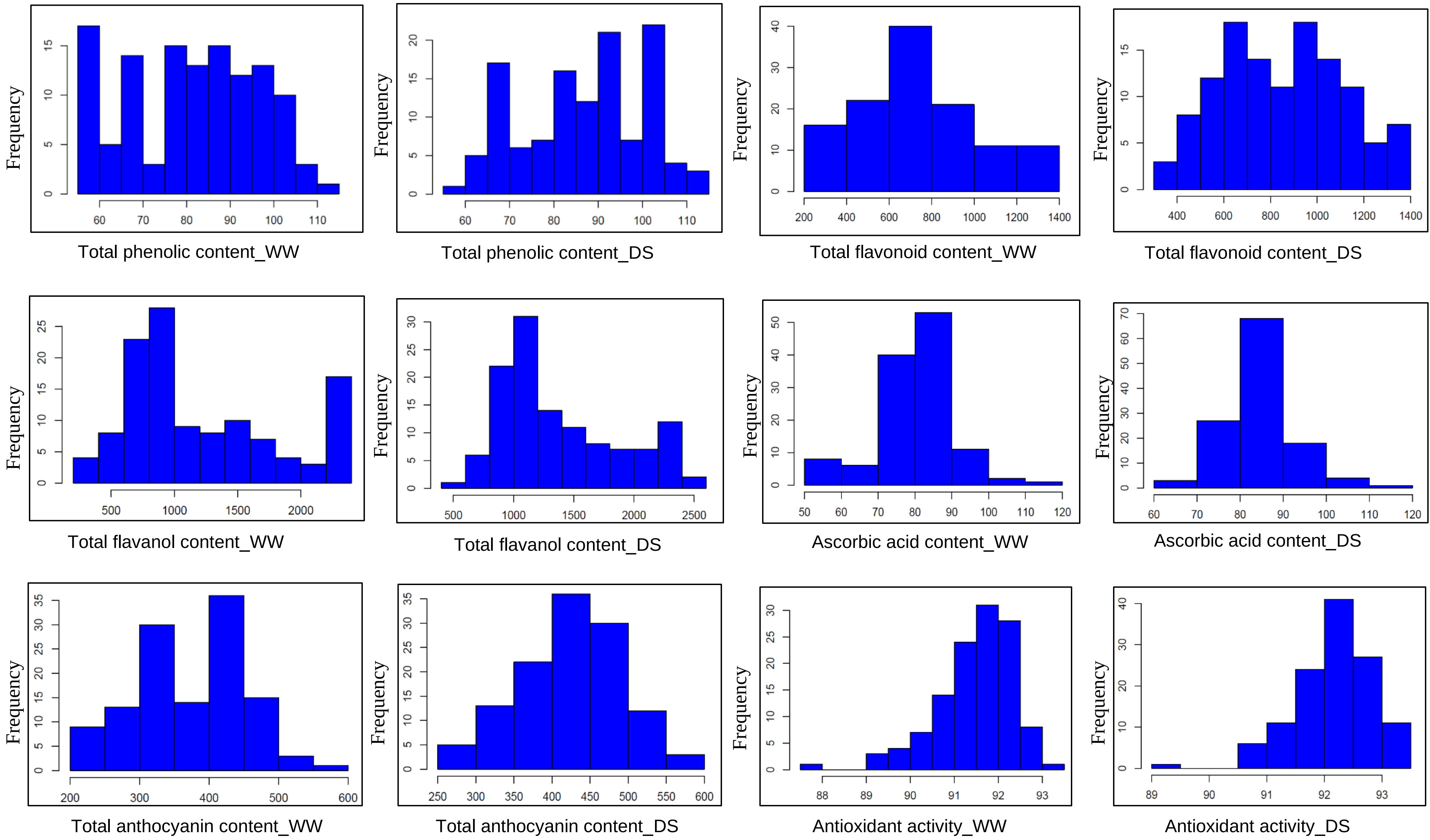

Supplementary Fig. S2. Phenotype distribution and boxplots of phytochemical traits in the associated population of rapeseed (*Brassica napus*) under well-watered (WW) and drought stress (DS) conditions.
