# Supplementary material for: Integration of genome-wide association studies, metabolomics, and transcriptomics reveals phenolic acid- and flavonoid-associated genes and their regulatory elements under drought stress in rapeseed flowers
Source: Front Plant Sci. 2024 Jan 11;14:1249142. doi: 10.3389/fpls.2023.1249142 (PMC10808681; doi:10.3389/fpls.2023.1249142)
Supplement: Supplementary file 3 [file DataSheet_3.pdf]

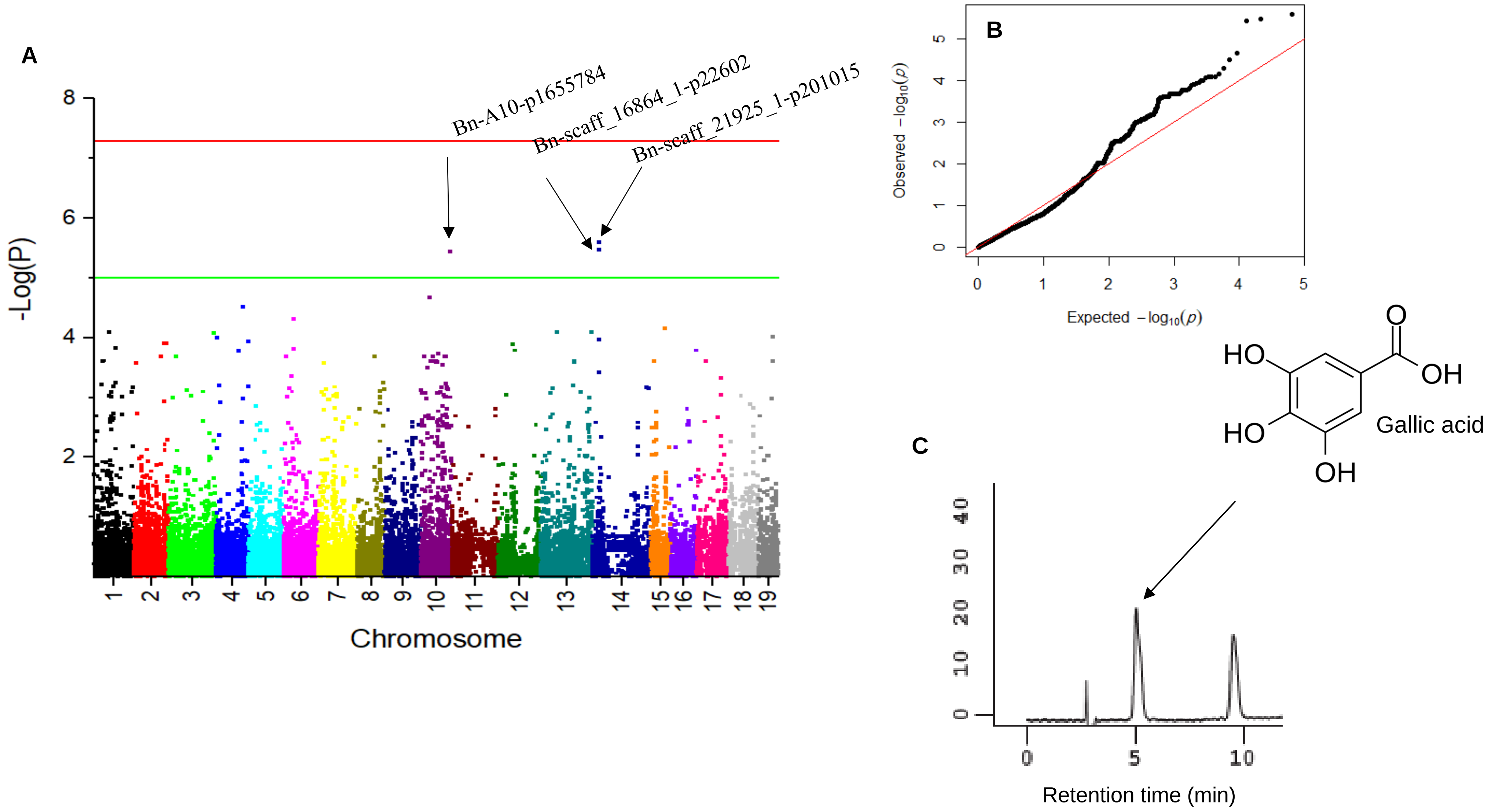
A
Bn-A10-p1655784
Bn-scaff_16864_1-p22602
Bn-scaff_21925_1-p201015
8
6
4
2
-Log(P)
1 2 3 4 5 6 7 8 9 10 11 12 13 14 15 16 17 18 19
Chromosome
B
Observed $-\log_{10}(p)$
Expected $-\log_{10}(p)$
0 1 2 3 4 5
C
O
HO
OH
HO
OH
Gallic acid
0 10 20 30 40
0 5 10
Retention time (min)

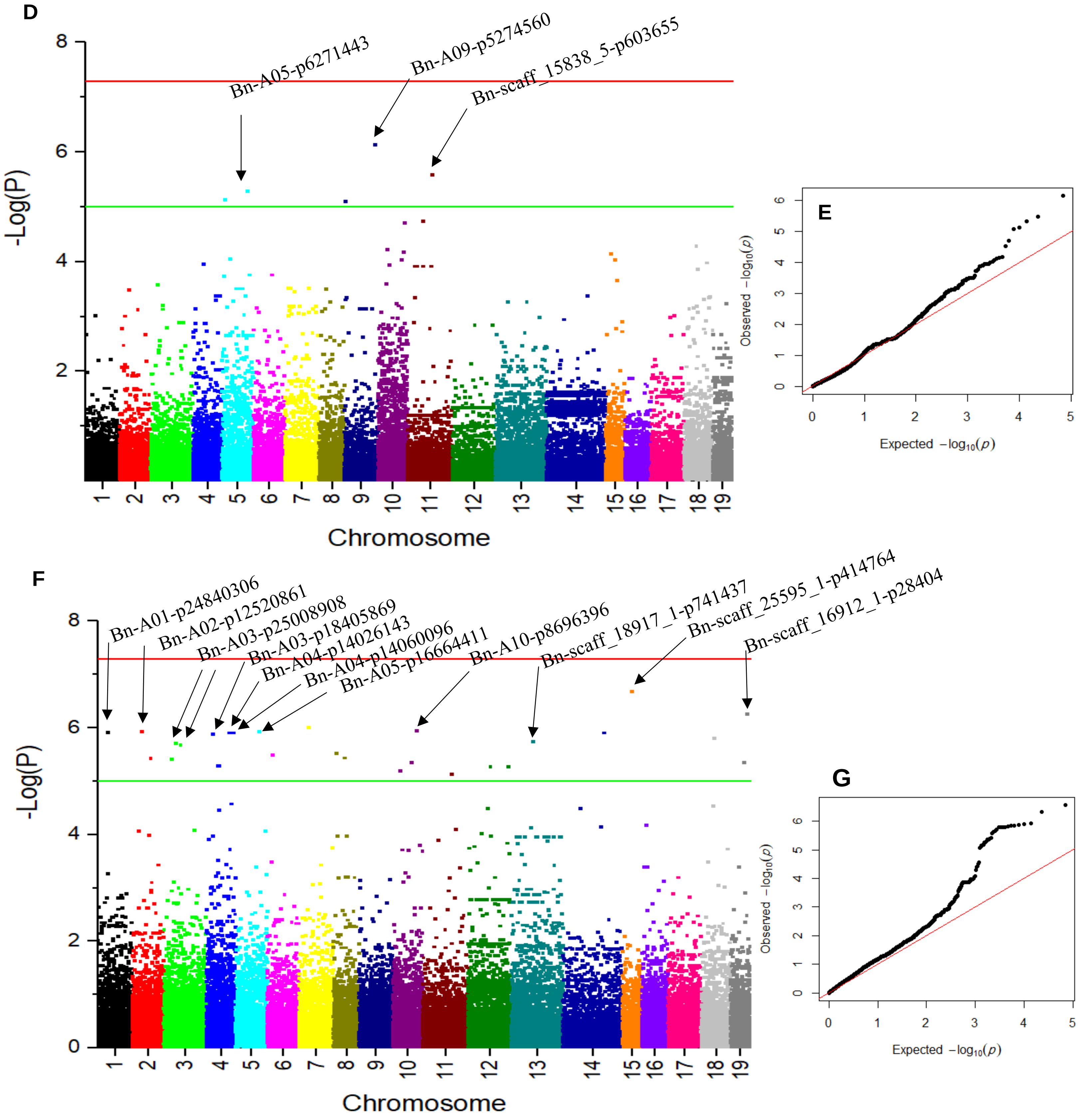
D
Bn-A05-p6271443
Bn-A09-p5274560
Bn-scaff_15838_5-p603655
-Log(P)
Chromosome
E
Observed $-\log_{10}(p)$
Expected $-\log_{10}(p)$
F
Bn-A01-p24840306
Bn-A02-p12520861
Bn-A03-p25008908
Bn-A03-p18405869
Bn-A04-p14026143
Bn-A04-p14060096
Bn-A05-p16664411
Bn-A10-p8696396
Bn-scaff_18917_1-p741437
Bn-scaff_25595_1-p414764
Bn-scaff_16912_1-p28404
-Log(P)
Chromosome
G
Observed $-\log_{10}(p)$
Expected $-\log_{10}(p)$

Supplementary Fig. S3. Manhattan and QQ-plots for GWAS of gallic acid content under well-watered condition and caffeic acid and epicatechin under drought stress. (**A**) Manhattan plot displaying the GWAS result of gallic acid content in 19 chromosomes under well-watered condition. SNPs on different chromosomes are denoted by different colors. (**B**) The corresponding quantile-quantile (QQ) plot for GWAS of gallic acid content under well-watered condition. (**C**) The HPLC chromatograms for gallic acid. (**D**) Manhattan plot displaying the GWAS result of caffeic acid content in 19 chromosomes under drought stress condition. (**E**) QQ-plot for GWAS of caffeic acid content under drought stress condition. (**F**) Manhattan plot displaying the GWAS result of epicatechin content in 19 chromosomes under drought stress condition. (**G**) QQ-plot for GWAS of epicatechin content under drought stress condition.
