# Supplementary material for: Integration of genome-wide association studies, metabolomics, and transcriptomics reveals phenolic acid- and flavonoid-associated genes and their regulatory elements under drought stress in rapeseed flowers
Source: Front Plant Sci. 2024 Jan 11;14:1249142. doi: 10.3389/fpls.2023.1249142 (PMC10808681; doi:10.3389/fpls.2023.1249142)
Supplement: Supplementary file 4 [file DataSheet_4.pdf]

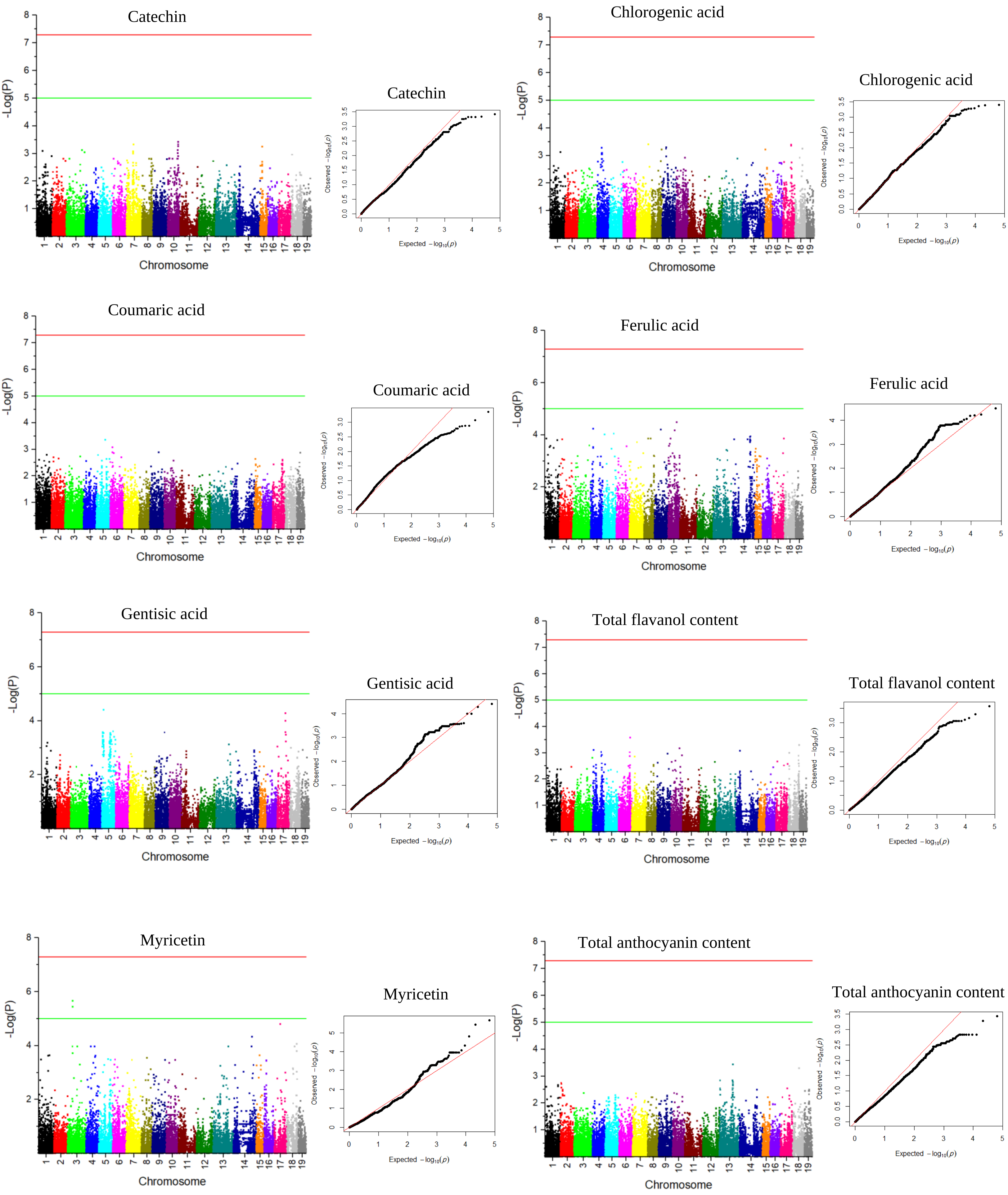
Catechin
-Log(P)
Chromosome
Catechin
Observed −log10(p)
Expected −log10(p)
Chlorogenic acid
-Log(P)
Chromosome
Chlorogenic acid
Observed −log10(p)
Expected −log10(p)
Coumaric acid
-Log(P)
Chromosome
Coumaric acid
Observed −log10(p)
Expected −log10(p)
Ferulic acid
-Log(P)
Chromosome
Ferulic acid
Observed −log10(p)
Expected −log10(p)
Gentisic acid
-Log(P)
Chromosome
Gentisic acid
Observed −log10(p)
Expected −log10(p)
Total flavanol content
-Log(P)
Chromosome
Total flavanol content
Observed −log10(p)
Expected −log10(p)
Myricetin
-Log(P)
Chromosome
Myricetin
Observed −log10(p)
Expected −log10(p)
Total anthocyanin content
-Log(P)
Chromosome
Total anthocyanin content
Observed −log10(p)
Expected −log10(p)

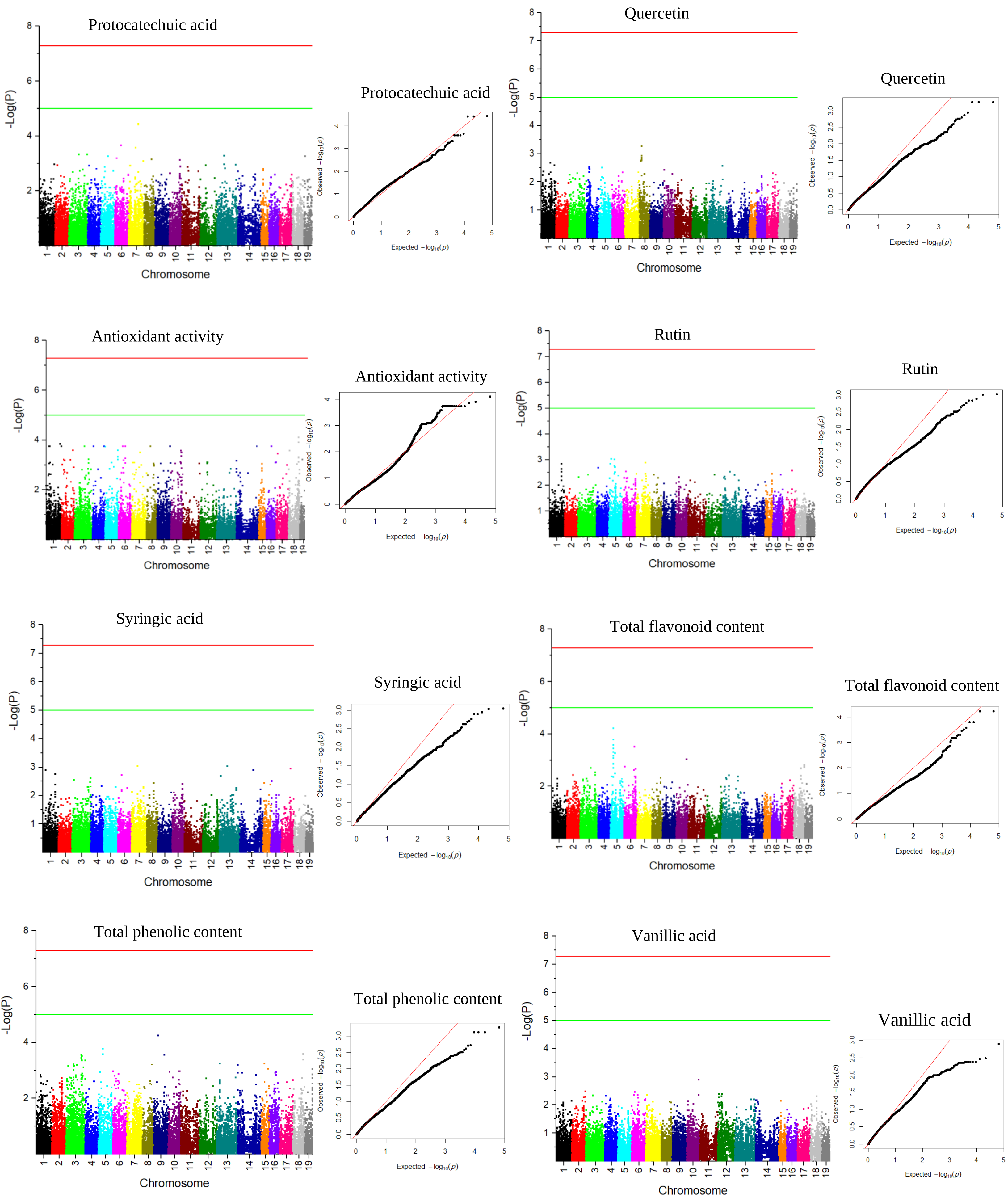
Protocatechuic acid
Quercetin
Antioxidant activity
Rutin
Syringic acid
Total flavonoid content
Total phenolic content
Vanillic acid
-Log(P)
Chromosome
1 2 3 4 5 6 7 8 9 10 11 12 13 14 15 16 17 18 19
Observed −log10(p)
Expected −log10(p)

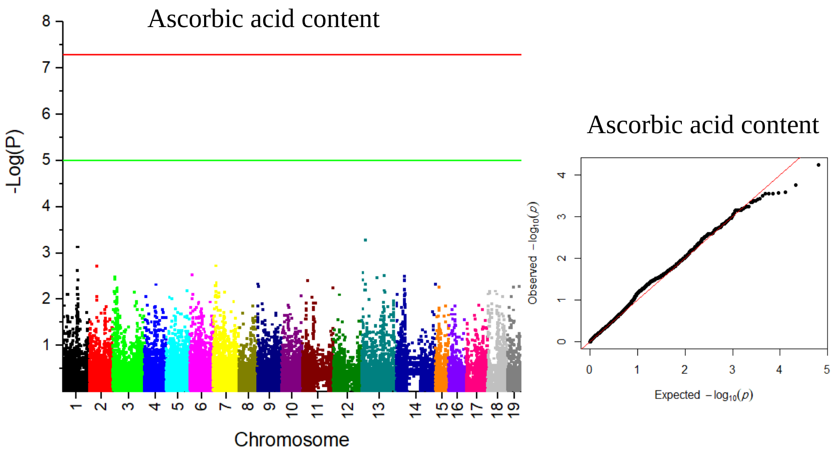

Supplementary Fig. S4. Manhattan plot depicting the genome-wide significant associations between SNP markers and phytochemical traits and phenolic compounds under well-watered condition. The corresponding quantile-quantile (QQ) plot is shown at the right side of the Manhattan plot. Markers on different chromosomes are denoted by different colors.
