# Supplementary material for: Integration of genome-wide association studies, metabolomics, and transcriptomics reveals phenolic acid- and flavonoid-associated genes and their regulatory elements under drought stress in rapeseed flowers
Source: Front Plant Sci. 2024 Jan 11;14:1249142. doi: 10.3389/fpls.2023.1249142 (PMC10808681; doi:10.3389/fpls.2023.1249142)
Supplement: Supplementary file 5 [file DataSheet_5.pdf]

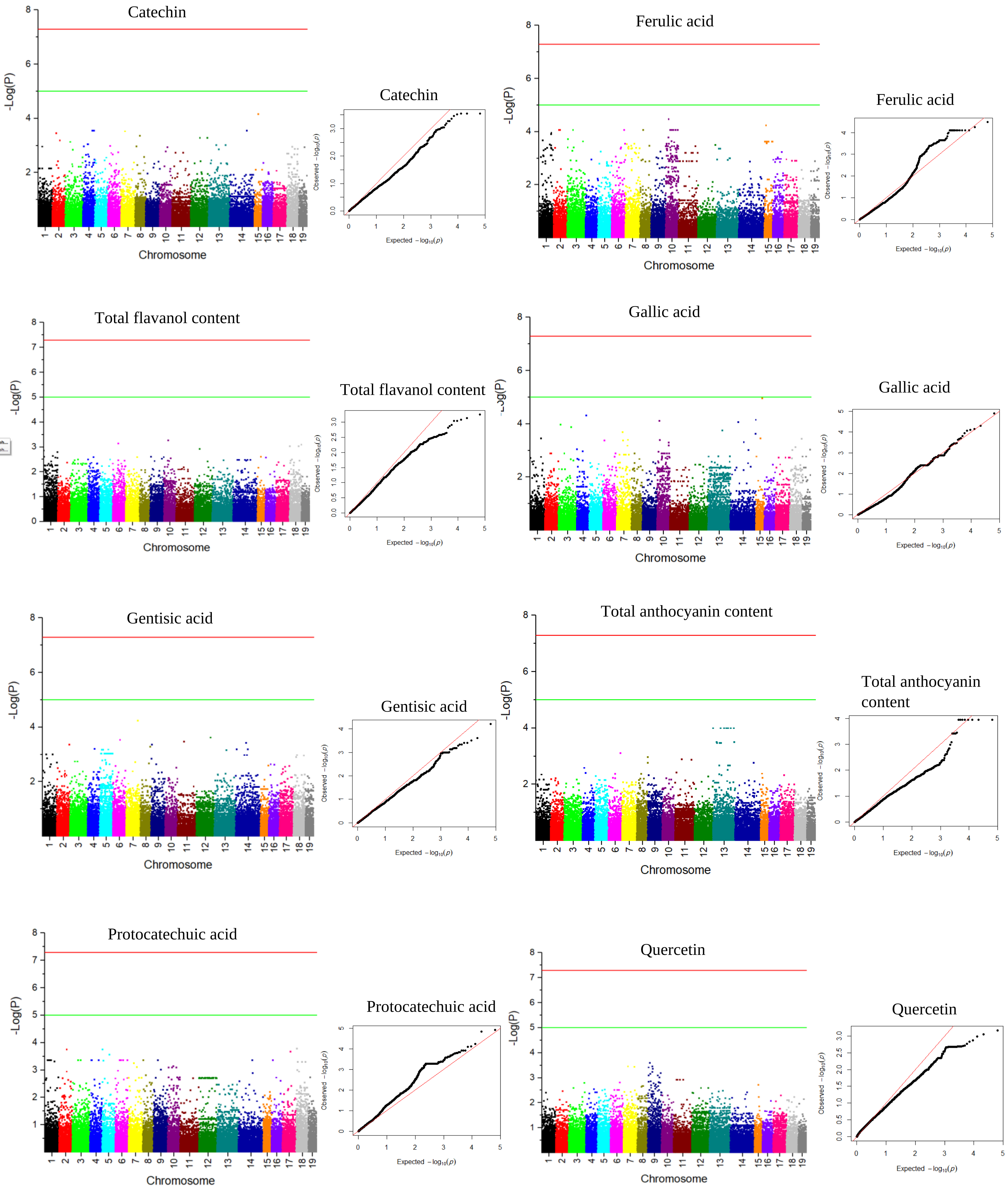

Catechin
-Log(P)
Chromosome
Catechin
Observed −log10(p)
Expected −log10(p)
Ferulic acid
-Log(P)
Chromosome
Ferulic acid
Observed −log10(p)
Expected −log10(p)
Total flavanol content
-Log(P)
Chromosome
Total flavanol content
Observed −log10(p)
Expected −log10(p)
Gallic acid
-Log(P)
Chromosome
Gallic acid
Observed −log10(p)
Expected −log10(p)
Gentisic acid
-Log(P)
Chromosome
Gentisic acid
Observed −log10(p)
Expected −log10(p)
Total anthocyanin content
-Log(P)
Chromosome
Total anthocyanin content
Observed −log10(p)
Expected −log10(p)
Protocatechuic acid
-Log(P)
Chromosome
Protocatechuic acid
Observed −log10(p)
Expected −log10(p)
Quercetin
-Log(P)
Chromosome
Quercetin
Observed −log10(p)
Expected −log10(p)

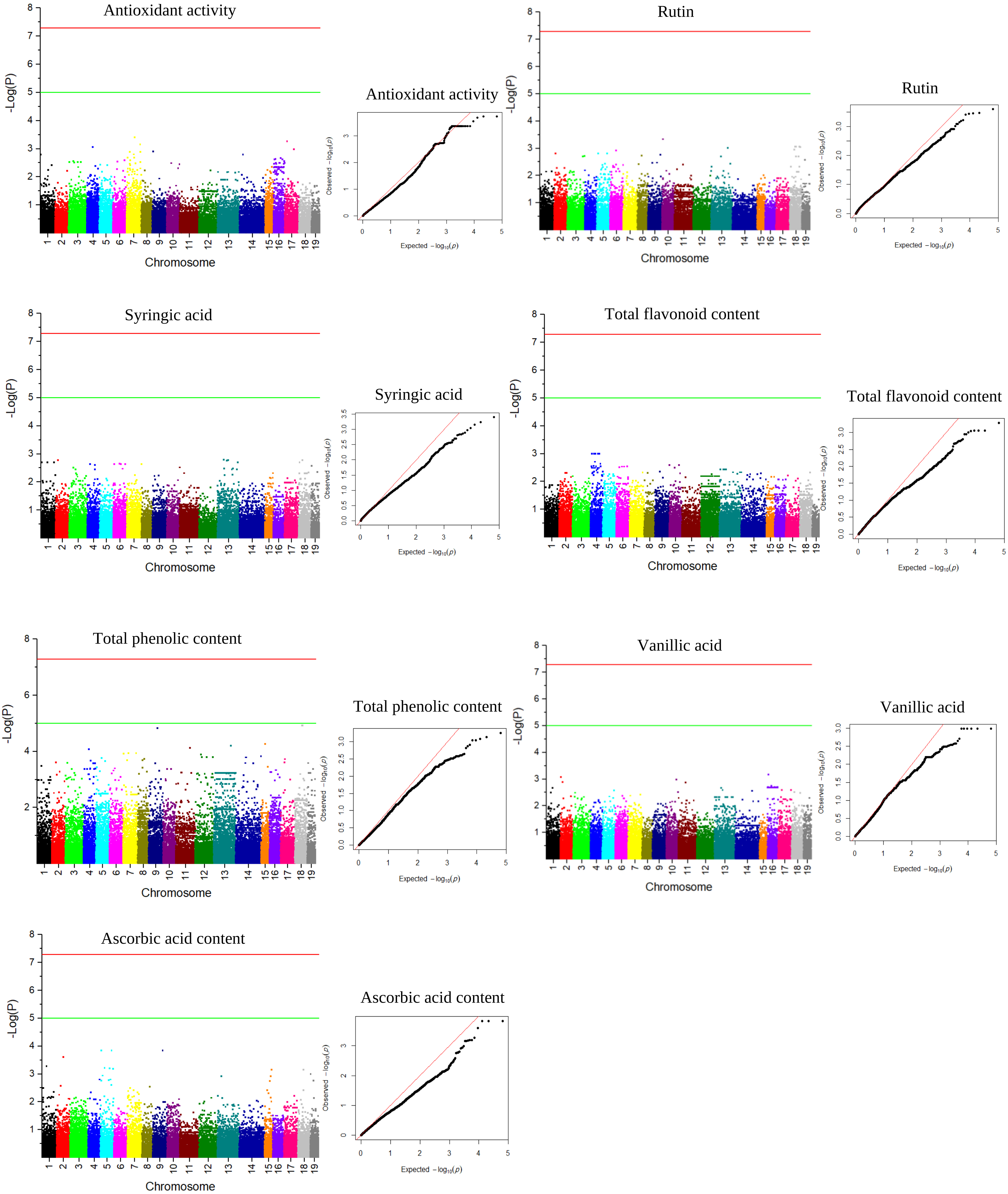

Antioxidant activity
Rutin
Syringic acid
Total flavonoid content
Total phenolic content
Vanillic acid
Ascorbic acid content
-Log(P)
Chromosome
Observed −log10(p)
Expected −log10(p)

Supplementary Fig. S5. Manhattan plot depicting the genome-wide significant associations between SNP markers and phytochemical traits and phenolic compounds under drought stress condition. The corresponding quantile-quantile (QQ) plot is shown at the right side of the Manhattan plot. Markers on different chromosomes are denoted by different colors.
