# Supplementary material for: Integration of genome-wide association studies, metabolomics, and transcriptomics reveals phenolic acid- and flavonoid-associated genes and their regulatory elements under drought stress in rapeseed flowers
Source: Front Plant Sci. 2024 Jan 11;14:1249142. doi: 10.3389/fpls.2023.1249142 (PMC10808681; doi:10.3389/fpls.2023.1249142)
Supplement: Supplementary file 6 [file DataSheet_6.pdf]

Supplementary Table S1. Description of rapeseed (*Brassica napus*) accessions tested for phytochemical traits, phenolic compounds and genome-wide association study (GWAS) for traits at well-watered and drought-stressed.

Code	Accession	Origin	Breeding status	Seasonal type[a]	Pollination status	Code	Accession	Origin	Breeding status	Seasonal type	Pollination status
G01	Okapi	France	Cultivar	Winter	Open pollinating	G31	Es neptune	France	Hybrid	Winter	-
G02	Ahmadi	Iran	Cultivar	Winter	Open pollinating	G32	Kodiac	Germany	Cultivar	Winter	Open pollinating
G03	Slm046	France	Breeding line	Winter	Open pollinating	G33	Tassilo	Germany	Hybrid	Winter	-
G04	Sw102	Iran	Breeding line	Winter	Open pollinating	G34	Triangle	Germany	Hybrid	Winter	-
G05	l72	Iran	Breeding line	Winter	Open pollinating	G35	Traviata	Germany	Hybrid	Winter	-
G06	963	Iran	Breeding line	Winter	Open pollinating	G36	Diffusion	Germany	Cultivar	Winter	Open pollinating
G07	957	Iran	Breeding line	Winter	Open pollinating	G37	Zorica	Serbia	Cultivar	Winter	Open pollinating
G08	Expower	America	Hybrid	Winter	-	G38	Zelatna	Serbia	Cultivar	Winter	Open pollinating
G09	Dk extorm	America	Hybrid	Winter	-	G39	Es Hydromel	France	Hybrid	Winter	-
G10	Dk excalibur	America	Hybrid	Winter	-	G40	Es Alonso	France	Hybrid	Winter	-
G11	Es vatalie	France	Hybrid	Winter	-	G41	Es Darko	France	Hybrid	Winter	-
G12	Es Danube	France	Hybrid	Winter	-	G42	Es lauven	France	Hybrid	Winter	-
G13	Es Artist	France	Hybrid	Winter	-	G43	Es mercure	France	Hybrid	Winter	-
G14	Sy vesuvio	America	Hybrid	Winter	-	G44	Okapi-original-mashkad	France	Hybrid	Winter	-
G15	Sy melea	America	Hybrid	Winter	-	G45	Garou	Germany	Cultivar	Winter	-
G16	Sy Harnus	America	Hybrid	Winter	-	G46	Rohan	Germany	Cultivar	Winter	Open pollinating
G17	1110	Iran	Breeding line	Winter	Open pollinating	G47	Gk Reka	Hungary	Cultivar	Winter	Open pollinating
G18	1112	Iran	Breeding line	Winter	Open pollinating	G48	Gk csenge	Hungary	Cultivar	Winter	Open pollinating
G19	1093	Iran	Breeding line	Winter	Open pollinating	G49	Z-800-3	Iran	Breeding line	Winter	Open pollinating
G20	1096	Iran	Breeding line	Winter	Open pollinating	G50	Z-800-6	Iran	Breeding line	Winter	Open pollinating
G21	1114	Iran	Breeding line	Winter	Open pollinating	G51	Z-900-3	Iran	Breeding line	Winter	Open pollinating
G22	1204	Iran	Breeding line	Winter	Open pollinating	G52	Z-900-6	Iran	Breeding line	Winter	Open pollinating
G23	1206	Iran	Breeding line	Winter	Open pollinating	G53	Z-900-7	Iran	Breeding line	Winter	Open pollinating
G24	Talaye	Germany	Cultivar	Winter	Open pollinating	G54	Z-900-8	Iran	Breeding line	Winter	Open pollinating
G25	Opera	Germany	Cultivar	Winter	Open pollinating	G55	Z-900-9	Iran	Breeding line	Winter	Open pollinating
G26	Licorol	Germany	Cultivar	Winter	Open pollinating	G56	Z-900-10	Iran	Breeding line	Winter	Open pollinating
G27	Modena	Russia	Cultivar	Winter	Open pollinating	G57	T-800-1	Iran	Breeding line	Winter	Open pollinating
G28	Zarfam	Iran	Cultivar	Faculative	Open pollinating	G58	T-800-6	Iran	Breeding line	Winter	Open pollinating
G29	Gabriella	Hungary	Cultivar	Winter	Open pollinating	G59	Cod 769	Iran	Breeding line	Winter	Open pollinating
G30	Brutus	Germany	Hybrid	Winter	-	G60	Exp-800-3	Iran	Breeding line	Winter	Open pollinating

Supplementary Table S1. (Continued)

Code	Accession	Origin	Breeding status	Seasonal type	Pollination status
G61	Exp-900-1	Iran	Breeding line	Winter	Open pollinating
G62	Code 12	Iran	Breeding line	Winter	Open pollinating
G63	Code 21	Iran	Breeding line	Winter	Open pollinating
G64	Code 20	Iran	Breeding line	Winter	Open pollinating
G65	Code 18	Iran	Breeding line	Winter	Open pollinating
G66	Code 255	Iran	Breeding line	Winter	Open pollinating
G67	Code 262	Iran	Breeding line	Winter	Open pollinating
G68	Code 763	Iran	Breeding line	Winter	Open pollinating
G69	Code 768	Iran	Breeding line	Winter	Open pollinating
G70	Code 751	Iran	Breeding line	Winter	Open pollinating
G71	Code 268	Iran	Breeding line	Winter	Open pollinating
G72	Code 270	Iran	Breeding line	Winter	Open pollinating
G73	Code 655	Iran	Breeding line	Winter	Open pollinating
G74	Code 914	Iran	Breeding line	Winter	Open pollinating
G75	Code 253	Iran	Breeding line	Winter	Open pollinating
G76	Code 266	Iran	Breeding line	Winter	Open pollinating
G77	Code 263	Iran	Breeding line	Winter	Open pollinating
G78	Code 770	Iran	Breeding line	Winter	Open pollinating
G79	Code 264	Iran	Breeding line	Winter	Open pollinating
G80	Code 901	Iran	Breeding line	Winter	Open pollinating
G81	Code 23	Iran	Breeding line	Winter	Open pollinating
G82	Code 655	Iran	Breeding line	Winter	Open pollinating
G83	Code 1	Iran	Breeding line	Winter	Open pollinating
G84	Code 3	Iran	Breeding line	Winter	Open pollinating
G85	Code 11	Iran	Breeding line	Winter	Open pollinating
G86	Code 19	Iran	Breeding line	Winter	Open pollinating
G87	Cooper	Germany	Breeding line	Winter	Open pollinating
G88	Adriana	Germany	Breeding line	Winter	Open pollinating
G89	Code 767	Iran	Breeding line	Winter	Open pollinating
G90	Code 752	Iran	Breeding line	Winter	Open pollinating
G91	B20-p15 R13 S2-13	Iran	Breeding line	Winter	Open pollinating
G92	B17-p12 R1 S2-1	Iran	Breeding line	Winter	Open pollinating
G93	B17-p12 R9 S2-1	Iran	Breeding line	Winter	Open pollinating
G94	B19-p14 R12 S2-1	Iran	Breeding line	Winter	Open pollinating
G95	B20-p15 R14 S2-3	Iran	Breeding line	Winter	Open pollinating
G96	B17-p12 R2 S2-1	Iran	Breeding line	Winter	Open pollinating
G97	B19-p14 R8 S2-1	Iran	Breeding line	Winter	Open pollinating
G98	B15-p11 R15 S2-5	Iran	Breeding line	Winter	Open pollinating
G99	B17-p12 R3 S2-1	Iran	Breeding line	Winter	Open pollinating
G100	B12-p9 R2 S2-3	Iran	Breeding line	Winter	Open pollinating
G101	B19-p14 R1 S2-2	Iran	Breeding line	Winter	Open pollinating
G102	B19-p14 R1 S2-1	Iran	Breeding line	Winter	Open pollinating
G103	B16-p11 R16 S2-1	Iran	Breeding line	Winter	Open pollinating
G104	B19-p14 R12 S2-6	Iran	Breeding line	Winter	Open pollinating
G105	B12-p9 R1 S2-1	Iran	Breeding line	Winter	Open pollinating
G106	B17-p12 R13 S2-1	Iran	Breeding line	Winter	Open pollinating
G107	B17 okapi -1	Iran	Breeding line	Winter	Open pollinating
G108	B17-p15 R13 S2-8	Iran	Breeding line	Winter	Open pollinating
G109	Code-3-2	Iran	Breeding line	Winter	Open pollinating
G110	H4815	Australia	Cultivar	Spring	Open pollinating
G111	Dalgan	Iran	Cultivar	Spring	Open pollinating
G112	Licord	Germany	Cultivar	Winter	Open pollinating
G113	H308	Australia	Cultivar	Spring	Open pollinating
G114	Zafar	Iran	Cultivar	Spring	Open pollinating
G115	Sarigol	Iran	Cultivar	Spring	Open pollinating
G116	H401	Australia	Cultivar	Spring	Open pollinating
G117	Karaj1	Iran	Cultivar	Winter	Open pollinating
G118	RGS	Germany	Cultivar	Spring	Open pollinating
G119	H420	Australia	Cultivar	Spring	Open pollinating

[a]Seasonal types were distinguished by their flowering times at Research Farm of Plant Production and Genetics, Shiraz, Iran.
