# Supplementary material for: Integration of genome-wide association studies, metabolomics, and transcriptomics reveals phenolic acid- and flavonoid-associated genes and their regulatory elements under drought stress in rapeseed flowers
Source: Front Plant Sci. 2024 Jan 11;14:1249142. doi: 10.3389/fpls.2023.1249142 (PMC10808681; doi:10.3389/fpls.2023.1249142)
Supplement: Supplementary file 7 [file DataSheet_7.pdf]

Supplementary Table S2. Concentrations of macronutrient and micronutrient elements in soil of experiment in 2020.

Element	Symbol	Value
**Macronutrients**		
Calcium (Mgk^{-1})	Ca	4511
Phosphorus (Mgk^{-1})	P	34.59
Potassium (Mgk^{-1})	K	1140
Nitrogen (%)	N	0.23
**Micronutrients**		
Manganese (Mgk^{-1})	Mn	21.83
Zinc (Mgk^{-1})	Zn	2.69
Iron (Mgk^{-1})	Fe	5.70
Boron (ppm)	B	0.36
**pH**		7.20

The concentrations of elements are in mg/kg, parts per million (ppm), and Percent (%).
