# Supplementary material for: Integration of genome-wide association studies, metabolomics, and transcriptomics reveals phenolic acid- and flavonoid-associated genes and their regulatory elements under drought stress in rapeseed flowers
Source: Front Plant Sci. 2024 Jan 11;14:1249142. doi: 10.3389/fpls.2023.1249142 (PMC10808681; doi:10.3389/fpls.2023.1249142)
Supplement: Supplementary file 8 [file DataSheet_8.pdf]

Supplementary Table S3. Results of ANOVA on the effects of genotype and irrigation regime on the phytochemical traits, antioxidant activity and phenolic compounds rapeseed (*Brassica napus*).

	Genotype	Environment	Genotype × Environment
df	118	1	118
Total phenolic content	833.97**	3876.14**	14.53**
Total flavonoid content	290573.85**	1424841.42**	13603.45**
Total flavanol content	1185954.40**	2960218.30**	44060.30**
Antioxidant activity	2.20**	59.25**	0.15 ns
Ascorbic acid content	316.36**	3689.14**	37.45 ns
Total anthocyanin content	18769.68**	390058.79**	2095.44 ns
Gallic acid	1.99**	45.90**	0.17**
Protocatechuic acid	12.67**	141.02**	3.92**
Catechin	1.30**	434.90**	0.64**
Vanillic acid	6.72**	14.42**	0.63**
Epicatechin	97.38 **	2376.76**	65.64**
Syringic acid	782.74**	722.75**	39.82**
Chlorogenic acid	9.22**	71.58**	4.40 ns
Gentisic acid	4.79**	0.26 ns	0.66 ns
Caffeic acid	79.83**	3340.48**	74.43**
Coumaric acid	9.58**	135.82**	6.45 ns
Ferulic acid	1.27**	48.18**	0.64**
Rutin	35.16 **	334.08**	9.44**
Myricetin	3.03**	496.02**	1.28**
Quercetin	0.10**	0.02 ns	0.08 **

**: $P < 0.01$, ns: non- significant
