# Supplementary material for: Integration of genome-wide association studies, metabolomics, and transcriptomics reveals phenolic acid- and flavonoid-associated genes and their regulatory elements under drought stress in rapeseed flowers
Source: Front Plant Sci. 2024 Jan 11;14:1249142. doi: 10.3389/fpls.2023.1249142 (PMC10808681; doi:10.3389/fpls.2023.1249142)
Supplement: Supplementary file 9 [file DataSheet_9.pdf]

Supplementary Table S4. Eigen vectors and eigen values of the principal components (PC) for various phytochemical traits, phenolic compounds, and antioxidant activity in rapeseed (*Brassica napus*) accessions at well-watered condition.

Variable	PC1	PC2	PC3	PC4	PC5	PC6	PC7
Eigenvalue	3.64	2.87	2.33	2.13	1.37	1.19	1.03
Variation (%)	17.9	12.3	11.68	10.69	6.88	5.96	5.18
Cumulative	17.9	30.2	41.88	52.57	59.45	65.41	70.59
Total phenolic content	0.447	-0.097	-0.078	-0.021	-0.130	0.046	-0.030
Total flavonoid content	0.056	-0.283	0.290	0.099	-0.158	0.280	-0.084
Total flavanol content	-0.032	-0.242	0.011	0.198	0.083	0.559	0.012
Antioxidant activity	-0.045	0.267	-0.164	-0.306	-0.175	-0.080	0.452
Ascorbic acid content	0.158	-0.113	0.067	0.141	0.355	-0.123	0.168
Total anthocyanin content	0.142	-0.183	-0.065	0.084	0.416	-0.289	-0.345
Gallic acid	0.419	-0.001	-0.180	-0.011	-0.156	0.027	0.149
Protocatechuic acid	0.380	0.110	-0.075	-0.006	0.052	0.059	-0.282
Catechin	0.494	0.015	-0.070	0.078	-0.025	0.031	0.065
Vanillic acid	0.233	-0.270	-0.153	-0.201	-0.136	0.152	0.324
Epicatechin	0.158	-0.242	0.373	-0.221	0.067	-0.093	0.059
Syringic acid	0.088	0.460	0.016	0.230	0.167	0.252	0.135
Chlorogenic acid	0.227	0.194	0.417	-0.079	0.143	0.121	0.091
Gentisic acid	0.040	0.169	0.528	-0.083	-0.207	0.000	-0.059
Caffeic acid	0.003	0.030	0.414	-0.229	-0.045	-0.095	0.059
Coumaric acid	-0.006	-0.232	0.190	0.435	0.119	-0.167	0.285
Ferulic acid	-0.020	-0.018	0.065	0.521	-0.184	-0.249	0.411
Rutin	0.028	0.434	0.043	0.183	0.248	0.282	-0.004
Myricetin	0.057	0.034	-0.011	-0.292	0.543	-0.169	0.289
Quercetin	0.200	0.261	0.045	0.190	-0.270	-0.425	-0.242
