# Supplementary material for: Integration of genome-wide association studies, metabolomics, and transcriptomics reveals phenolic acid- and flavonoid-associated genes and their regulatory elements under drought stress in rapeseed flowers
Source: Front Plant Sci. 2024 Jan 11;14:1249142. doi: 10.3389/fpls.2023.1249142 (PMC10808681; doi:10.3389/fpls.2023.1249142)
Supplement: Supplementary file 10 [file DataSheet_10.pdf]

Supplementary Table S5. Eigen vectors and eigen values of the principal components (PC) for various phytochemical traits, phenolic compounds, and antioxidant activity in rapeseed (*Brassica napus*) accessions under drought stress.

Variable	PC1	PC2	PC3	PC4	PC5	PC6	PC7	PC8
Eigenvalue	3.2	2.36	1.97	1.74	1.45	1.38	1.1	1.02
Variation (%)	13.1	10.6	8.85	8.71	7.27	6.91	5.55	5.14
Cumulative	13.1	23.7	32.55	41.26	48.53	55.44	60.99	66.13
Total phenolic content	0.128	0.326	0.272	-0.352	-0.228	-0.218	0.086	-0.074
Total flavonoid content	-0.078	0.105	-0.145	-0.316	0.081	0.394	-0.248	-0.309
Total flavanol content	0.121	0.077	-0.212	-0.214	0.481	0.089	0.025	0.437
Antioxidant activity	-0.166	0.078	0.456	0.046	-0.066	-0.070	-0.264	-0.099
Ascorbic acid content	0.040	0.029	-0.110	-0.252	-0.275	0.431	-0.174	-0.170
Total anthocyanin content	-0.017	0.227	-0.063	0.322	-0.284	0.361	0.078	0.032
Gallic acid	0.296	0.343	0.241	-0.150	-0.201	0.025	0.012	0.201
Protocatechuic acid	0.375	0.024	0.044	0.109	0.275	0.128	-0.155	-0.268
Catechin	0.435	0.068	0.009	-0.145	-0.136	0.145	0.122	-0.049
Vanillic acid	0.088	0.506	-0.040	-0.059	0.207	-0.055	0.179	0.209
Epicatechin	0.400	-0.070	0.082	0.258	0.158	0.015	-0.021	-0.375
Syringic acid	-0.011	-0.274	0.418	-0.271	0.079	-0.067	0.298	-0.021
Chlorogenic acid	-0.017	-0.230	0.146	0.115	-0.209	0.283	-0.292	0.529
Gentisic acid	-0.281	-0.093	0.304	-0.035	0.066	0.220	0.247	-0.084
Caffeic acid	0.327	-0.125	0.170	0.190	-0.197	0.170	0.121	0.239
Coumaric acid	-0.107	0.239	0.201	0.223	0.403	0.270	-0.088	0.077
Ferulic acid	0.175	-0.201	-0.360	0.093	-0.163	-0.064	0.369	-0.009
Rutin	0.237	-0.334	0.256	-0.050	0.253	0.194	0.115	0.033
Myricetin	-0.226	0.231	0.061	0.271	-0.006	0.256	0.537	-0.131
Quercetin	0.111	0.144	0.106	0.422	-0.023	-0.291	-0.239	-0.026
