# Supplementary material for: Integration of genome-wide association studies, metabolomics, and transcriptomics reveals phenolic acid- and flavonoid-associated genes and their regulatory elements under drought stress in rapeseed flowers
Source: Front Plant Sci. 2024 Jan 11;14:1249142. doi: 10.3389/fpls.2023.1249142 (PMC10808681; doi:10.3389/fpls.2023.1249142)
Supplement: Supplementary file 11 [file DataSheet_11.pdf]

Supplementary Table S6. Stepwise regression for determining the most important phenolics relative to antioxidant activity at well-watered and drought stress conditions.

Variable	Parameter Estimate	Standard Error	Type II SS	F Value	*Pr* > F
**Well-watered condition**					
Intercept	92.68131	0.42108	28346	48445.3	<.0001
Chlorogenic acid	0.10945	0.04404	3.61375	6.18	0.0144
Total flavonoid content	-0.00058186	0.00024584	3.27770	5.60	0.0196
Total flavonol content	-0.00034039	0.00011916	4.77416	8.16	0.0051
Total anthocyanin content	-0.00229	0.00092565	3.59185	6.14	0.0147
**Drought stress condition**					
Intercept	92.18684	0.49077	14571	35283.8	<.0001
Gentisic acid	0.10723	0.04707	2.10405	5.19	0.0246
Coumaric acid	0.04733	0.02372	1.61458	3.98	0.0383
Myricetin	0.07549	0.04238	1.28629	3.17	0.0275
Ferulic acid	-0.12048	0.06542	1.37488	3.39	0.0281
Total flavonol content	-0.0003658	0.00012045	3.74042	9.23	0.0030
