# Supplementary material for: Integration of genome-wide association studies, metabolomics, and transcriptomics reveals phenolic acid- and flavonoid-associated genes and their regulatory elements under drought stress in rapeseed flowers
Source: Front Plant Sci. 2024 Jan 11;14:1249142. doi: 10.3389/fpls.2023.1249142 (PMC10808681; doi:10.3389/fpls.2023.1249142)
Supplement: Supplementary file 12 [file DataSheet_12.docx]

| Supplementary Table S7. Significantly associated SNP markers with phenolic compounds at *P* < 0.00001 analyzed through MLM based statistical model of 119 rapeseed (*Brassica napus*) accessions under well-watered condition. | | | | | | | | | | | |
| --- | --- | --- | --- | --- | --- | --- | --- | --- | --- | --- | --- |
| Phenolic compounds | SNP marker | Linkage group | Position (bp) | *P*-value | *R^2^* (%) | Metabolite | SNP marker | Linkage group | Position (bp) | *P*-value | *R^2^* (%) |
| Gallic acid | Bn-A10-p1655784 | A10 | 16316355 | 3.63E-06 | 0.29245 | Caffeic acid | Bn-A07-p13650653 | A07 | 15643197 | 8.63E-07 | 0.29731 |
|  | Bn-scaff_21925_1-p201015 | C04 | 1857195 | 3.36E-06 | 0.2943 |  | Bn-A07-p19880968 | A07 | 21698267 | 8.91E-06 | 0.27473 |
|  | Bn-scaff_16864_1-p22602 | C04 | 1891703 | 2.54E-06 | 0.30126 |  | Bn-A07-p20511320 | A07 | 22280456 | 3.65E-06 | 0.2969 |
| Epicatechin | Bn-A01-p24950985 | A01 | 249937 | 1.28E-06 | 0.2422 |  | Bn-A08-p5806240 | A08 | 281382 | 6.83E-07 | 0.30301 |
|  | Bn-A01-p1158564 | A01 | 762276 | 7.64E-06 | 0.2425 |  | Bn-A08-p8374080 | A08 | 7231645 | 4.73E-06 | 0.2904 |
|  | Bn-A05-p21973951 | A05 | 20109429 | 7.36E-06 | 0.24335 |  | Bn-A09-p28759144 | A09 | 253552 | 3.53E-06 | 0.29772 |
|  | Bn-A07-p21631043 | A07 | 23173894 | 7.00E-06 | 0.24448 |  | Bn-A09-p28759763 | A09 | 253555 | 4.68E-06 | 0.2907 |
|  | Bn-A08-p7497342 | A08 | 6504460 | 1.28E-06 | 0.2422 |  | Bn-A09-p33358298 | A09 | 365574 | 3.44E-06 | 0.29836 |
|  | Bn-A08-p4869575 | A08 | 12865213 | 1.28E-06 | 0.2422 |  | Bn-A09-p33406899 | A09 | 370396 | 3.04E-06 | 0.30147 |
|  | Bn-A09-p27678823 | A09 | 25596369 | 2.46E-06 | 0.30305 |  | Bn-A09-p28758854 | A09 | 26690641 | 4.68E-06 | 0.2907 |
|  | Bn-A10-p3980498 | A10 | 873182 | 6.29E-06 | 0.24695 |  | Bn-A09-p30371768 | A09 | 28142634 | 2.77E-06 | 0.30388 |
|  | Bn-A10-p2764779 | A10 | 1167496 | 1.28E-06 | 0.2422 |  | Bn-A09-p33271433 | A09 | 30953917 | 3.42E-06 | 0.29851 |
|  | Bn-A10-p11423143 | A10 | 4434401 | 1.93E-07 | 0.33018 |  | Bn-scaff_16445_1-p1679519 | A09 | 31043889 | 2.33E-06 | 0.30815 |
|  | Bn-A10-p13309178 | A10 | 13354247 | 5.56E-06 | 0.24983 |  | Bn-A09-p35981539 | A09 | 33104863 | 1.81E-07 | 0.29001 |
|  | Bn-A10-p12842513 | A10 | 43008308 | 3.26E-06 | 0.26222 |  | Bn-A09-p35981493 | A09 | 37842484 | 1.81E-07 | 0.29001 |
|  | Bn-scaff_21778_1-p269753 | C03 | 5063952 | 6.46E-06 | 0.27924 |  | Bn-A09-p35867904 | A09 | 37959234 | 1.81E-07 | 0.29001 |
|  | Bn-scaff_22148_1-p157602 | C04 | 29181029 | 9.34E-06 | 0.27023 |  | Bn-A10-p8544988 | A10 | 180322 | 2.81E-06 | 0.30351 |
|  | Bn-scaff_16268_1-p543187 | C05 | 162937 | 6.99E-06 | 0.24453 |  | Bn-A10-p11783750 | A10 | 2343226 | 1.08E-06 | 0.29196 |
|  | Bn-scaff_16268_1-p548269 | C05 | 163550 | 6.99E-06 | 0.24453 |  | Bn-A10-p12066927 | A10 | 2728229 | 3.64E-06 | 0.29699 |
|  | Bn-scaff_16268_1-p548337 | C05 | 163557 | 6.99E-06 | 0.24453 |  | Bn-A10-p12067020 | A10 | 2728463 | 3.64E-06 | 0.29699 |
|  | Bn-scaff_16268_1-p533546 | C05 | 12718531 | 6.99E-06 | 0.24453 |  | Bn-A10-p12067206 | A10 | 2728556 | 3.64E-06 | 0.29699 |
|  | Bn-scaff_16268_1-p532339 | C05 | 12719736 | 6.99E-06 | 0.24453 |  | Bn-A10-p12231523 | A10 | 2910403 | 2.75E-06 | 0.30402 |
|  | Bn-scaff_16268_1-p507110 | C05 | 12740819 | 6.99E-06 | 0.24453 |  | Bn-A10-p12308946 | A10 | 2987256 | 4.43E-06 | 0.29207 |
|  | Bn-scaff_18589_1-p127443 | C05 | 29638309 | 6.35E-06 | 0.24675 |  | Bn-A10-p12311753 | A10 | 2989088 | 4.43E-06 | 0.29206 |
|  | Bn-scaff_18589_1-p126670 | C05 | 29639083 | 6.35E-06 | 0.24675 |  | Bn-A10-p12311817 | A10 | 2995832 | 4.43E-06 | 0.29207 |
|  | Bn-scaff_18589_1-p94879 | C05 | 29664439 | 6.35E-06 | 0.24675 |  | Bn-A10-p12313235 | A10 | 2995897 | 4.43E-06 | 0.29206 |
|  | Bn-scaff_18589_1-p76811 | C05 | 29685678 | 6.35E-06 | 0.24675 |  | Bn-A10-p12315853 | A10 | 2997675 | 4.43E-06 | 0.29207 |
|  | Bn-scaff_19224_1-p29613 | C05 | 29768943 | 6.35E-06 | 0.24675 |  | Bn-A10-p12315948 | A10 | 2999883 | 4.43E-06 | 0.29207 |
|  | Bn-scaff_19224_1-p29858 | C05 | 29769188 | 6.35E-06 | 0.24675 |  | Bn-A10-p12316390 | A10 | 2999978 | 4.43E-06 | 0.29207 |
|  | Bn-scaff_16110_1-p2336219 | C07 | 42536740 | 1.28E-06 | 0.2422 |  | Bn-A10-p12316420 | A10 | 3000418 | 4.43E-06 | 0.29207 |
| Caffeic acid | Bn-scaff_15808_1-p529075 | A01 | 179675 | 2.52E-06 | 0.30618 |  | Bn-A10-p12316487 | A10 | 3000448 | 4.43E-06 | 0.29207 |
|  | Bn-scaff_22481_1-p143945 | A01 | 17259349 | 3.49E-06 | 0.29803 |  | Bn-A10-p12316900 | A10 | 3000902 | 4.43E-06 | 0.29207 |
|  | Bn-A02-p1402685 | A02 | 22913 | 1.81E-07 | 0.29001 |  | Bn-A10-p12317263 | A10 | 3001078 | 4.43E-06 | 0.29206 |
|  | Bn-A02-p1264906 | A02 | 114981 | 1.11E-06 | 0.29123 |  | Bn-A10-p7252424 | A10 | 8893827 | 4.63E-06 | 0.29096 |
|  | Bn-A02-p24237359 | A02 | 124901 | 4.11E-06 | 0.29396 |  | Bn-A10-p8867175 | A10 | 10257219 | 4.77E-06 | 0.29022 |
|  | Bn-A02-p1485915 | A02 | 167480 | 2.78E-06 | 0.30373 |  | Bn-A10-p8984096 | A10 | 38450125 | 1.02E-06 | 0.29323 |
|  | Bn-A02-p1517113 | A02 | 200031 | 4.60E-06 | 0.29113 |  | Bn-scaff_15712_13-p61367 | C02 | 254584 | 1.93E-06 | 0.31296 |
|  | Bn-A02-p2851231 | A02 | 393886 | 4.13E-07 | 0.31533 |  | Bn-scaff_20836_1-p30574 | C02 | 294444 | 1.81E-07 | 0.29001 |
|  | Bn-A02-p27450764 | A02 | 426966 | 1.49E-06 | 0.31956 |  | Bn-scaff_15712_13-p38138 | C02 | 372866 | 2.18E-06 | 0.30992 |
|  | Bn-A02-p27755843 | A02 | 444005 | 4.25E-06 | 0.29308 |  | Bn-scaff_17623_1-p837616 | C02 | 455566 | 3.54E-06 | 0.29765 |
|  | Bn-A02-p27399448 | A02 | 544396 | 2.62E-06 | 0.30519 |  | Bn-scaff_29061_1-p30884 | C02 | 4292775 | 3.55E-06 | 0.29761 |
|  | Bn-A02-p27490396 | A02 | 554076 | 1.17E-06 | 0.29002 |  | Bn-scaff_17522_1-p1497074 | C02 | 5162738 | 3.01E-06 | 0.30175 |
|  | Bn-A02-p27503846 | A02 | 555293 | 2.45E-06 | 0.30691 |  | Bn-scaff_17522_1-p1483337 | C02 | 5172871 | 2.53E-06 | 0.3061 |
|  | Bn-A02-p27514988 | A02 | 556962 | 8.62E-07 | 0.29736 |  | Bn-scaff_20942_1-p734832 | C02 | 10513865 | 2.06E-06 | 0.31128 |
|  | Bn-A02-p27525531 | A02 | 558217 | 8.62E-07 | 0.29736 |  | Bn-scaff_20942_1-p718900 | C02 | 10523601 | 5.34E-07 | 0.30903 |
|  | Bn-A02-p27565132 | A02 | 563638 | 3.58E-06 | 0.29738 |  | Bn-scaff_17109_1-p675773 | C02 | 41646489 | 2.99E-06 | 0.3019 |
|  | Bn-A02-p27567204 | A02 | 564577 | 3.38E-06 | 0.29886 |  | Bn-scaff_17109_1-p626573 | C02 | 41712868 | 1.12E-06 | 0.29105 |
|  | Bn-A02-p27567264 | A02 | 564583 | 2.44E-06 | 0.30702 |  | Bn-scaff_17109_1-p599429 | C02 | 41763391 | 2.55E-06 | 0.30592 |
|  | Bn-A02-p27569069 | A02 | 564747 | 8.62E-07 | 0.29736 |  | Bn-scaff_17109_1-p593012 | C02 | 41769808 | 3.20E-06 | 0.30019 |
|  | Bn-A02-p2075762 | A02 | 701380 | 3.53E-06 | 0.29777 |  | Bn-scaff_17109_1-p557566 | C02 | 41808767 | 3.83E-06 | 0.29571 |
|  | Bn-A02-p25079431 | A02 | 23154160 | 3.25E-06 | 0.29985 |  | Bn-scaff_17109_1-p557456 | C02 | 41808877 | 3.83E-06 | 0.29571 |
|  | Bn-A02-p25105527 | A02 | 23180627 | 4.62E-06 | 0.29102 |  | Bn-scaff_17109_1-p557169 | C02 | 41809164 | 3.79E-06 | 0.29598 |
|  | Bn-A02-p25109769 | A02 | 23183973 | 4.62E-06 | 0.29102 |  | Bn-scaff_17109_1-p511136 | C02 | 41851099 | 3.83E-06 | 0.29571 |
|  | Bn-A02-p26154897 | A02 | 23778761 | 2.56E-06 | 0.30577 |  | Bn-scaff_17109_1-p508730 | C02 | 41853503 | 3.83E-06 | 0.29571 |
|  | Bn-A02-p27055844 | A02 | 24377661 | 3.26E-06 | 0.29972 |  | Bn-scaff_17109_1-p428616 | C02 | 41917311 | 7.61E-07 | 0.30038 |
|  | Bn-A02-p27055872 | A02 | 24377689 | 3.26E-06 | 0.29972 |  | Bn-scaff_15918_1-p310635 | C02 | 42239959 | 4.65E-06 | 0.29086 |
|  | Bn-A02-p27122935 | A02 | 24446240 | 4.62E-06 | 0.29098 |  | Bn-scaff_15918_1-p312140 | C02 | 42241454 | 4.65E-06 | 0.29086 |
|  | Bn-A02-p27226579 | A02 | 24551863 | 1.13E-06 | 0.29078 |  | Bn-scaff_15918_1-p312733 | C02 | 42242048 | 4.65E-06 | 0.29086 |
|  | Bn-A02-p27286923 | A02 | 24599211 | 3.43E-06 | 0.29844 |  | Bn-scaff_15918_1-p318193 | C02 | 42242813 | 4.13E-06 | 0.29381 |
|  | Bn-A02-p27288011 | A02 | 24605400 | 3.26E-06 | 0.29972 |  | Bn-scaff_16162_1-p79842 | C02 | 42315091 | 2.87E-06 | 0.30298 |
|  | Bn-A02-p27288044 | A02 | 24605433 | 3.43E-06 | 0.29844 |  | Bn-scaff_16162_1-p78008 | C02 | 42316910 | 2.87E-06 | 0.30298 |
|  | Bn-A02-p27288121 | A02 | 24605510 | 2.48E-06 | 0.30657 |  | Bn-scaff_17623_1-p956003 | C02 | 42538477 | 3.54E-06 | 0.29765 |
|  | Bn-A02-p27304105 | A02 | 24611363 | 1.61E-06 | 0.31753 |  | Bn-scaff_17623_1-p895939 | C02 | 42597220 | 3.54E-06 | 0.29765 |
|  | Bn-A02-p27786739 | A02 | 24744845 | 4.55E-06 | 0.29141 |  | Bn-scaff_17623_1-p894398 | C02 | 42598792 | 3.54E-06 | 0.29765 |
|  | Bn-A02-p27792444 | A02 | 24751608 | 4.29E-06 | 0.29287 |  | Bn-scaff_17623_1-p706105 | C02 | 42728575 | 4.40E-06 | 0.29223 |
|  | Bn-A02-p27158239 | A02 | 45504191 | 3.26E-06 | 0.29972 |  | Bn-scaff_17623_1-p696488 | C02 | 42736278 | 3.82E-06 | 0.29575 |
|  | Bn-A02-p27403121 | A02 | 45846359 | 1.13E-06 | 0.29078 |  | Bn-scaff_17623_1-p663832 | C02 | 42763469 | 1.81E-07 | 0.29001 |
|  | Bn-A03-p9124799 | A03 | 728544 | 3.94E-06 | 0.29501 |  | Bn-scaff_17623_1-p662478 | C02 | 42764823 | 3.76E-06 | 0.29616 |
|  | Bn-A03-p14423477 | A03 | 13594169 | 5.66E-06 | 0.28595 |  | Bn-scaff_17623_1-p599063 | C02 | 42823098 | 1.81E-07 | 0.29001 |
|  | Bn-A04-p7349794 | A04 | 376495 | 3.99E-06 | 0.2947 |  | Bn-scaff_17623_1-p546659 | C02 | 42864289 | 1.87E-06 | 0.31374 |
|  | Bn-A04-p8004085 | A04 | 378678 | 8.14E-07 | 0.335 |  | Bn-scaff_17623_1-p254765 | C02 | 43091298 | 4.08E-06 | 0.29409 |
|  | Bn-A04-p15670183 | A04 | 741036 | 1.17E-06 | 0.29005 |  | Bn-scaff_17721_1-p794270 | C02 | 43541890 | 2.29E-06 | 0.30859 |
|  | Bn-A04-p15661972 | A04 | 742428 | 4.15E-06 | 0.29369 |  | Bn-scaff_17721_1-p676383 | C02 | 43657545 | 4.81E-06 | 0.29002 |
|  | Bn-A04-p12411454 | A04 | 3938098 | 1.81E-07 | 0.29001 |  | Bn-scaff_17721_1-p347555 | C02 | 43966422 | 4.02E-06 | 0.29448 |
|  | Bn-A04-p5133653 | A04 | 5305611 | 1.17E-06 | 0.29002 |  | Bn-scaff_22728_1-p344206 | C03 | 6167915 | 3.40E-06 | 0.29867 |
|  | Bn-A04-p5982123 | A04 | 7177075 | 3.11E-06 | 0.30088 |  | Bn-scaff_16517_1-p63354 | C04 | 244113 | 1.12E-06 | 0.29104 |
|  | Bn-A04-p7077586 | A04 | 8348735 | 9.70E-07 | 0.29449 |  | Bn-scaff_24979_1-p132892 | C04 | 401792 | 4.67E-07 | 0.3123 |
|  | Bn-A04-p7275318 | A04 | 8562276 | 9.70E-07 | 0.29449 |  | Bn-scaff_16447_1-p403240 | C04 | 8636542 | 2.85E-07 | 0.32447 |
|  | Bn-A04-p7323275 | A04 | 8622635 | 2.88E-06 | 0.26836 |  | Bn-scaff_17869_1-p121828 | C04 | 9140900 | 3.13E-06 | 0.30076 |
|  | Bn-A04-p9098709 | A04 | 10190551 | 3.35E-08 | 0.33017 |  | Bn-scaff_20079_1-p69594 | C04 | 40880727 | 9.70E-07 | 0.29449 |
|  | Bn-A04-p9847875 | A04 | 10979452 | 2.71E-06 | 0.26986 |  | Bn-scaff_22527_1-p88113 | C05 | 5129170 | 1.98E-06 | 0.31228 |
|  | Bn-A04-p9932606 | A04 | 11068399 | 1.45E-06 | 0.32015 |  | Bn-scaff_20294_1-p268948 | C06 | 324445 | 4.74E-06 | 0.29039 |
|  | Bn-A04-p10188827 | A04 | 11333029 | 1.81E-07 | 0.29001 |  | Bn-scaff_17721_1-p50204 | C07 | 18852041 | 3.86E-06 | 0.29549 |
|  | Bn-A04-p10434805 | A04 | 11594670 | 5.08E-07 | 0.31025 |  | Bn-scaff_16110_1-p2044246 | C07 | 42827778 | 1.91E-06 | 0.31316 |
|  | Bn-A04-p11897079 | A04 | 12919880 | 5.45E-07 | 0.30854 |  | Bn-scaff_16110_1-p2030050 | C07 | 42846308 | 6.54E-07 | 0.30408 |
|  | Bn-A04-p13129306 | A04 | 13895032 | 3.85E-06 | 0.29557 |  | Bn-scaff_16110_1-p2028032 | C07 | 42848328 | 6.54E-07 | 0.30408 |
|  | Bn-A04-p13583325 | A04 | 14254894 | 2.48E-06 | 0.30662 |  | Bn-scaff_16110_1-p471808 | C07 | 44430478 | 4.76E-06 | 0.29026 |
|  | Bn-A04-p15514869 | A04 | 15938732 | 4.24E-06 | 0.29313 |  | Bn-scaff_16456_1-p453404 | C09 | 35037966 | 4.10E-06 | 0.29398 |
|  | Bn-scaff_16888_1-p1431260 | A04 | 16839984 | 1.13E-06 | 0.29074 | Myricetin | Bn-A03-p6299523 | A03 | 5649456 | 2.20E-06 | 0.231 |
|  | Bn-A04-p7322000 | A04 | 33629786 | 9.70E-07 | 0.29449 |  | Bn-A03-p6439432 | A03 | 5754736 | 3.63E-06 | 0.26052 |
|  | Bn-A05-p7370869 | A05 | 5235427 | 4.37E-06 | 0.29239 | TPC | Bn-A01-p8418102 | A01 | 7615643 | 7.48E-04 | 0.14143 |
|  | Bn-scaff_23096_1-p257017 | A05 | 11560893 | 1.81E-07 | 0.29001 |  | Bn-A01-p8452366 | A01 | 7669625 | 7.48E-04 | 0.14143 |
|  | Bn-A05-p16338133 | A05 | 15041844 | 5.60E-07 | 0.30787 |  | Bn-A01-p8452981 | A01 | 7727474 | 7.48E-04 | 0.14143 |
|  | Bn-A05-p21193491 | A05 | 19346126 | 4.05E-06 | 0.2943 | RSA | Bn-A02-p23309610 | A02 | 21418682 | 2.61E-04 | 0.16589 |
|  | Bn-A05-p21233666 | A05 | 19395677 | 3.99E-06 | 0.2947 |  | Bn-A05-p23188259 | A05 | 22815857 | 4.72E-04 | 0.15308 |
|  | Bn-A05-p21299910 | A05 | 19464223 | 1.81E-07 | 0.29001 |  | Bn-A05-p23196656 | A05 | 2515722 | 2.61E-04 | 0.16589 |
|  | Bn-A05-p21319243 | A05 | 19480513 | 1.15E-06 | 0.29046 |  | Bn-scaff_23907_1-p819330 | C04 | 1352000 | 6.86E-04 | 0.1451 |
|  | Bn-A05-p21326091 | A05 | 19484238 | 1.06E-06 | 0.29226 | TFDC | Bn-A05-p1693000 | A05 | 1837985 | 6.73E-04 | 0.14592 |
|  | Bn-A05-p21852563 | A05 | 19994324 | 4.41E-06 | 0.29218 |  | Bn-A05-p1693196 | A05 | 16381092 | 6.73E-04 | 0.14592 |
|  | Bn-A06-p1312619 | A06 | 1350492 | 1.13E-06 | 0.29089 |  | Bn-A05-p1695394 | A05 | 16421645 | 6.73E-04 | 0.14592 |
|  | Bn-A06-p1403969 | A06 | 1441858 | 1.81E-07 | 0.29001 |  | Bn-A05-p1695552 | A05 | 16425855 | 6.73E-04 | 0.14592 |
|  | Bn-A06-p3051799 | A06 | 2944746 | 1.81E-07 | 0.29001 |  | Bn-A05-p1696330 | A05 | 16447217 | 5.02E-04 | 0.15219 |
|  | Bn-A06-p3066372 | A06 | 2959161 | 1.81E-07 | 0.29001 |  | Bn-A05-p1696411 | A05 | 16463413 | 5.02E-04 | 0.15219 |
|  | Bn-A06-p22324968 | A06 | 21356613 | 4.02E-06 | 0.29447 |  | Bn-A05-p1697018 | A05 | 16514073 | 2.70E-04 | 0.16564 |
|  | Bn-A07-p10010327 | A07 | 107230 | 9.16E-07 | 0.29587 |  | Bn-A05-p1764530 | A05 | 17025636 | 1.64E-04 | 0.17654 |
|  | Bn-A07-p9689917 | A07 | 156817 | 4.63E-06 | 0.29096 |  | Bn-A05-p1775540 | A05 | 17078398 | 1.64E-04 | 0.17654 |
| Caffeic acid | Bn-A07-p9690172 | A07 | 156819 | 1.06E-06 | 0.29224 |  | Bn-scaff_15705_1-p374299 | A06 | 19362705 | 3.13E-04 | 0.16245 |
|  | Bn-A07-p9690345 | A07 | 156821 | 1.06E-06 | 0.29224 | TFLC | Bn-A04-p11988057 | A04 | 13192688 | 9.55E-04 | 0.1223 |
|  | Bn-A07-p9324529 | A07 | 157863 | 1.06E-06 | 0.29224 |  | Bn-A04-p12010066 | A04 | 13198156 | 9.55E-04 | 0.1223 |
|  | Bn-A07-p8851644 | A07 | 975001 | 1.05E-06 | 0.29252 |  | Bn-A06-p21800927 | A06 | 20820985 | 9.58E-04 | 0.12224 |
|  | Bn-A07-p1729364 | A07 | 1812785 | 1.58E-06 | 0.31798 |  | Bn-A06-p22077748 | A06 | 2320596 | 2.69E-04 | 0.1464 |
|  | Bn-A07-p8348770 | A07 | 9795581 | 1.17E-06 | 0.29003 |  | Bn-A10-p12957851 | A10 | 12986214 | 6.87E-04 | 0.1285 |
|  | Bn-A07-p8615521 | A07 | 10060370 | 3.09E-06 | 0.30108 |  | Bn-scaff_19248_1-p57521 | C04 | 6618050 | 8.59E-04 | 0.1243 |
|  | Bn-A07-p8616202 | A07 | 10061050 | 4.18E-06 | 0.29354 |  | Bn-scaff_19248_1-p64918 | C04 | 6621616 | 8.59E-04 | 0.1243 |
|  | Bn-A07-p8617442 | A07 | 10062333 | 1.81E-07 | 0.29001 |  | Bn-scaff_19248_1-p66490 | C04 | 6623177 | 8.59E-04 | 0.1243 |
|  | Bn-A07-p8775069 | A07 | 10226485 | 3.34E-06 | 0.29916 |  | Bn-scaff_19248_1-p66592 | C04 | 6623279 | 8.59E-04 | 0.1243 |
|  | Bn-A07-p8977017 | A07 | 10442232 | 1.15E-06 | 0.29041 |  | Bn-scaff_19248_1-p73640 | C04 | 6627161 | 8.59E-04 | 0.1243 |
|  | Bn-A07-p9014912 | A07 | 10473737 | 9.72E-07 | 0.29443 | TAC | Bn-scaff_18917_1-p653997 | C03 | 29082938 | 3.70E-04 | 0.15778 |
|  | Bn-A07-p9708357 | A07 | 10995667 | 1.06E-06 | 0.29224 |  | Bn-scaff_21786_1-p158103 | C08 | 19676215 | 5.18E-04 | 0.15053 |
|  | Bn-A07-p9975543 | A07 | 11250817 | 1.06E-06 | 0.29224 | AAC | Bn-A03-p9966625 | A03 | 9165370 | 6.96E-04 | 0.14244 |
|  | Bn-A07-p12830017 | A07 | 14921504 | 1.17E-06 | 0.29004 |  | Bn-scaff_16445_1-p2613244 | C08 | 34299868 | 2.58E-04 | 0.16344 |

MLM, TPC, RSA, TFDC, TFLC, TAC, and AAC were the abbreviations of mixed linear model, total phenolic content, radical scavenging activity, total flavonoid content, total flavonol content, total anthocyanin content, and ascorbic acid content, respectively.
