# Supplementary material for: Integration of genome-wide association studies, metabolomics, and transcriptomics reveals phenolic acid- and flavonoid-associated genes and their regulatory elements under drought stress in rapeseed flowers
Source: Front Plant Sci. 2024 Jan 11;14:1249142. doi: 10.3389/fpls.2023.1249142 (PMC10808681; doi:10.3389/fpls.2023.1249142)
Supplement: Supplementary file 13 [file DataSheet_13.docx]

| Supplementary Table S8. Significantly associated SNP markers with phenolic compounds at *P* < 0.00001 analyzed through MLM based statistical model of 119 rapeseed (*Brassica napus*) accessions under drought stress condition. | | | | | | | | | | | |
| --- | --- | --- | --- | --- | --- | --- | --- | --- | --- | --- | --- |
| Phenolic compounds | SNP marker | Linkage group | Position (bp) | *P*-value | *R^2^* (%) | Metabolite | SNP marker | Linkage group | Position (bp) | *P*-value | *R^2^* (%) |
| Epicatechin | Bn-A01-p24840306 | A01 | 28789300 | 1.24E-06 | 0.32437 | Myricetin | Bn-A04-p10541268 | A04 | 13727498 | 2.25E-07 | 0.35019 |
|  | Bn-A02-p12520861 | A02 | 30748459 | 1.19E-06 | 0.28957 |  | Bn-A04-p15475759 | A04 | 20602376 | 3.99E-07 | 0.33589 |
|  | Bn-A02-p19317643 | A02 | 48610326 | 3.73E-06 | 0.29649 |  | Bn-A04-p9174273 | A04 | 29735454 | 3.56E-07 | 0.33867 |
|  | Bn-A03-p10600567 | A03 | 21736504 | 3.87E-06 | 0.29556 |  | Bn-A04-p10608772 | A04 | 36674359 | 2.17E-07 | 0.351 |
|  | Bn-A03-p25008908 | A03 | 27777397 | 1.95E-06 | 0.31282 |  | Bn-A04-p12051277 | A04 | 38666357 | 4.85E-07 | 0.33107 |
|  | Bn-A03-p18405869 | A03 | 35797438 | 2.12E-06 | 0.31071 |  | Bn-A04-p17239509 | A04 | 40629305 | 4.75E-07 | 0.33158 |
|  | Bn-A04-p14026143 | A04 | 25622396 | 1.31E-06 | 0.32288 |  | Bn-A04-p2753796 | A04 | 41603428 | 4.31E-07 | 0.33398 |
|  | Bn-A04-p13761897 | A04 | 35730314 | 5.14E-06 | 0.28847 |  | Bn-A04-p2949339 | A04 | 49700351 | 3.21E-07 | 0.3413 |
|  | Bn-A04-p13767868 | A04 | 38679311 | 5.14E-06 | 0.28847 |  | Bn-A04-p3140726 | A04 | 63745313 | 4.34E-07 | 0.3338 |
|  | Bn-scaff_16517_1-p561851 | A04 | 60736356 | 1.25E-06 | 0.2884 |  | Bn-scaff_16984_1-p299228 | A05 | 17755360 | 1.40E-07 | 0.36209 |
|  | Bn-A04-p14353463 | A04 | 61674476 | 1.25E-06 | 0.2884 |  | Bn-A05-p19583714 | A05 | 35761408 | 4.81E-07 | 0.33124 |
|  | Bn-A04-p14060096 | A04 | 69672426 | 1.25E-06 | 0.2884 |  | Bn-A05-p5973528 | A05 | 39629424 | 4.72E-07 | 0.33174 |
|  | Bn-A05-p16664411 | A05 | 58805388 | 1.20E-06 | 0.32529 |  | Bn-A05-p9347113 | A05 | 41681485 | 1.72E-07 | 0.35684 |
|  | Bn-A06-p24678077 | A06 | 21669344 | 3.22E-06 | 0.30019 |  | Bn-A05-p18615644 | A05 | 47809368 | 3.62E-07 | 0.33831 |
|  | Bn-A07-p18233390 | A07 | 28726499 | 9.98E-07 | 0.29392 |  | Bn-A05-p19806333 | A05 | 49720355 | 4.19E-07 | 0.33467 |
|  | Bn-A08-p3959828 | A08 | 17743400 | 3.08E-06 | 0.30133 |  | Bn-A05-p20903214 | A05 | 51763329 | 4.46E-07 | 0.33311 |
|  | Bn-A08-p6185551 | A08 | 36804447 | 3.71E-06 | 0.29664 |  | Bn-A05-p18616260 | A05 | 54758449 | 3.62E-07 | 0.33831 |
|  | Bn-A10-p5873429 | A10 | 24738460 | 6.48E-06 | 0.28273 |  | Bn-A05-p6423355 | A05 | 57716400 | 4.70E-07 | 0.33182 |
|  | Bn-scaff_15808_1-p388294 | A10 | 50751452 | 4.51E-06 | 0.29173 |  | Bn-A05-p6404595 | A05 | 60611361 | 3.83E-07 | 0.33686 |
|  | Bn-A10-p8696396 | A10 | 61698313 | 1.13E-06 | 0.32671 |  | Bn-A05-p10820194 | A05 | 61622414 | 2.81E-07 | 0.3446 |
|  | Bn-scaff_19364_1-p230722 | C01 | 52661467 | 7.58E-06 | 0.27884 |  | Bn-A05-p21248720 | A05 | 65700477 | 4.39E-07 | 0.33354 |
|  | Bn-scaff_16298_1-p75507 | C02 | 42803392 | 5.35E-06 | 0.28749 |  | Bn-A05-p22454211 | A05 | 72773358 | 1.33E-07 | 0.36334 |
|  | Bn-scaff_21131_1-p167685 | C02 | 69608464 | 5.35E-06 | 0.28749 |  | Bn-A06-p7420466 | A06 | 25658407 | 3.08E-07 | 0.34229 |
|  | Bn-scaff_18917_1-p741437 | C03 | 36764387 | 1.83E-06 | 0.31441 |  | Bn-A06-p25369364 | A06 | 42731377 | 3.59E-07 | 0.33853 |
|  | Bn-scaff_16804_2-p157923 | C04 | 54614425 | 1.25E-06 | 0.2884 |  | Bn-A06-p5226816 | A06 | 42785352 | 4.25E-07 | 0.33432 |
|  | Bn-scaff_25595_1-p414764 | C05 | 43670426 | 2.10E-07 | 0.28664 |  | Bn-A06-p6555456 | A06 | 43618352 | 2.83E-07 | 0.34439 |
|  | Bn-scaff_16197_1-p135078 | C08 | 38774386 | 1.61E-06 | 0.31777 |  | Bn-A06-p1743446 | A06 | 43739339 | 1.01E-07 | 0.37039 |
|  | Bn-scaff_15808_1-p487486 | C09 | 52702475 | 4.51E-06 | 0.29173 |  | Bn-A06-p18709596 | A06 | 44686326 | 4.69E-07 | 0.33186 |
|  | Bn-scaff_16912_1-p28404 | C09 | 63810360 | 5.49E-07 | 0.3453 |  | Bn-A06-p5301714 | A06 | 45793404 | 4.25E-07 | 0.33432 |
| Chlorogenic acid | Bn-A01-p739098 | A01 | 23755510 | 8.45E-07 | 0.33593 |  | Bn-A06-p14212186 | A06 | 52664386 | 4.16E-07 | 0.33487 |
|  | Bn-A01-p6333910 | A01 | 34767483 | 2.69E-06 | 0.30631 |  | Bn-A06-p3428214 | A06 | 66606464 | 1.95E-07 | 0.3537 |
|  | Bn-A01-p10344498 | A01 | 45782365 | 2.55E-06 | 0.30764 |  | Bn-A07-p12400157 | A07 | 17627322 | 4.60E-07 | 0.33236 |
|  | Bn-A01-p6958863 | A01 | 59651471 | 2.79E-06 | 0.30539 |  | Bn-A07-p15905994 | A07 | 53715398 | 4.38E-07 | 0.33358 |
|  | Bn-A01-p14914475 | A01 | 59682342 | 2.88E-06 | 0.30459 |  | Bn-A07-p15949460 | A07 | 69634476 | 3.11E-07 | 0.34203 |
|  | Bn-A01-p11642772 | A01 | 60699345 | 2.71E-06 | 0.3061 |  | Bn-A08-p13427247 | A08 | 10698314 | 2.55E-07 | 0.34699 |
|  | Bn-A01-p23096147 | A01 | 74788337 | 1.31E-06 | 0.32471 |  | Bn-A08-p13382119 | A08 | 18650339 | 2.40E-07 | 0.34851 |
|  | Bn-A02-p25457843 | A02 | 68698375 | 1.76E-06 | 0.31715 |  | Bn-A08-p13427290 | A08 | 23809392 | 3.23E-07 | 0.34109 |
|  | Bn-A03-p24626484 | A03 | 24605470 | 2.41E-06 | 0.30912 |  | Bn-A08-p19800532 | A08 | 28807358 | 4.65E-07 | 0.3321 |
|  | Bn-A03-p7789818 | A03 | 26677470 | 6.29E-07 | 0.30673 |  | Bn-A08-p19802597 | A08 | 39618489 | 4.65E-07 | 0.3321 |
|  | Bn-A03-p24606883 | A03 | 33751393 | 1.75E-06 | 0.31726 |  | Bn-A08-p19799335 | A08 | 40784373 | 4.65E-07 | 0.3321 |
|  | Bn-A03-p9011966 | A03 | 37642309 | 1.19E-06 | 0.32712 |  | Bn-A08-p13460499 | A08 | 47773325 | 3.23E-07 | 0.34109 |
|  | Bn-A03-p24526574 | A03 | 37797376 | 1.52E-06 | 0.32089 |  | Bn-A08-p18463273 | A08 | 52655388 | 3.32E-07 | 0.34045 |
|  | Bn-A03-p24669270 | A03 | 47681345 | 1.73E-06 | 0.31752 |  | Bn-A08-p13692951 | A08 | 58691330 | 4.83E-07 | 0.33115 |
|  | Bn-scaff_17521_1-p805361 | A03 | 52700300 | 2.56E-06 | 0.30757 |  | Bn-A09-p11487820 | A09 | 17618473 | 1.97E-07 | 0.35347 |
|  | Bn-A03-p24612593 | A03 | 65635392 | 1.52E-06 | 0.32089 |  | Bn-A09-p11544785 | A09 | 20768341 | 1.97E-07 | 0.35347 |
|  | Bn-A04-p4035141 | A04 | 13730336 | 1.42E-06 | 0.32262 |  | Bn-A09-p19835714 | A09 | 35750413 | 4.03E-07 | 0.33561 |
|  | Bn-A04-p10671721 | A04 | 39665434 | 8.74E-07 | 0.33505 |  | Bn-scaff_17371_1-p462701 | A09 | 47615423 | 1.95E-07 | 0.35377 |
|  | Bn-A04-p10602815 | A04 | 49718487 | 1.20E-06 | 0.32687 |  | Bn-A09-p11110730 | A09 | 48767377 | 1.97E-07 | 0.35347 |
|  | Bn-A05-p14599830 | A05 | 38740490 | 3.23E-06 | 0.30167 |  | Bn-A09-p16846786 | A09 | 50743505 | 3.94E-07 | 0.33621 |
|  | Bn-A05-p13541711 | A05 | 42661316 | 3.08E-06 | 0.30283 |  | Bn-A09-p28802268 | A09 | 63766478 | 2.88E-07 | 0.34395 |
|  | Bn-A05-p17514353 | A05 | 51735300 | 3.14E-06 | 0.30238 |  | Bn-A09-p11210109 | A09 | 65742425 | 1.97E-07 | 0.35347 |
|  | Bn-A06-p3437409 | A06 | 12615333 | 3.12E-06 | 0.30252 |  | Bn-A09-p11141987 | A09 | 65760372 | 1.97E-07 | 0.35347 |
|  | Bn-A06-p3532353 | A06 | 12732383 | 2.98E-06 | 0.30374 |  | Bn-A09-p28225347 | A09 | 66786494 | 4.65E-07 | 0.33208 |
|  | Bn-A06-p3839293 | A06 | 13700378 | 2.80E-06 | 0.3053 |  | Bn-A09-p36550750 | A09 | 70758340 | 3.86E-07 | 0.33671 |
|  | Bn-A06-p3434498 | A06 | 16758504 | 3.12E-06 | 0.30252 |  | Bn-A09-p11139874 | A09 | 72781337 | 1.97E-07 | 0.35347 |
|  | Bn-A06-p3444868 | A06 | 24742430 | 2.65E-06 | 0.30668 |  | Bn-A09-p2548356 | A09 | 74775437 | 3.80E-07 | 0.33707 |
|  | Bn-A06-p3625328 | A06 | 28636469 | 2.80E-06 | 0.3053 |  | Bn-scaff_24869_1-p147699 | C01 | 13770481 | 1.95E-07 | 0.35372 |
|  | Bn-A06-p26186229 | A06 | 32665450 | 7.20E-07 | 0.30342 |  | Bn-scaff_22790_1-p809369 | C01 | 27681308 | 4.67E-07 | 0.332 |
|  | Bn-A06-p3528559 | A06 | 35652485 | 2.91E-06 | 0.30427 |  | Bn-scaff_22790_1-p752821 | C01 | 38720362 | 4.20E-07 | 0.33458 |
|  | Bn-A06-p3528628 | A06 | 44724378 | 2.98E-06 | 0.30374 |  | Bn-scaff_22790_1-p777045 | C01 | 54654486 | 4.20E-07 | 0.33458 |
|  | Bn-A06-p3667300 | A06 | 50772452 | 2.65E-06 | 0.30671 |  | Bn-scaff_17369_1-p3394 | C01 | 72795411 | 1.24E-07 | 0.36503 |
|  | Bn-scaff_20901_1-p188137 | A06 | 60602460 | 3.12E-06 | 0.30252 |  | Bn-scaff_17522_1-p24269 | C02 | 10687469 | 4.48E-07 | 0.33303 |
|  | Bn-A06-p4337931 | A06 | 61785467 | 1.82E-06 | 0.31624 |  | Bn-scaff_16704_1-p268160 | C02 | 10733459 | 2.64E-07 | 0.34619 |
|  | Bn-scaff_20901_1-p58099 | A06 | 74616302 | 2.00E-06 | 0.31385 |  | Bn-scaff_15712_2-p74031 | C02 | 11609329 | 1.81E-07 | 0.35559 |
|  | Bn-A07-p16659232 | A07 | 10663433 | 5.99E-07 | 0.30795 |  | Bn-scaff_16449_1-p40101 | C02 | 11733509 | 4.62E-07 | 0.33224 |
|  | Bn-A07-p9347254 | A07 | 11754472 | 3.04E-06 | 0.30322 |  | Bn-scaff_15839_1-p190523 | C02 | 14601358 | 3.54E-07 | 0.33886 |
|  | Bn-A07-p6542812 | A07 | 20717392 | 7.06E-07 | 0.30392 |  | Bn-scaff_17289_1-p442449 | C02 | 14788436 | 4.23E-07 | 0.33446 |
|  | Bn-A07-p17411599 | A07 | 23686492 | 2.09E-06 | 0.31268 |  | Bn-scaff_21705_1-p470585 | C02 | 16621460 | 4.62E-07 | 0.33224 |
|  | Bn-A07-p17419179 | A07 | 32662312 | 2.09E-06 | 0.31268 |  | Bn-scaff_20942_1-p432654 | C02 | 18763390 | 2.77E-07 | 0.3449 |
|  | Bn-A07-p9339366 | A07 | 34808412 | 7.29E-07 | 0.30314 |  | Bn-scaff_16130_1-p689480 | C02 | 19616307 | 1.40E-07 | 0.36214 |
|  | Bn-A07-p17418824 | A07 | 35759435 | 5.18E-07 | 0.31153 |  | Bn-scaff_16704_1-p460727 | C02 | 19669493 | 2.95E-07 | 0.34341 |
|  | Bn-A07-p17404116 | A07 | 52690345 | 7.12E-07 | 0.3037 |  | Bn-scaff_21705_1-p608100 | C02 | 19721386 | 4.62E-07 | 0.33224 |
|  | Bn-A07-p9485484 | A07 | 72701363 | 2.05E-06 | 0.31322 |  | Bn-scaff_21705_1-p590008 | C02 | 22620484 | 4.62E-07 | 0.33224 |
|  | Bn-A08-p17513789 | A08 | 21707468 | 7.79E-07 | 0.3015 |  | Bn-scaff_16704_1-p348398 | C02 | 23651403 | 2.95E-07 | 0.34341 |
|  | Bn-A08-p17462017 | A08 | 24650509 | 3.24E-06 | 0.30162 |  | Bn-scaff_21705_1-p612632 | C02 | 24770432 | 4.62E-07 | 0.33224 |
|  | Bn-A08-p17254975 | A08 | 27656496 | 2.05E-06 | 0.31323 |  | Bn-scaff_16704_1-p268272 | C02 | 24800396 | 2.64E-07 | 0.34619 |
|  | Bn-A08-p17108442 | A08 | 30678330 | 7.61E-07 | 0.30209 |  | Bn-scaff_21705_1-p494559 | C02 | 31615308 | 4.62E-07 | 0.33224 |
|  | Bn-A08-p17130151 | A08 | 31651401 | 3.07E-06 | 0.30297 |  | Bn-scaff_16704_1-p459651 | C02 | 31660302 | 2.95E-07 | 0.34341 |
|  | Bn-A08-p17202584 | A08 | 42646399 | 2.63E-06 | 0.30682 |  | Bn-scaff_17079_1-p144750 | C02 | 32606379 | 2.14E-07 | 0.35135 |
|  | Bn-A08-p17192841 | A08 | 47671499 | 2.79E-06 | 0.30534 |  | Bn-scaff_16130_1-p733651 | C02 | 33703452 | 3.16E-07 | 0.3417 |
|  | Bn-A08-p17111194 | A08 | 47762323 | 3.07E-06 | 0.30297 |  | Bn-scaff_15712_6-p1200159 | C02 | 38668454 | 1.81E-07 | 0.35559 |
|  | Bn-A08-p17124630 | A08 | 58777455 | 7.57E-07 | 0.3022 |  | Bn-scaff_21705_1-p597650 | C02 | 38756349 | 4.62E-07 | 0.33224 |
|  | Bn-scaff_16793_1-p143675 | A08 | 71697380 | 1.19E-07 | 0.30146 |  | Bn-scaff_17077_1-p149538 | C02 | 44647485 | 1.85E-07 | 0.35503 |
|  | Bn-A09-p27531548 | A09 | 11727481 | 1.19E-07 | 0.30146 |  | Bn-scaff_17289_1-p458549 | C02 | 45663510 | 4.26E-07 | 0.33426 |
|  | Bn-A09-p4286317 | A09 | 14750341 | 2.89E-06 | 0.30446 |  | Bn-scaff_16704_1-p349056 | C02 | 46750479 | 2.95E-07 | 0.34341 |
|  | Bn-A09-p27607559 | A09 | 21761377 | 2.54E-06 | 0.30771 |  | Bn-scaff_17079_1-p21817 | C02 | 48785325 | 1.40E-07 | 0.36214 |
|  | Bn-A09-p8783204 | A09 | 29699481 | 1.19E-07 | 0.30146 |  | Bn-scaff_17079_1-p144459 | C02 | 49603351 | 2.14E-07 | 0.35135 |
|  | Bn-A09-p27579758 | A09 | 35712318 | 1.19E-07 | 0.30146 |  | Bn-scaff_21705_1-p482169 | C02 | 50685402 | 4.62E-07 | 0.33224 |
|  | Bn-A09-p4291965 | A09 | 51622406 | 3.18E-06 | 0.30206 |  | Bn-scaff_20942_1-p440106 | C02 | 52768480 | 1.26E-07 | 0.36471 |
|  | Bn-A09-p27637967 | A09 | 60797465 | 2.54E-06 | 0.30771 |  | Bn-scaff_15712_2-p18850 | C02 | 54640350 | 1.81E-07 | 0.35559 |
|  | Bn-A09-p27529804 | A09 | 62646392 | 1.19E-07 | 0.30146 |  | Bn-scaff_16704_1-p462410 | C02 | 56681393 | 2.95E-07 | 0.34341 |
|  | Bn-A09-p21224256 | A09 | 65794350 | 7.58E-07 | 0.30217 |  | Bn-scaff_20942_1-p434655 | C02 | 56775447 | 2.77E-07 | 0.3449 |
|  | Bn-A09-p27109839 | A09 | 70797318 | 1.19E-07 | 0.30146 |  | Bn-scaff_15712_2-p109180 | C02 | 60743369 | 1.81E-07 | 0.35559 |
|  | Bn-A09-p27351028 | A09 | 71671398 | 1.19E-07 | 0.30146 |  | Bn-scaff_17079_1-p28593 | C02 | 62628403 | 1.40E-07 | 0.36214 |
|  | Bn-A09-p4288843 | A09 | 71726497 | 2.54E-06 | 0.30778 |  | Bn-scaff_17079_1-p21658 | C02 | 63618405 | 1.40E-07 | 0.36214 |
|  | Bn-A10-p8948483 | A10 | 11632400 | 1.31E-06 | 0.3246 |  | Bn-scaff_16704_1-p328255 | C02 | 66671357 | 3.12E-07 | 0.34202 |
|  | Bn-A10-p8960805 | A10 | 16788488 | 7.78E-07 | 0.30152 |  | Bn-scaff_16704_1-p461002 | C02 | 66719362 | 2.95E-07 | 0.34341 |
|  | Bn-A10-p8559413 | A10 | 16795308 | 2.95E-06 | 0.30398 |  | Bn-scaff_21705_1-p233028 | C02 | 66751475 | 4.82E-07 | 0.33119 |
|  | Bn-A10-p8960651 | A10 | 26653467 | 7.78E-07 | 0.30152 |  | Bn-scaff_17079_1-p145741 | C02 | 69749456 | 2.14E-07 | 0.35135 |
|  | Bn-A10-p8620702 | A10 | 42792367 | 7.53E-07 | 0.30234 |  | Bn-scaff_21705_1-p503703 | C02 | 69805396 | 4.62E-07 | 0.33224 |
|  | Bn-A10-p1458849 | A10 | 47787405 | 1.86E-06 | 0.3157 |  | Bn-scaff_16704_1-p272611 | C02 | 73678407 | 2.64E-07 | 0.34619 |
|  | Bn-A10-p8961465 | A10 | 50682484 | 1.14E-06 | 0.3283 |  | Bn-scaff_15877_1-p908058 | C03 | 14614456 | 2.49E-07 | 0.34761 |
|  | Bn-A10-p8953338 | A10 | 51674419 | 6.92E-07 | 0.3411 |  | Bn-scaff_17298_1-p281962 | C03 | 15755454 | 2.57E-07 | 0.34678 |
|  | Bn-A10-p8961320 | A10 | 51796340 | 1.14E-06 | 0.3283 |  | Bn-scaff_16394_2-p979621 | C03 | 17789403 | 1.39E-07 | 0.36221 |
|  | Bn-A10-p8964484 | A10 | 65779481 | 7.78E-07 | 0.30152 |  | Bn-scaff_21312_1-p169902 | C03 | 20671461 | 4.70E-07 | 0.33184 |
|  | Bn-A10-p9084421 | A10 | 66744427 | 2.99E-06 | 0.30366 |  | Bn-scaff_16755_1-p927921 | C03 | 25639463 | 7.65E-06 | 0.26463 |
|  | Bn-scaff_17036_1-p19202 | C01 | 26699319 | 1.67E-06 | 0.31836 |  | Bn-scaff_27313_1-p653 | C03 | 29789419 | 3.84E-07 | 0.33683 |
|  | Bn-scaff_23813_1-p522936 | C02 | 53623478 | 7.46E-07 | 0.30256 |  | Bn-scaff_16755_1-p902975 | C03 | 36651419 | 7.65E-06 | 0.26463 |
|  | Bn-scaff_23813_1-p531505 | C02 | 62781417 | 7.46E-07 | 0.30256 |  | Bn-scaff_16130_2-p484864 | C03 | 40752373 | 4.04E-07 | 0.33555 |
|  | Bn-scaff_22728_1-p1397442 | C03 | 27630373 | 2.09E-06 | 0.31274 |  | Bn-scaff_23761_1-p281604 | C03 | 41668496 | 3.05E-07 | 0.34254 |
|  | Bn-scaff_16352_1-p762688 | C03 | 70789477 | 7.20E-07 | 0.30343 |  | Bn-scaff_23954_1-p157687 | C03 | 43645448 | 3.00E-07 | 0.34296 |
|  | Bn-scaff_15911_1-p592398 | C04 | 30731392 | 2.44E-06 | 0.3088 |  | Bn-scaff_16394_2-p383438 | C03 | 45787340 | 9.55E-08 | 0.37176 |
|  | Bn-scaff_15908_1-p755460 | C04 | 41674363 | 7.50E-07 | 0.339 |  | Bn-scaff_19047_1-p10848 | C03 | 47659393 | 1.99E-07 | 0.35325 |
|  | Bn-scaff_15798_1-p121402 | C04 | 49625329 | 2.43E-06 | 0.3089 |  | Bn-scaff_17298_1-p306441 | C03 | 49724354 | 2.24E-07 | 0.35024 |
|  | Bn-scaff_15798_1-p1081921 | C04 | 62736433 | 2.96E-06 | 0.30384 |  | Bn-scaff_28562_1-p30677 | C03 | 49738402 | 3.98E-07 | 0.33594 |
|  | Bn-scaff_25870_1-p48609 | C04 | 63633345 | 3.06E-06 | 0.30305 |  | Bn-scaff_28265_1-p20757 | C03 | 55666422 | 1.14E-07 | 0.36735 |
|  | Bn-scaff_20084_1-p106565 | C07 | 33737388 | 2.73E-06 | 0.30592 |  | Bn-scaff_16002_1-p2282681 | C03 | 59664430 | 1.34E-07 | 0.36308 |
|  | Bn-scaff_20084_1-p399365 | C07 | 44769340 | 7.49E-07 | 0.30247 |  | Bn-scaff_20646_1-p492384 | C03 | 59670459 | 4.59E-07 | 0.33241 |
| Caffeic acid | Bn-A05-p14332800 | A05 | 15634349 | 7.60E-06 | 0.2827 |  | Bn-scaff_16002_1-p1595246 | C03 | 59746490 | 2.70E-07 | 0.3456 |
|  | Bn-A05-p6271443 | A05 | 61673335 | 5.23E-06 | 0.25801 |  | Bn-scaff_20646_1-p226900 | C03 | 60804394 | 3.38E-07 | 0.33997 |
|  | Bn-A09-p1404415 | A09 | 11806350 | 7.95E-06 | 0.24811 |  | Bn-scaff_20646_1-p210307 | C03 | 61645313 | 3.38E-07 | 0.33997 |
|  | Bn-A09-p5274560 | A09 | 69805384 | 7.31E-07 | 0.30574 |  | Bn-scaff_28562_1-p23696 | C03 | 69778480 | 3.98E-07 | 0.33594 |
|  | Bn-scaff_15838_5-p603655 | C01 | 46706356 | 2.63E-06 | 0.30963 |  | Bn-scaff_16002_1-p2307317 | C03 | 72700395 | 3.76E-07 | 0.33732 |
| Coumaric acid | Bn-A01-p9258144 | A01 | 13708418 | 9.73E-08 | 0.39448 |  | Bn-scaff_28562_1-p22961 | C03 | 73738363 | 3.98E-07 | 0.33594 |
|  | Bn-A01-p9469933 | A01 | 20708470 | 1.76E-10 | 0.46865 |  | Bn-scaff_27676_1-p62035 | C04 | 14756363 | 4.48E-07 | 0.33302 |
|  | Bn-A01-p6333910 | A01 | 34767483 | 9.86E-06 | 0.27483 |  | Bn-scaff_19821_1-p337530 | C04 | 30789324 | 4.07E-07 | 0.33539 |
|  | Bn-A01-p6333810 | A01 | 44798502 | 2.45E-06 | 0.2748 |  | Bn-scaff_27421_1-p150153 | C04 | 46799320 | 1.60E-08 | 0.33085 |
|  | Bn-A01-p8439363 | A01 | 73646490 | 1.76E-10 | 0.46865 |  | Bn-scaff_23432_1-p43409 | C04 | 47723311 | 3.39E-07 | 0.33991 |
|  | Bn-A02-p12452799 | A02 | 10685324 | 7.63E-07 | 0.3032 |  | Bn-scaff_23432_1-p58237 | C04 | 57784389 | 3.39E-07 | 0.33991 |
|  | Bn-A02-p12044265 | A02 | 16769326 | 7.61E-07 | 0.30326 |  | Bn-scaff_17869_1-p110378 | C04 | 59722505 | 2.18E-08 | 0.40987 |
|  | Bn-A02-p11053817 | A02 | 38778377 | 5.51E-06 | 0.21496 |  | Bn-scaff_20817_1-p60579 | C04 | 60632495 | 3.77E-07 | 0.33729 |
|  | Bn-A02-p12460628 | A02 | 40662371 | 7.63E-07 | 0.3032 |  | Bn-scaff_27676_1-p175780 | C04 | 63600341 | 4.39E-07 | 0.33351 |
|  | Bn-A02-p12443767 | A02 | 40782505 | 3.03E-06 | 0.30448 |  | Bn-scaff_22530_1-p56354 | C04 | 71665479 | 3.39E-07 | 0.33991 |
|  | Bn-A02-p12393535 | A02 | 56747462 | 7.60E-07 | 0.30328 |  | Bn-scaff_16647_1-p434968 | C06 | 13617386 | 4.55E-07 | 0.33261 |
|  | Bn-A03-p21238466 | A03 | 12601333 | 3.39E-09 | 0.48799 |  | Bn-scaff_16647_1-p34631 | C06 | 17633499 | 4.74E-07 | 0.33163 |
|  | Bn-A03-p419250 | A03 | 16601451 | 1.36E-09 | 0.46865 |  | Bn-scaff_16984_1-p188161 | C06 | 26694449 | 2.28E-07 | 0.34976 |
|  | Bn-A03-p298309 | A03 | 40704419 | 5.89E-09 | 0.47218 |  | Bn-scaff_16647_1-p190518 | C06 | 28738317 | 4.07E-07 | 0.33539 |
|  | Bn-A03-p420642 | A03 | 41743320 | 5.76E-09 | 0.47283 |  | Bn-scaff_25107_1-p22878 | C06 | 38722391 | 1.56E-07 | 0.35942 |
|  | Bn-A03-p13572495 | A03 | 61768302 | 5.49E-09 | 0.47417 |  | Bn-scaff_16553_1-p87747 | C06 | 39615327 | 1.89E-07 | 0.35445 |
|  | Bn-A04-p12163967 | A04 | 69615422 | 1.38E-06 | 0.32465 |  | Bn-scaff_16984_1-p129923 | C06 | 45694361 | 1.54E-07 | 0.35973 |
|  | Bn-A06-p8697193 | A06 | 67799339 | 7.24E-06 | 0.24907 |  | Bn-scaff_16553_1-p87504 | C06 | 49608432 | 1.89E-07 | 0.35445 |
|  | Bn-A07-p14814393 | A07 | 71628481 | 5.21E-09 | 0.47568 |  | Bn-scaff_25107_1-p4106 | C06 | 53724346 | 1.56E-07 | 0.35942 |
|  | Bn-A10-p13577876 | A10 | 63608461 | 1.16E-06 | 0.32904 |  | Bn-scaff_25107_1-p4704 | C06 | 53747413 | 1.56E-07 | 0.35942 |
|  | Bn-A10-p13669875 | A10 | 66766446 | 3.30E-09 | 0.48875 |  | Bn-scaff_16984_1-p22409 | C06 | 56685310 | 1.56E-07 | 0.35938 |
|  | Bn-scaff_21820_1-p578364 | C01 | 64603489 | 6.29E-09 | 0.47031 |  | Bn-scaff_16647_1-p292154 | C06 | 64789405 | 4.51E-07 | 0.33285 |
|  | Bn-scaff_23408_1-p97029 | C05 | 15626414 | 2.01E-06 | 0.31498 |  | Bn-scaff_16647_1-p287095 | C06 | 66623446 | 4.51E-07 | 0.33285 |
|  | Bn-scaff_23408_1-p96806 | C05 | 30718429 | 2.09E-06 | 0.31389 |  | Bn-scaff_16647_1-p153105 | C06 | 66651489 | 4.74E-07 | 0.33164 |
|  | Bn-scaff_23408_1-p96976 | C05 | 35690496 | 2.01E-06 | 0.31498 |  | Bn-scaff_16647_1-p169144 | C06 | 67636476 | 4.07E-07 | 0.33539 |
|  | Bn-scaff_23408_1-p97372 | C05 | 69738427 | 2.09E-06 | 0.31389 |  | Bn-scaff_15818_2-p1207187 | C06 | 69773487 | 4.79E-07 | 0.33136 |
|  | Bn-scaff_16361_1-p2793418 | C08 | 49692309 | 1.59E-07 | 0.29592 |  | Bn-scaff_16984_1-p294576 | C06 | 70611495 | 1.40E-07 | 0.36209 |
|  | Bn-scaff_16174_1-p1445094 | C08 | 53674421 | 1.35E-09 | 0.46883 |  | Bn-scaff_16110_1-p3665685 | C07 | 12602350 | 3.49E-07 | 0.33921 |
|  | Bn-scaff_16770_1-p1746549 | C08 | 56760303 | 9.26E-10 | 0.47929 |  | Bn-scaff_16069_1-p4686169 | C07 | 12770457 | 3.78E-07 | 0.33721 |
|  | Bn-scaff_16174_1-p1444980 | C08 | 58624501 | 4.64E-07 | 0.31548 |  | Bn-scaff_16069_1-p4645442 | C07 | 16645463 | 4.40E-07 | 0.33346 |
|  | Bn-scaff_16770_1-p3966893 | C08 | 66654367 | 5.29E-07 | 0.31221 |  | Bn-scaff_16069_1-p4620976 | C07 | 20650330 | 3.82E-07 | 0.33695 |
|  | Bn-scaff_16770_1-p1924877 | C08 | 66702346 | 9.26E-10 | 0.47929 |  | Bn-scaff_16110_1-p3644698 | C07 | 23723483 | 3.49E-07 | 0.33921 |
| Myricetin | Bn-A01-p1439621 | A01 | 26766362 | 2.72E-07 | 0.34537 |  | Bn-scaff_16069_1-p1668600 | C07 | 31650355 | 3.74E-07 | 0.3375 |
|  | Bn-A01-p2053320 | A01 | 30694385 | 3.50E-07 | 0.33916 |  | Bn-scaff_16110_1-p3665779 | C07 | 36752391 | 3.49E-07 | 0.33921 |
|  | Bn-A01-p24829988 | A01 | 57643362 | 3.09E-07 | 0.34223 |  | Bn-scaff_16110_1-p3625554 | C07 | 37615498 | 3.49E-07 | 0.33921 |
|  | Bn-A01-p26461873 | A01 | 57705407 | 2.43E-07 | 0.34826 |  | Bn-scaff_16110_1-p3625372 | C07 | 40720481 | 3.49E-07 | 0.33921 |
|  | Bn-A01-p24829245 | A01 | 58601416 | 3.09E-07 | 0.34223 |  | Bn-scaff_16110_1-p3624627 | C07 | 44801445 | 3.49E-07 | 0.33921 |
|  | Bn-A01-p1449865 | A01 | 60725373 | 2.32E-07 | 0.34939 |  | Bn-scaff_15754_1-p240103 | C07 | 52653424 | 4.48E-07 | 0.33303 |
|  | Bn-A01-p1440009 | A01 | 72649424 | 2.54E-07 | 0.34708 |  | Bn-scaff_16069_1-p3780494 | C07 | 52777301 | 3.35E-07 | 0.34019 |
|  | Bn-A01-p10926129 | A01 | 73626377 | 9.75E-08 | 0.33391 |  | Bn-scaff_16069_1-p4833476 | C07 | 53691350 | 4.39E-07 | 0.33352 |
|  | Bn-A01-p24835306 | A01 | 73629403 | 2.89E-07 | 0.34392 |  | Bn-scaff_16130_1-p2299596 | C07 | 54663390 | 2.97E-07 | 0.34325 |
|  | Bn-A02-p978741 | A02 | 14626319 | 2.74E-07 | 0.34521 |  | Bn-scaff_16130_1-p2305762 | C07 | 55715348 | 2.97E-07 | 0.34325 |
|  | Bn-A02-p27111963 | A02 | 15601330 | 3.55E-07 | 0.33877 |  | Bn-scaff_16110_1-p3663531 | C07 | 61771362 | 3.49E-07 | 0.33921 |
|  | Bn-A02-p8799151 | A02 | 23802345 | 4.31E-07 | 0.33399 |  | Bn-scaff_16069_1-p1152134 | C07 | 67661317 | 3.78E-07 | 0.33721 |
|  | Bn-A02-p9409799 | A02 | 38618301 | 1.93E-07 | 0.35399 |  | Bn-scaff_16361_1-p1508732 | C08 | 64810338 | 4.78E-07 | 0.33142 |
|  | Bn-A02-p8912747 | A02 | 49716462 | 3.53E-07 | 0.33895 |  | Bn-scaff_15576_1-p69876 | C09 | 25684438 | 4.68E-07 | 0.33194 |
|  | Bn-A02-p27149888 | A02 | 58624405 | 4.67E-07 | 0.332 |  | Bn-scaff_17367_1-p63741 | C09 | 49601352 | 4.57E-07 | 0.3325 |
|  | Bn-A02-p24702986 | A02 | 65693380 | 1.70E-07 | 0.3571 |  | Bn-scaff_17077_1-p105753 | C09 | 72720376 | 2.50E-07 | 0.34752 |
|  | Bn-A02-p8571577 | A02 | 69663376 | 3.57E-07 | 0.33862 | RSA | Bn-A05-p23188259 | A05 | 21346528 | 4.87E-04 | 0.15095 |
|  | Bn-A02-p27110006 | A02 | 71644472 | 3.55E-07 | 0.33877 |  | Bn-A05-p23196656 | A05 | 21349945 | 1.90E-04 | 0.17117 |
|  | Bn-A03-p5039586 | A03 | 10792351 | 3.51E-07 | 0.33904 |  | Bn-scaff_16197_1-p372246 | C07 | 3534211 | 9.91E-04 | 0.13594 |
|  | Bn-A03-p28234586 | A03 | 18660490 | 2.18E-07 | 0.35095 |  | Bn-scaff_16197_1-p372490 | A09 | 3534018 | 9.91E-04 | 0.13594 |
|  | Bn-A03-p29271737 | A03 | 19747435 | 2.87E-07 | 0.34403 | TAC | Bn-scaff_16092_1-p476550 | C03 | 28153244 | 1.13E-04 | 0.17617 |
|  | Bn-A03-p17056395 | A03 | 20711467 | 2.47E-07 | 0.34784 |  | Bn-scaff_16092_1-p502841 | A03 | 28181511 | 1.13E-04 | 0.17617 |
|  | Bn-A03-p27420794 | A03 | 37663392 | 4.14E-07 | 0.33495 |  | Bn-scaff_16092_1-p541171 | C07 | 42948790 | 1.13E-04 | 0.17617 |
|  | Bn-A03-p29279909 | A03 | 40707483 | 2.87E-07 | 0.34403 |  | Bn-scaff_16092_1-p624909 | C07 | 28302227 | 1.13E-04 | 0.17617 |
| Myricetin | Bn-A03-p27406806 | A03 | 43712373 | 9.56E-08 | 0.33439 |  | Bn-scaff_16092_1-p646236 | C03 | 28323875 | 1.13E-04 | 0.17617 |
|  | Bn-A03-p18782416 | A03 | 61660336 | 2.07E-07 | 0.35224 |  | Bn-scaff_16092_1-p676443 | C07 | 2028358 | 1.13E-04 | 0.17617 |
|  | Bn-A03-p24745951 | A03 | 64706392 | 3.84E-07 | 0.33683 |  | Bn-scaff_16092_1-p676617 | C07 | 2028184 | 1.13E-04 | 0.17617 |
|  | Bn-scaff_16704_1-p392535 | A03 | 66736380 | 2.95E-07 | 0.34341 |  | Bn-scaff_16092_1-p703245 | C07 | 42591236 | 1.13E-04 | 0.17617 |
|  | Bn-A03-p20151244 | A03 | 69755362 | 2.80E-07 | 0.34468 | TFLC | Bn-A06-p22077748 | A06 | 21114139 | 7.29E-04 | 0.13415 |
|  | Bn-scaff_20646_1-p218671 | A03 | 71654411 | 4.23E-07 | 0.33442 |  | Bn-A10-p12957851 | A10 | 12986214 | 5.56E-04 | 0.13955 |
|  | Bn-A03-p25636300 | A03 | 72686484 | 1.65E-07 | 0.35796 |  |  |  |  |  |  |

MLM, RSA, TAC, and TFLC were the abbreviations of mixed linear model, radical scavenging activity, total anthocyanin content, and total flavonol content, respectively.
